# EfgA is a conserved formaldehyde sensor that leads to bacterial growth arrest in response to elevated formaldehyde

**DOI:** 10.1371/journal.pbio.3001208

**Published:** 2021-05-26

**Authors:** Jannell V. Bazurto, Dipti D. Nayak, Tomislav Ticak, Milya Davlieva, Jessica A. Lee, Chandler N. Hellenbrand, Leah B. Lambert, Olivia J. Benski, Caleb J. Quates, Jill L. Johnson, Jagdish Suresh Patel, F. Marty Ytreberg, Yousif Shamoo, Christopher J. Marx

**Affiliations:** 1 Department of Biological Sciences, University of Idaho, Moscow, Idaho, United States of America; 2 Institute for Modeling Collaboration and Innovation, University of Idaho, Moscow, Idaho, United States of America; 3 Institute for Bioinformatics and Evolutionary Studies, University of Idaho, Moscow, Idaho, United States of America; 4 Department of Plant and Microbial Biology, University of Minnesota, Twin Cities, Minnesota, United States of America; 5 Microbial and Plant Genomics Institute, University of Minnesota, Twin Cities, Minnesota, United States of America; 6 Biotechnology Institute, University of Minnesota, Twin Cities, Minnesota, United States of America; 7 Department of Organismic and Evolutionary Biology, Harvard University, Cambridge, Massachusetts, United States of America; 8 Department of Microbiology, University of Illinois, Urbana, Illinois, United States of America; 9 Department of Biosciences, Rice University, Houston, Texas, United States of America; 10 Space Biosciences Research Branch, NASA Ames Research Center, Moffett Field, California, United States of America; 11 Department of Physics, University of Idaho, Moscow, Idaho, United States of America; Max-Planck-Institut fur terrestrische Mikrobiologie, GERMANY

## Abstract

Normal cellular processes give rise to toxic metabolites that cells must mitigate. Formaldehyde is a universal stressor and potent metabolic toxin that is generated in organisms from bacteria to humans. Methylotrophic bacteria such as *Methylorubrum extorquens* face an acute challenge due to their production of formaldehyde as an obligate central intermediate of single-carbon metabolism. Mechanisms to sense and respond to formaldehyde were speculated to exist in methylotrophs for decades but had never been discovered. Here, we identify a member of the DUF336 domain family, named *efgA* for enhanced formaldehyde growth, that plays an important role in endogenous formaldehyde stress response in *M*. *extorquens* PA1 and is found almost exclusively in methylotrophic taxa. Our experimental analyses reveal that EfgA is a formaldehyde sensor that rapidly arrests growth in response to elevated levels of formaldehyde. Heterologous expression of EfgA in *Escherichia coli* increases formaldehyde resistance, indicating that its interaction partners are widespread and conserved. EfgA represents the first example of a formaldehyde stress response system that does not involve enzymatic detoxification. Thus, EfgA comprises a unique stress response mechanism in bacteria, whereby a single protein directly senses elevated levels of a toxic intracellular metabolite and safeguards cells from potential damage.

## Abstract 2

The known formaldehyde stress response systems involve enzymatic detoxification. Here, the authors show that the formaldehyde sensor efgA plays an important role in the endogenous formaldehyde stress response in Methylorubrum extorquens, halting cell growth in response to elevated levels of formaldehyde, and is found almost exclusively in methylotrophic taxa.

## Introduction

Robust organisms “maintain performance in the face of perturbations and uncertainty” [1] and require mechanisms for averting cellular damage during stress. In all biological systems, routine cellular processes generate highly toxic metabolites that can inflict damage on macromolecules and metabolites. The response systems that mitigate these endogenous stressors vary from detoxification systems, to neutralize reactive compounds such as hydrogen peroxide, imines, and aldehydes [2–5], to post-damage repair systems such as enzyme-mediated damage reversal [6,7] and targeted molecular degradation systems [8]. Induction of these responses can arise due to direct sensing of the metabolic toxins [9] or by sensing the damaged molecules themselves [6].

Formaldehyde is a ubiquitous metabolic toxin generated in most, if not all, organisms as a byproduct of enzymatic reactions or degradation products of metabolites. Due to its high reactivity with amines and thiols in particular, formaldehyde can damage numerous molecules such as metabolites [10], nucleic acids [11], and proteins [12–14]. To date, the only known formaldehyde-specific stress response systems involve enzymatic detoxification [3,15–17]. In bacteria, various formaldehyde detoxification pathways exist, including the widely conserved glutathione- (GSH-) dependent pathway as well as pathways dependent on pterins or sugar phosphates [3]; some species employ multiple pathways [18]. Thus far, there is a single example of a formaldehyde sensor, FrmR, a transcriptional repressor that directly binds formaldehyde and controls expression of the detoxification pathway in *Escherichia coli* [19].

Methylotrophs are diverse organisms that can use reduced one-carbon (C_1_) compounds (e.g., methane, methanol) or multicarbon compounds lacking carbon–carbon bonds (e.g., trimethylamine) as sole sources of carbon and energy. Methylotrophs are of practical importance in cycling C_1_ compounds like methane in the environment, consuming methylated compounds that affect microbiome–gut interactions [20–22] and converting C_1_ substrates to valuable products in industrial settings [23]. Due to their metabolic capacity, methylotrophs face the unique challenge of managing high fluxes of formaldehyde as a central metabolic intermediate.

**Fig 1:**
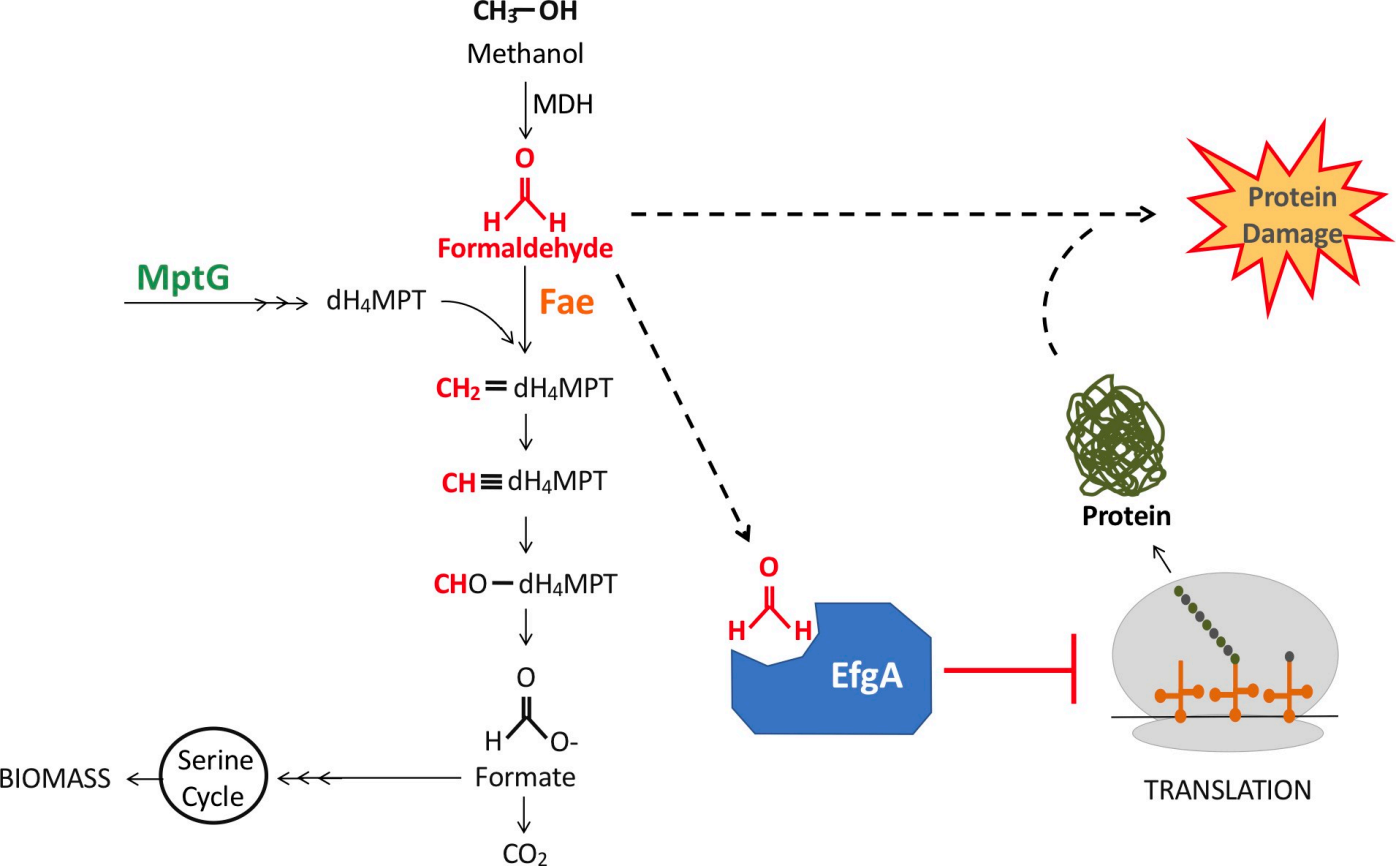
Methanol utilization pathway in *M*. *extorquens*. Methanol is oxidized to formaldehyde by MDH. Fae then condenses free formaldehyde and dH_4_MPT. MptG is required for dH_4_MPT biosynthesis. The pathway branches at formate which can be further oxidized to CO_2_ or routed to the assimilation pathways (e.g., serine cycle). Alternatively, free formaldehyde can bind EfgA and lead to cessation of translation. Our working model is that EfgA prevents formaldehyde-induced protein damage. dH_4_MPT, dephospho-tetrahydromethanopterin; Fae, formaldehyde-activating enzyme; MDH, methanol dehydrogenase.

Even for methylotrophs, where the metabolic pathways for the production and consumption of formaldehyde have been identified, no genes have been identified that allow cells to sense and respond to formaldehyde. The alphaproteobacterium *Methylorubrum extorquens* (formerly *Methylobacterium* [24]) is the most extensively studied facultative methylotroph. During growth on methanol, *M*. *extorquens* uses periplasmic methanol dehydrogenases to oxidize methanol to formaldehyde, which is presumed to diffuse through the inner membrane or possibly undergo active transport [25] (Fig 1). In the cytoplasm, formaldehyde condenses with the C_1_ carrier, dephospho-tetrahydromethanopterin (dH_4_MPT) [26,27], a reaction catalyzed by formaldehyde-activating enzyme (Fae, EC: 4.2.1.147) [28]. Through a series of dH_4_MPT intermediates, C_1_ units are oxidized to formate. This branchpoint metabolite is then either further oxidized to CO_2_ or assimilated into biomass via a tetrahydrofolate-linked pathway and, subsequently, the serine cycle [29,30]. The dH_4_MPT pathway plays the dual role of formaldehyde oxidation for C_1_ growth and detoxification, whereby mutants with a defective dH_4_MPT pathway exhibit methanol sensitivity due to an inability to detoxify formaldehyde produced intracellularly [26]. Indeed, at the level of gene expression, Fae and the enzymes of the dH_4_MPT pathway are present at high levels during growth on either C_1_ or multi-C growth substrates [31–33]. These data have led to the picture that methylotrophs, which can have intracellular fluxes of 100 mM/min [28] and an intracellular concentration of formaldehyde of approximately 1 mM [34] during growth on methanol, simply have sufficiently high, constitutive level of formaldehyde oxidation capacity to prevent toxic buildup, perhaps obviating formaldehyde sensing and an associated response.

Recent work demonstrating that formaldehyde stress tolerance is responsive to the environment suggests that cells can sense this toxic intermediate. Formaldehyde tolerance, defined here as the nongenetic ability for cells to grow in the presence of a maximum concentration of formaldehyde, was found to be phenotypically heterogeneous across genetically identical individuals of *M*. *extorquens* PA1, whereby some cells could not even survive exposure to 1 mM formaldehyde when it was provided in the growth medium [35]. This is consistent with the paradoxical finding that many methylotrophs are unable to directly grow on formaldehyde even though they rapidly generate it during growth on more reduced C_1_ compounds [26,35]. Although some of the cells in populations were surprisingly sensitive to formaldehyde, other rare cells could grow at normal growth rates under conditions where the vast majority rapidly die. The distribution of formaldehyde tolerances was found to rapidly shift upwards in response to formaldehyde stress and the distribution would relax downwards in its absence. Critically, differential death and growth could not explain the rapid shifts in the tolerance distribution, arguing that cells have yet undiscovered systems to sense and respond to formaldehyde and do not rely solely upon consistently high levels of dH_4_MPT pathway enzymes for resistance.

Here, we have employed experimental evolution to select for growth on formaldehyde and have uncovered multiple loci encoding genes that can impact formaldehyde resistance. Formaldehyde resistance is defined herein as the genetic-based ability for cells to grow in the presence of a maximum concentration of formaldehyde; relative tolerance/resistance is determined by the presence of growth and/or by the duration of the apparent lag times as formaldehyde has minimal impact, if any, on growth rates [35]. Experimental evolution has unique advantages compared to the classical approach of examining mutants defective in a process [36,37], including the ability to invoke the role of essential genes and gain-of-function mutations, both of which we observed herein. The simplest mechanistic hypothesis for increased resistance would be that—like antibiotic resistance mediated by enzymatic modification—evolved resistance would be mediated by an increase in formaldehyde oxidation. Thus, it came as a surprise that none of the loci with beneficial mutations are known to be related to formaldehyde oxidation or any other known methylotrophy gene [38]. Instead, we identified several novel loci, most commonly a gene of unknown function that we name *efgA* (**e**nhanced **f**ormaldehyde **g**rowth). We explored the role and function of EfgA through a combination of X-ray crystallography, molecular modeling, mutational analysis, and biochemical characterization, revealing that EfgA is a sensor that directly binds formaldehyde and rapidly arrests growth. EfgA is beneficial to cells when confronted with elevated levels of internally produced formaldehyde and, through phylogenetic analyses and heterologous expression, we show that EfgA function is broadly conserved in methylotrophs. Furthermore, EfgA-mediated formaldehyde protection is transferable to non-methylotrophs. Our findings represent the first characterized formaldehyde stress response in methylotrophs and demonstrate a unique strategy where a single protein senses a toxic metabolite and leads to growth arrest.

## Materials and methods

### Bacterial strains, media, and chemicals

*Methylorubrum* (reclassified from *Methylobacterium* [24]) strains used in this study are derived from *M*. *extorquens* PA1 [39,40] lacking cellulose synthesis genes to optimize liquid growth measurements [41]. Thus, the genotype referred to herein as “wild-type” (CM2730) is more accurately Δ*celABC*.

*E*. *coli* strains used in the physiological studies were derivatives of BW23474 [42], while those used for cloning and protein overexpression were derivatives of *E*. *coli* NEB 10-beta and BL21 (DE3) (Stratagene), respectively.

All strains used in this study are described in Table 1 and S1 Table.

**Table 1:** Evolved beneficial alleles.

PRIMARY MUTATIONS			
Allele	Position	Mutation	Annotation
*Mext_4158* Protein of unknown function DUF336 (EfgA)			
*efgA*^*evo1*^	4627319	C→T	T60I
*efgA*^*evo2*^	4627352	G→A	G49D
*efgA*^*evo3*^	4627304	G→A	G65D
*efgA*^*evo4*^	4627151	Δ11 bp	frameshift
*efgA*^*evo5*^	4627109	Δ63	deletion
*efgA*^*evo6*^	4627487	C→T	T4I
*efgA*^*evo7*^	4627177	G→A	M107I
*efgA*^*evo8*^	4627428	A→C	T24P
*efgA*^*evo9*^	4627492	C→G	H2Q
*efgA*^*evo10*^	4627157	G→A	S114N
*efgA*^*evo11*^	4627226	T→A	L91H
*efgA*^*evo12*^	4627275	T→C	S75P
*efgA*^*evo13*^	4627170	G→C	A110P
*efgA*^*evo14*^	4627223	Δ1 bp	frameshift
*efgA*^*evo15*^	4627223	Δ1 bp	frameshift
*efgA*^*evo16*^	4627173	G→T	G109C
*efgA*^*evo17*^	4627305	G→A	G65S
***Mext_1636*** **Peptide deformylase (PDF)**			
*def*^*evo1*^	1824018	G→A	G143S
*def*^*evo2*^	1824284	T→G	V54G
***Mext_0925*** **MarR family regulator**			
*Mext_0925*^*evo1*^	1001161	C→G	H119D
*Mext_0925*^*evo2*^	1001376	Δ1 bp	frameshift
***Mext_4194*** **ABC transporter-like protein**			
*potG*^*evo1*^	4664528	G→A	R115Q
***Mext_4478/Mext_4479*** **Hypothetical protein/ribosomal L11 methyltransferase**			
*prmA*^*evo1*^	4996712	G→C	−541/−77
SECONDARY MUTATIONS			
***Mext_0399*** **Heat-inducible transcription repressor HrcA**			
*hrcA*^*evo1*^	443917	Δ1bp	frameshift
***Mext_0606*** **Adenylate cyclase**			
*efgB*^*evo1*^	666124	C→T	R332C
*efgB*^*evo2*^	666131	G→A	E313K
***Mext_1058*** **Molybdenum cofactor cytidylyltransferase**			
*Mext_1058*^*evo1*^	1156254	G→C	G91R
***Mext_2112*** **XRE family transcriptional regulator/shikimate kinase**			
*Mext_2112*^*evo1*^	2369958	T→C	L105P
***Mext_2690*** **P-type Cu+ transporter**			
*Mext_2690*^*evo1*^	3008713	G→C	G610R
***Mext_3596*** **Serine protease Do/acid stress chaperone HdeA**			
*mntR*^*evo1*^	3975117	A→G	T108A
***Mext_3827/Mext_3828*** **Serine protease Do/acid stress chaperone HdeA**			
*Mext_3828*^*evo1*^	4252124	T→C	+371/−293
***Mext_4337/Mext_4338*** **Cytochrome o ubiquinol oxidase/transmembrane transporter protein**			
*cyoA*^*evo1*^	4822969	Δ37 bp	−54/+105
***Mext_4456*** **Beta-lactamase domain protein**			
*Mext_4456*^*evo1*^	4963821	T→C	Y265H

Growth experiments for *M*. *extorquens* were performed in a modified Hypho medium [43] or *Methylobacterium* PIPES (MP) medium [41] with 3.5 mM succinate, 15 mM methanol, or 2, 4, 5, 6, 8, 10 mM formaldehyde as a sole carbon source. Growth and formaldehyde phenotypes were comparable between the two media. For growth on solid medium, Bacto Agar (15 g/L, BD Diagnostics Franklin Lakes, NJ, New Jersey, USA) was added, and the concentrations of succinate or methanol were increased to 15 or 125 mM, respectively, or BD Difco nutrient agar was used.

Formaldehyde stock solutions (1 M) were prepared by boiling 0.3 g paraformaldehyde and 10 mL of 0.05 N NaOH in a sealed tube for 20 m; stocks were kept at room temperature and made fresh weekly (growth experiments) or daily (*in vitro* binding experiments). When present in the media, compounds were at the following final concentrations: kanamycin (50 μg/mL), tetracycline (12.5 μg/mL), trimethoprim (10 μg/mL), streptomycin (100 μg/mL), sucrose (50 g/L), and cumate (30 μg/mL). Stock solutions of glyoxal, acetaldehyde, butyraldehyde, glutaraldehyde, and propionaldehyde were prepared in water or ethanol.

Growth experiments for *E*. *coli* were performed in MOPS medium [44] with 2 mM glucose, 0.7 to 1.1 mM formaldehyde, and 0.5 mM L-rhamnose for induction.

Chemicals were purchased from Sigma Aldrich, St. Louis, MO, USA, VWR, Radnor, PA, USA, or ThermoFisher Scientific, Waltham, MA, USA.

### Growth analyses

Starter cultures of *M*. *extorquens* (2 mL) were grown in biological triplicate by inoculating media with individual colonies. Cultures were grown in Hypho or MP liquid medium with shaking (250 rpm on platform shaker or 70 rpm in a New Brunswick TC-7 culture roller drum) during incubation at 30°C. Formaldehyde resistance phenotypes are comparable between Hypho and MP medium. Early stationary-phase cultures (24 h for succinate, 36 h for methanol) were then subcultured (1/64) into relevant media for growth measurements. Cell density was determined by monitoring absorbance with a Spectronic 200 (Thermo Fisher, Waltham, MA, USA) or a SmartSpec Plus (Bio-Rad, Hercules, CA) at 600 nm. To determine cell viability (CFU/mL), a 100-μL aliquot of culture was used to harvest cells by centrifugation. The supernatant was discarded, and the cell pellet was resuspended into MP medium (no carbon). Cell suspensions were then serially diluted (1/10 dilutions, 200 μL total volume) in 96-well polystyrene plates with MP medium (no carbon), and 10 μL aliquots of each dilution were spotted to MP medium plates (15 mM succinate) in technical triplicate. Plates were inverted and incubated at 30°C until colony formation was apparent (4 to 6 d), at which point colonies were counted. Technical triplicates were averaged for each biological replicates, and biological replicates were averaged.

Starter cultures for growth analyses of *E*. *coli* were initiated from freezer stocks of WM8637 or WM8653 into 5 mL tubes at 37°C shaken at 250 rpm. After growth to stationary phase overnight, these were subcultured (1/500) into MOPS medium with 2 mM glucose. After overnight growth, both cultures were diluted to an OD_600_ = 0.02 into MOPS with 2 mM glucose with or without 0.5 mM rhamnose. A volume of 640 μL was pipetted into Costar 3548 48-well plates (Corning, Corning, NY, USA) and grown without lids at 37°C in Synergy H1 plate readers (BioTek, Winooski, VT, USA) with double-orbital shaking at 425 rpm and a 3-mm orbit and readings taken every 15 min (OD_600_ with 100 ms delay and 8 measurements per data point). After 2.33 h to establish exponential growth, the dispenser of the Synergy H1 was used to automatically deliver formaldehyde at 225 μL/s from a 32-mM stock made in H_2_O (volume added ranged from 14 to 22 μL) straight into the wells to final concentrations of 0.7, 0.9, or 1.1 mM. Data were analyzed by first subtracting the average of the blank wells used at the corners of the plate. The data shown are from non-edge wells on the plate; consistent trends with shifted times of recovery were found for the daily duplicates in the edge wells. The entire experiment was repeated on three additional days (each with duplicates) from separate starter cultures.

### Genomic context of *efgA* and *efgB* in other organisms

To examine synteny of *efgA* and *efgB*, the genomic context of each gene was examined in organisms with closely related homologs using GeneHood (https://gitlab.com/genehood/genehood-cli) and the MiST3 (Microbial Signal Transduction Database) [45]. Figures were generated using the GeneHood software.

### Experimental evolution

From individual colonies, three independent cultures of CM2730 were grown in 10 mL liquid Hypho medium in batch culture in 50 mL flasks (sealed) supplemented with 15 mM methanol. Upon reaching stationary phase, a 156-μL aliquot (1/32 inoculum) was transferred to fresh medium containing decreased methanol and increased formaldehyde concentrations (S1 Fig). For the first 60 generations, the concentration of formaldehyde increased with each transfer. For the next 90 generations, the concentration of formaldehyde was kept constant (20 mM). Final populations (150 generations) were plated to a series of solid media containing: (i) nutrient agar, 15 mM methanol, 3.5 mM succinate, 15 mM formate, 5 mM formaldehyde; (ii) Hypho, 4 mM succinate; (iii) Hypho, 100 mM methanol; and (iv) Hypho, 30 mM formate. Colonies that arose were streaked for isolation on the same respective medium and were further characterized.

A second round of experimental evolution used 25 individual colonies and only involved 4 transfers after an initial round of growth at 15 mM methanol: (i) 10 mM methanol, 1 mM formaldehyde; (ii) 5 mM methanol, 2.5 mM formaldehyde; (iii) 5 mM methanol, 5 mM formaldehyde; and (iv) 5 mM formaldehyde.

### Sequence acquisition and phylogenetics

#### Maximum likelihood phylogenetic tree of EfgA

The amino acid sequence of EfgA was used as a query with PSI-BLAST [46] using an 1E^−10^ cutoff in RefSeq [47]. From each of the 5,172 matches, the DUF336 sequence was extracted for analysis; sequences >30% identity were analyzed with CD-HIT [48], allowing us to eliminate all sequences with 90% or greater identity. The average amino acid size was then calculated for the database, and any sequence longer than 1σ was removed prior to alignment. The formaldehyde-bound crystal structure of EfgA, 6C0Z, was used as a query for homologous structures, which were then added into the sequence database manually. The 5,172 sequences were then aligned with MUSCLE [49] using default parameters. The alignment file was then analyzed with FastTree 2.1 [50] with the LG + CAT [51], WAG + CAT [52], and JTT + CAT [53] models with and without gamma distribution. The LG + CAT model generated the best LogLk (−584712.707) and the fewest bad splits (15/5,168). The phylogenetic reconstruction was then analyzed and annotated with iTOL [54] with marker positions for known structures added to the tree. The final phylogenetic data are available at TreeBASE (http://purl.org/phylo/treebase/phylows/study/TB2:S27073).

#### Maximum likelihood phylogenetic tree of EfgB

The nucleotide sequence of EfgB was used as a query with BLASTN [46] using a filter to eliminate all sequences with 90% or greater identity; the top 100 matches (all >65% identity) were used to assess phylogeny. The evolutionary history was inferred by using the maximum likelihood method and the general time reversible model [55]. The bootstrap consensus tree was inferred from 500 replicates [56]. The percentage of replicate trees in which the associated taxa clustered together in the bootstrap test (500 replicates) are shown next to the branches [54]. Initial tree(s) for the heuristic search were obtained automatically by applying Neighbor-Join and BioNJ algorithms to a matrix of pairwise distances estimated using the Maximum Composite Likelihood (MCL) approach, and then selecting the topology with superior log likelihood value. This analysis involved 97 nucleotide sequences. There were a total of 1,578 positions in the final dataset. Evolutionary analyses were conducted in MEGA X [57]. The final phylogenetic data are available at TreeBASE (http://purl.org/phylo/treebase/phylows/study/TB2:S27073).

### Genetic approaches in *M*. *extorquens* and *E*. *coli*

Allelic exchange was used to introduce changes into relevant genetic loci as previously described [58]. Due to challenges with identifying a tetracycline concentration that would reliably differentiate between strains with and without the resistance marker in *M*. *extorquens* PA1, we constructed a kanamycin-resistant version of the allelic exchange vector. A 2-kb region of pCM433 [58] encoding *cat* and most of *tet* was excised using *Eco*RV. The remaining vector backbone, with the exception of a 0.9-kb region containing *bla* was PCR amplified and joined via Gibson assembly (HiFi DNA Assembly, New England Biolabs, Ipswich, MA, USA) with a 1.3-kb PCR product containing *kan* PCR amplified from pCM66 [59] to generate pPS04 (S2 Fig). The complete sequence of pPS04 is available in GenBank (Submission 2392028, pending) and has been deposited at Addgene (169472).

Deletions of *efgA*, eliminated 404 bp from the ORF of *Mext_4158* (21-424/435); deletions of *efgB*, *fmt*, and *fmt-def* eliminated the entire coding region(s) of *Mext_0606* (1401 bp), *Mext_1635* (930 bp), and *Mext_1635-Mext_1636* (1456 bp), respectively.

Inducible expression vectors derived from pLC290 were used to express *efgA* from *M*. *extorquens* PA1 and *Mfla_1444* from *Methylobacillus flagellatus* KT in *M*. *extorquens* (pDN147 and pDN162, respectively). Vectors include 30 bp upstream sequence of each gene including respective native ribosomal binding sites.

The WM8655 strain was generated to express *efgA* from *M*. *extorquens* PA1 from the rhamnose-inducible P_*rhaS*_ promoter in *E*. *coli*. The *efgA* coding sequence plus 30 bp at the 5′ end was amplified using primers with 30 nt overlaps to permit Gibson assembly (HiFi DNA Assembly, New England Biolabs) into pAH120 [42] that had been digested with *Xba*I and *Nde*I, generating pDN380. An empty control strain, WM8637, was first generated by electroporation of pINT-ts into BW23474 [42]. pDN380 was introduced via electroporation into WM8637 to generate WM8655. Constructs were confirmed by analytical PCR and sequencing of the *efgA* locus.

All vectors were designed using SnapGene software. The Gibson assembly kit from New England Biolabs was used to construct vectors from restriction enzyme-digested, linearized vector backbone and PCR-generated inserts. For *E*. *coli*, transformations were performed using standard (WM8637 and WM8655) or manufacturer’s (BL21 (DE3)) protocols for chemical transformation. For complementation of *M*. *extorquens*, triparental conjugations were performed using pRK2073 [60].

### Formaldehyde quantification

#### In stocks and media

Formaldehyde concentrations in the culture media were measured as previously described [61]. Supernatant from a 100-μL aliquot of culture was isolated by centrifugation (14,000 × *g*). In technical triplicate, 10 μL of the supernatant or 100 μL of 0.1X supernatant (diluted with MP medium, no carbon) was combined with 190 or 100 μL Nash reagent B (2 M ammonium acetate, 50 mM glacial acetic acid, 20 mM acetylacetone), respectively, in 96-well polystyrene plates. Reaction plates were incubated (60°C, 10 min) and cooled to room temp (5 min), and absorbance was read at 432 nm on a Wallac 1420 VICTOR Multilabel reader (Perkin Elmer, Waltham, MA, USA). Formaldehyde standards were prepared daily from 1 M formaldehyde stock solutions, and a standard curve was alongside all sample measurements.

#### Internal formaldehyde

*M*. *extorquens* cells from 50 mL of culture were harvested by centrifugation (4,000 RPM for 10 m at 4°C) and resuspended in 1.5 mL of 25 mM Tris-HCl (pH 8) with 150 mM NaCl. Cell suspensions were lysed using a Bead Rupter 24 (Omni International, Kennesaw, GA, USA) set to 6 m/s for 90 s. Lysates were centrifuged (12,000 RPM for 10 m at 4°C), and the supernatant was transferred to a sterile 1.5-mL tube. The concentration of formaldehyde was determined using a previously described Purpald assay [62]. In short, 100 μL of lysate was mixed with 100 μL of Purpald (34 mM, dissolved in 2 M NaOH) in a 96-well polystyrene plate. Reactions were incubated (room temperature, 20 m), and then 100 μL of NaIO_4_ (33 mM, dissolved in 0.2 M NaOH) was added to each well. After bubbling subsided (approximately 20 m), the absorbance was measured at 550 nm on a SpectraMax i3x Multi-Mode Plate Reader (Molecular Devices, San Jose, CA, USA). Formaldehyde standards were prepared daily from a 1M stock solution, and a standard curve was prepared alongside all sample measurements. Intracellular formaldehyde concentrations were calculated by normalizing concentrations in cell lysates to total cell volume. Total cell volume was calculated based on total cell number, determined from CFU/mL as a function of cell density (Absorbance 600 nm) and average cell volume (2.63 fL). Intracellular formaldehyde concentration = formaldehyde concentration of lysate × 1.5 mL extraction volume / (CFU/mL of lysate [calculated from Abs_600_] × 50 mL lysate total volume × 2.63 fL/CFU).

### Crystallization and structure of *M*. *extorquens* EfgA in complex with formaldehyde

#### Expression and purification of EfgA from *M*. *extorquens*

The gene encoding EfgA (including the thrombin cleavable 6XHis tag) was transformed into *E*. *coli* strain BL21 (DE3) (Stratagene, La Jolla, CA, USA) for overexpression from pDN79. The protein was overexpressed by growing cells in Luria Bertani broth (LB) medium to an A_600_ of 0.6 at 37°C and subsequent induction with 0.5 mM isopropyl β-D-1-thiogalactopyranoside (IPTG) for 14 h at 16°C. The cell pellet was collected by centrifugation and resuspended in buffer A (50 mM Tris pH (7.5), 1 M NaCl, 20 mM imidazole, 0.3 mM DTT, 0.2 mM PMSF, and Complete protease inhibitor cocktail tablet, EDTA-free (Roche Diagnostics Corp, Indianapolis, Indiana, US)). Lysis was performed by sonication. The lysate was then centrifuged for 60 m at 24,000 rpm at 4°C. The supernatant was then applied onto a Hi Trap affinity (5 ml) (Ni^2+^) column (GE Healthcare Life Sciences, Marlborough, MA, USA) The column was washed with 10 column volumes of buffer A, and the protein was then eluted with increasing concentrations of imidazole from 20 to 500 mM. The fractions containing the protein of interest were pooled, dialyzed against 50 mM Tris (pH 7.5), 0.3 mM DTT, 0.5 mM EDTA, and purified over a Q-XL Sepharose column (GE Healthcare) using a 0.1- to 1-M NaCl gradient. The fractions containing EfgA were pooled and concentrated in an Amicon centrifugal filter concentrator with a 10-kDa cutoff membrane (Millipore, Burlington, MA, USA). The concentrated protein was then further purified by size-exclusion chromatography using a Superdex-200 column (GE Healthcare, HiLoad 16/60) equilibrated with buffer (50 mM Tris (pH 8.0), 250 mM NaCl). Again, the fractions containing the protein were pooled and concentrated with an Amicon centrifugal filter concentrator with a 10-kDa cutoff membrane. The purity of the protein was analyzed with 15% sodium dodecyl sulfate polyacrylamide gel electrophoresis (SDS–PAGE) by using ImageJ software and was determined to be greater than 95%.

#### Crystallization and structure

Crystals of *M*. *extorquens* EfgA were obtained by sparse matrix screening at 15 mg/mL at 4°C and 10°C. Preliminary results were followed by optimization of the successful condition manually using the sitting drop vapor diffusion method. The best quality crystals were grown in 0.2 M potassium fluoride, 2.2 M ammonium sulfate ((NH_4_)_2_SO_4_) at 10°C. The EfgA-formaldehyde crystals suitable for data collection were grown at 10°C in 0.2 M KNO_3_, 2.2 M (NH_4_)_2_SO_4_, 16.6 mM formaldehyde using the hanging drop vapor diffusion method. Diffraction data sets for *M*. *extorquens* EfgA were collected at 100 K at 1.65 Å resolution at the Argonne National Laboratory’s Advanced Photon Source (ANL APS) beamline 21-ID-G on a MarMosaic 300 CCD detector. Diffraction data for EfgA in complex with formaldehyde were collected at 100 K at 1.85 Å resolution at ANL APS beamline 21-ID-D on a MarMosaic 300 CCD detector. X-ray diffraction data were processed using HKL2000 [63]. The crystal of apo-protein belonged to the space group P22121 with the unit cell parameters a = 71.82, b = 72, c = 105.18, α = β = γ = 90°. *M*. *extorquens* EfgA in complex with formaldehyde crystallized in space group P21221 with the unit cell parameters a = 72.06, b = 72.06, c = 104.56, α = β = γ = 90°. The 3-dimensional structures were determined by molecular replacement using *Klebsiella pneumoniae* protein OrfY (Pfam DUF336) domain as the search model in Phaser-MR [64]. The molecular replacement for EfgA-formaldehyde complex was further confirmed by the initial (2Fo- Fc) map generated using Coot [65] that clearly indicated electron density for the formaldehyde that was not included in the original search model. The structure was refined using the Phenix suite [66] and Coot [65]. Ramachandran plots and root-mean-square deviations (rmsd) from ideality for bond angles and lengths were determined using a structure validation program, MolProbity [67]. A summary of data collection and refinement statistics are listed in Table 2.

**Table 2:** Data collection and refinement statistics.

Data set	EfgA^WT^	EfgA^WT^/soaking with Formaldehyde
Data collection		
Wavelength (Å)	1.000	0.97934
Resolution (Å)^a^	42.5–1.6 (1.7–1.6)	42.3–1.8 (1.89–1.83)
Space group	P22121	P21221
Cell dimensions a, b, c; (Å) α, β, ɣ; (°)	71.82, 72.0, 105.18 90, 90, 90	72.06, 72.06, 104.55 90, 90, 90
Molecules per a.u.	4	4
Unique reflections^a^	62821 (6085)	47983 (4714)
Average redundancy^a^	12.9 (12.5)	11.6 (11.4)
Completeness (%)^a^	94.7 (95.5)	98.37 (97.32)
R_merge_ (%)^a^^,^^b^	13.6 (92.2)	11.9 (54.5)
Output <I/sigI>^b^	19.8 (1.8)	26.1 (4.7)
Refinement		
R_work_ (%)^c^	22.15 (28.15)	21.59 (23.06)
R_free_ (%)^d^	24.53 (29.94)	25.46 (26.41)
r.m.s.d.^e^ from ideality Bonds (Å) Angles (°)	0.003 0.571	0.009 0.94
Average B-factor (Å^2^)	20.82	25.93
Ramachandran^f^ Favored (%) Allowed (%) Outliers (%)	96.30 3.70 0	98.33 1.67 0
PDB ID	6BWS	6COZ

^a^Values for the last shell are in parenthesis.

^b^R_merge_ = Σ│I—<I>│/ Σ I, where I is measured intensity for reflections with indices of hkl.

^c^R_work_ = Σ│F_o_- F_c_│/ Σ│F_o_│ for all data with F_o_ > 2 σ (F_o_) excluding data to calculate R_free_.

^d^R_free_ = Σ│F_o_- F_c_│/ Σ│F_o_│ for all data with F_o_ > 2 σ (F_o_) excluded from refinement.

^e^Root mean square deviation

^f^Calculated by using MolProbity [67].

### In silico predictions of formaldehyde binding site and folding and binding stabilities of EfgA variants

To predict the location of the formaldehyde binding pocket on the EfgA tetramer, and to estimate folding and binding stabilities of EfgA variants, classical molecular dynamics (MD) simulations were carried out. These simulations used the apo EfgA tetramer X-ray crystal structure (Protein Data Bank (PDB) ID: 6BWS) and EfgA monomer (chain A of EfgA tetramer) and were performed using GROMACS v2018 [68]. The AutoDock Vina program [69] was then used to dock formaldehyde to snapshots of the EfgA tetramer for 100 snapshots extracted from the MD simulations to determine the pockets most heavily populated with high scoring poses. EfgA monomer and tetramer snapshots were analyzed using FoldX software (MD+FoldX approach) to estimate folding and binding stabilities of EfgA variants.

#### Molecular dynamics simulation of EfgA monomer and tetramer

Both EfgA tetramer and EfgA monomer structures were subjected to atomistic MD simulations using the same protocol. MD simulations were performed using AMBER99SB*-ILDN [70] forcefield. The EfgA structure was placed in a cubic box of TIP3P water, and the net charge was neutralized by adding Na^+^ and Cl^−^ ions at a concentration of 0.15 M. Protonation states for all ionizable residues were automatically assigned for neutral pH. The system was then minimized using the steepest descent algorithm for 10,000 steps. The subsequent equilibration process was to perform 1 ns simulation with the positions of all heavy atoms in the complex harmonically restrained to allow equilibration of the water molecules around the proteins, followed by another 1 ns simulation with no restraints. During equilibration, the temperature and the pressure of the system was set to 300°K and 1 atm, respectively, using the Berendsen algorithm [71]. Production simulations were then carried out for 100 ns with pressure maintained using Parrinello-Rahman barostat [72], and temperature was controlled using the v-rescale thermostat [73]. Particle mesh Ewald [74] was used to treat electrostatics with a real-space cutoff of 1.2 nm. Van der Waals interactions were cut off at 1.2 nm with the Potential-shift-Verlet method for smoothing interactions. The LINCS algorithm [75] was applied to constrain all bonds to their ideal lengths, and timestep of 2 fs was used. During the 100 ns production simulation snapshots were saved every 1 ns giving 100 snapshots of EfgA tetramer and EfgA monomer to be used for docking calculations and FoldX analysis.

#### Docking of formaldehyde to EfgA tetramer

Each of the 100 EfgA tetramer snapshots obtained during MD simulations was used to dock formaldehyde with the AutoDock Vina software [69]. Generally, docking programs allow the ligand to be completely flexible during the conformational search with only a few restricted side chains on the protein assigned as flexible. Use of 100 snapshots from MD simulation allows us to at least partly overcome this limitation.

The 3-D coordinates of formaldehyde were obtained from PubChem (https://pubchem.ncbi.nlm.nih.gov) and assigned Gasteiger partial charges using Autodock Tools (http://mgltools.scripps.edu/). Ligand docking was then carried out by creating a grid box of size 80 Å × 60 Å × 60 Å, centered on the geometric center of the EfgA tetramer, with a grid spacing of 1 Å. All regions of the tetramer protein complex were included in the search for the most favorable interactions of the ligand. The input exhaustiveness parameter for the docking was set to 400. The number of top docking orientations with high docking scores was fixed to 20. This docking protocol was applied to all 100 snapshots and the X-ray crystal structure of the EfgA tetramer, yielded 2020 (101 snapshots × 20 top docking conformations) conformations of formaldehyde bound to the EfgA tetramer. Highly populated docking clusters were then identified using VolMap plugin built in VMD software [76].

#### Predicting folding and binding stabilities of EfgA variants

A mutation of EfgA can affect the folding of a monomer and/or the formation of the tetramer. In order to determine how amino acid mutations, including the known variants, alter stabilities (ΔΔG values) for EfgA folding and formation of a tetramer, we calculated ΔΔG values of folding and binding using our previously successful MD+FoldX approach [77–79]. This involves analyzing MD snapshots with FoldX software [80]. MD snapshots of the EfgA monomer and tetramer were analyzed using the same protocol reported in our previous study [79]. Briefly, each snapshot was subjected to the RepairPDB command 6 times in succession to minimize and obtain convergence of the potential energy. For each snapshot, all possible 19 single mutations in the monomer/tetramer at each amino acid site were then generated using BuildModel command. Lastly, the folding stability of the EfgA monomer due to each mutation was estimated using Stability command, and the binding stability of the EfgA tetramer was estimated using AnalyseComplex command. For each mutation, we then estimated ΔΔG of folding and binding by averaging the FoldX results across all individual snapshot estimates. This process led to a total of 2,546 (134 EfgA residues ×19 possible mutations at each site) ΔΔG values for both folding and binding.

### Comparison of structural homologs

Structures for EfgA homologs (OrfY– 2A2L, HbpS– 3FPV, Ybr137w – 4CLC, DESPIG_02683 – 4NKP, PduOC– 5CX7, and EfgA– 6BWS and 6C0Z) were acquired from PDB (https://www.rcsb.org/) and aligned with PyMol v2.3 [81].

### EfgA:ligand binding assays

#### Expression and purification of EfgA

Recombinant EfgA containing a C-terminal 6X-His tag was expressed in *E*. *coli* BL21(DE3) housing pET28 vectors (Novagen, Madison, WI, USA). Single colonies from LB supplemented with 50 μg/mL kanamycin were used to generate 25 mL overnight cultures grown at 37°C with continuous shaking at 250 RPM. Cultures were diluted to an OD_600_ = 0.05 in a 1 L flask containing 800 mL of LB kanamycin then placed at 37°C. When cultures reached OD_600_ approximately 0.52 to 0.60, they were induced with 1 mM IPTG and grown for 4 h at 37°C. Cells were then harvested by centrifugation at 10,000 × *g* for 20 m at 4°C. Harvested cells were washed with Buffer A (50 mM Na_2_HPO_4_, 300 mM NaCl, 35 mM imidazole at pH 8.0), then harvested again and resuspended in 15 mL Buffer A. Cells were lysed via French Press (Thermo Fisher Scientific) in a prechilled cell at 20,000 psi. Cell lysate was cleared by centrifugation at 50,000 × *g* for 2 h at 4°C followed by filtration through 0.22 μm PTFE filters and stored at −80°C prior to purification.

Protein lysates were thawed at 4°C and centrifuged briefly to ensure no precipitants were present prior to column chromatography. An NGC FPLC (Bio-Rad) was used to purify the C-terminal 6X-His EfgA with 1 mL Ni-NTA columns (Bio-Rad). Columns were equilibrated with 10 mL of Buffer A at 1 mL/min prior to loading lysates. Lysates were loaded at 0.35 mL/min followed by 10 mL of Buffer A at 1 mL/min. An isocratic phase was generated by passing 10 mL of Buffer B (50 mM Na_2_HPO_4_, 300 mM NaCl, 500 mM imidazole at pH 8.0) gradually through the column in reverse phase at 0.5 mL/min with samples being collected every 0.5 mL. Degassed H_2_O (10 mL) was run at 1.0 mL/min until conductivity and absorbance (280 nm) were zero. Samples with high 280 nm values were collected during the isocratic phase of Buffer B.

Fractions from FPLC purification were treated with Laemmli buffer [82] with 10 mM 1,4-dithiothreitol (DTT) and heated for 5 m at 85°C then analyzed on a 4% to 15% discontinuous SDS–PAGE gel with a 6% stacking gel run at ambient temperature at a constant 100 V. Samples that showed high purity were pooled and stored with 9% glycerol (v/v) and then quantitated with a Bradford assay [83] prior to storage at −80°C.

#### Microscale isothermal calorimetry (mITC)

Binding of formaldehyde and EfgA was measured via mITC using an Affinity ITC–LV (TA Instruments–Waters LLC, New Castle, DE, USA). An isothermal buffer for experimental titrations of EfgA was designed to minimize binding between ligands and buffer (50 mM Na_2_HPO_4_, 300 mM NaCl (pH 8.0)). Pooled samples of EfgA or EfgA variants were buffer exchanged 1:128 fold with mITC buffer using 3K MWCO Amicon cellulose acetate filters at 4,000 × *g* for 30 m in a swinging bucket rotor. Protein samples were quantitated via Bradford assay and normalized to 2 mg/mL using mITC buffer. Initially, protein:ligand concentrations were used in varying ratios to determine working assay parameters. Proteins were then diluted in mITC buffer to a final concentration of 50 μM. Formaldehyde (1 M) was prepared from paraformaldehyde in milliQ-H_2_O and used within 24 h. Formaldehyde stock was serially diluted to 25 mM in mITC buffer. Methanol, formate, and acetaldehyde were all prepared in the same way as formaldehyde to minimize difference between ligand preparation. Prior to use, protein samples and buffer were degassed for 10 m at 650 mm Hg; ligands were degassed for only 5 m to minimize vapor loss.

All experimental runs were performed with 400 μL mITC buffer in the reference and 400 μL sample cells. Between runs, the sample cell and titration syringe were washed 10 times with degassed H_2_O and 10 times with degassed mITC buffer. The run protocol was 20 injections of 2 μL of 25 mM ligand (with the first being a 0.3-μL throw-away titration) every 200 s with a stir speed of 125 RPM, and 25°C. Prior to any run or data collection, a slope (μW/h) difference of 0.30 and standard deviation (μW) of 0.03 was required between reference and sample cell.

Isotherms of buffer:ligand were subtracted from protein:ligand data prior to calculation of binding energies. The blank μcal energy was subtracted from the total, which acted as the heat of dilution of ligand into protein.

#### Microscale thermophoresis (MST)

MST experiments were performed on a Monolith NT.115 system (Nanotemper Technologies, San Francisco, California, USA). A solution of unlabeled formaldehyde was serially diluted in reaction buffer (10 mM Na_2_HPO_4_, 1.8 mM KH_2_PO_4_, 2.7 mM KCl, 137 mM NaCl, 0.05% (v/v) Tween-20) to which an equal volume of Alexa-647 labeled EfgA was added to a final concentration of 20 nM. The samples were loaded into standard treated capillaries (Nanotemper) using 70% LED and 80% IR-laser power. Laser on and off times were set at 30 s and 5 s, respectively. The resulting K_d_ values are based on an average from three independent MST measurements. Temperature of MST experiments were 20°C and 30°C. Data analysis was performed using Nanotemper Analysis software, v.1.5.41.

The raw MST traces for each individual experiment were transformed and fit according to published methods by fitting the normalized fluorescence (F_norm_) to the Hill equation:

(F_norm_ = (F_norm,max_ − F_norm,min_) × [c_A_]^n^ / ([c_A_]^n^ + K_d_^n^), where F_norm,max_ = maximal normalized fluorescence, F_norm,min_ *=* minimal normalized fluorescence, [c_A_] = concentration of protein, K_d_ = dissociation constant, and n = hill coefficient.

### Formaldehyde tolerance distributions

Formaldehyde tolerance is defined as the maximum concentration of formaldehyde in which a given cell in a population can grow. To compare the distribution of formaldehyde tolerance phenotypes among individual cells in populations of wild-type and Δ*efgA* mutants, formaldehyde tolerance assay plates were prepared as follows: MP medium was prepared with agar, autoclaved, and cooled to 50°C; then, methanol (final concentration: 125 mM) and formaldehyde (to the desired final concentration) were rapidly mixed in, and the agar was poured into 100 mm petri dishes. The dish lids were immediately replaced, and plates were cooled on the benchtop. Plates were stored at 4°C and used within one week of pouring. Colony-forming units (CFUs) were plated and enumerated as described above.

Cell cultures were grown in MP-methanol medium until stationary phase. They were then subjected to serial 1:10 dilutions in MP to a final dilution of 10^−6^. From each of the seven dilutions, three replicates of 10 μL were pipetted onto each MP-methanol-formaldehyde plate to form spots (total: 21 spots per sample per plate type). The spots were allowed to dry briefly in a laminar flow hood, then lids were replaced and plates were stored in plastic bags and incubated at 30°C for 4 d before colonies were counted. For each replicate set of seven spots, the two highest-dilution spots with countable colonies were enumerated and summed, then multiplied by 1.1 times the lower of the two dilution factors to calculate the original number of CFUs in the sample. The frequency of tolerant cells at each formaldehyde concentration was then calculated by expressing the number of CFU at that concentration as a proportion of the CFU measured at 0 mM. For each sample, the mean and standard deviation of the three replicate spot series was calculated. To compare the shape of the curves, we measured the rate of the decline of tolerant cells relative to formaldehyde concentration (log_10_ cells / mM formaldehyde) by fitting a line to the points where frequencies are consistently statistically different (for WT, the last four non-zero values; for Δ*efgA*, the last four values).

This method has a limit of detection of 1.65 × 10^−7^ (an abundance of 34 CFU/mL is necessary to observe one cell per 30 μL plated, and the total cell population tested was 2 × 10^8^ CFU/mL; therefore, the least-abundant subpopulation that could be detected, disregarding the effects of Poisson distributions at lower λ, is one with an average frequency of 1.65 × 10^−7^ within the total population). Although this assay measures the growth of bacterial colonies and not directly that of individual cells, it has been demonstrated to correlate well with single-cell methods of measuring formaldehyde tolerance distribution [35].

### Competition assays

In biological triplicate, strains were acclimated to growth in Hypho supplemented with 15 mM methanol. In each competition, a test strain was mixed 1:1 (by volume) with a fluorescent reference strain expressing mCherry (CM3841, *efgA*^*evo1*^ *efgB*^*evo1*^ Δ*hpt*::*P*_*tacA*_*-mCherry*). This mixture was then used to subculture (1/64 inoculum) into 5 mL of identical fresh medium and grown as described above. The frequencies of fluorescent and nonfluorescent cells were quantified at the start (F0, t = 0) and end (F1, t = h when cells reached stationary phase) of the competition experiment using an LSRII flow cytometer (BD, Franklin Lakes, New Jersey). mCherry was excited at 561 nm and measured at 620/40 nm. For a given sample, at least 1,000 cells were counted. An identical set of competitions were set up with 5 mM formaldehyde in place of methanol as the sole carbon source in the growth medium for acclimation and subsequent competition assays. Malthusian fitness values (W) relative to the reference strain were calculated by a previously described equation assuming an average of 64-fold size expansion of mixed populations during competitive growth: W = log(F_1_ × 64 / F_0_) / log((1 − F_1_) × 64 / (1 − F_0_)); for a 32-fold size expansion in formaldehyde: W = log(F_1_ × 32 / F_0_) / log((1 − F_1_) × 32 / (1 − F_0_)) [43].

### In vivo translation assays

Succinate-growth stationary phase cultures of wild-type (CM2730) and the Δ*efgA* mutant (CM3745) were inoculated into MP medium with 15 mM succinate in biological triplicate and grown at 30°C with shaking. At early exponential phase (OD_600_ = 0.25), 1 mM [^13^CD_3_]-methionine (Met) was added to each culture and mixed to homogeneity. Each culture was divided into three aliquots and treated with (i) nothing; (ii) 5 mM formaldehyde; or (iii) 50 μg/mL kanamycin and immediately returned to the incubator. At 0, 20, 40, 60, 90, 180, and 360 min, the optical density was measured and cells from 1 mL of culture were harvested by centrifugation (top speed, 2 m) for [^13^CD_3_]-Met quantification by modification of a previously described method [84]. For time = 0 m, cells were harvested immediately post [^13^CD_3_]-Met addition, prior to formaldehyde/kanamycin treatment. Cells were washed with 1 mL of MP medium (no carbon) with 5 s vortex and again harvested by centrifugation. Cell pellets were resuspended in 200 μL of 6 M HCl. The suspension was transferred to a new Eppendorf tube, incubated (105°C, 18 h), and then dried (lid open, 95°C, approximately 20 h). Pellets were resuspended in dimethylformamide (DMF), and the suspension was transferred to a new Eppendorf tube. Amino acids were derivatized with the addition of *N*-*tert*butyldimethylsilyl-*N*-methyltrifluoroacetamide with 1% (wt/wt) *tert*butyldimethyl chlorosilane (TBDMSTFA), incubated (85°C, 1 h), and then transferred to GC/MS vials for analysis. GC parameters with minor modifications: pressure, 124.5 psi; total flow, 17.9 mL/min; column flow, 1.35 mL/min; column length, 29 m.

GC/MS analysis showed that the peak area ratio of major peaks 218 m/z (standard Met) and 222 m/z ([^13^CD_3_]-Met) was well correlated with the presence of each species in a mixture and was thus used to measure [^13^CD_3_]-Met incorporation in cells.

## Results

### Evolution of *Methylorubrum extorquens* on lethal concentrations of formaldehyde identifies two novel loci, *efgA* and *efgB*, with homologs that are stress response sensors

We evolved *M*. *extorquens* PA1 [40] for robust growth on formaldehyde in order to identify what loci might be involved in sensing and responding to formaldehyde toxicity. The first of these experiments involved using a steady transition from growth on 15 mM methanol to growth on 20 mM formaldehyde in the first 60 generations; this concentration was maintained to generation 150 (S2 Fig). Testing growth of individual isolates confirmed growth at these previously lethal concentrations and showed increased bacterial density with higher formaldehyde concentrations, presumably due to an increase in available carbon (S3 Fig).

Resequencing the genome of a representative isolate from each of the 3 populations identified nonsynonymous mutations in 2 genes of unknown function that were both mutated in each lineage, *Mext_4158* and *Mext_0606*. *Mext_4158* encodes a single domain, 144 amino acid protein with a domain of unknown function (DUF) 336. *Mext_0606* encodes a 466 amino acid protein that encodes a putative adenylate/guanylate cyclase. Neither gene resides in an apparent operon nor are they located near known methylotrophy genes (S4 Fig).

Further sequencing in 10 additional, randomly picked isolates from each population confirmed the prevalence of nonsynonymous mutations in *Mext_4158* and *Mext_0606* and identified mutations in a few other loci that did not occur in more than one population (Table 1, S1 Table). The repeated occurrence of mutations in *Mext_4158* and *Mext_0606* suggested that these 2 loci are of particular importance for **e**nhanced **f**ormaldehyde **g**rowth, thus we renamed them *efgA* and *efgB*, respectively. In a second round of evolution experiments to rapidly obtain growth in 5 mM formaldehyde, isolates from 18 of the 25 populations had mutations in *efgA* (two frameshifts, a 63-bp deletion, the remainder nonsynonymous mutations (Table 1, S1 Table)); no mutations were identified in *efgB*. Subsequent experimentation described below differentiated the respective roles of *efgA* and *efgB* in formaldehyde growth.

### Homologs of EfgA are specifically associated with methylotrophic lineages

**Fig 2:**
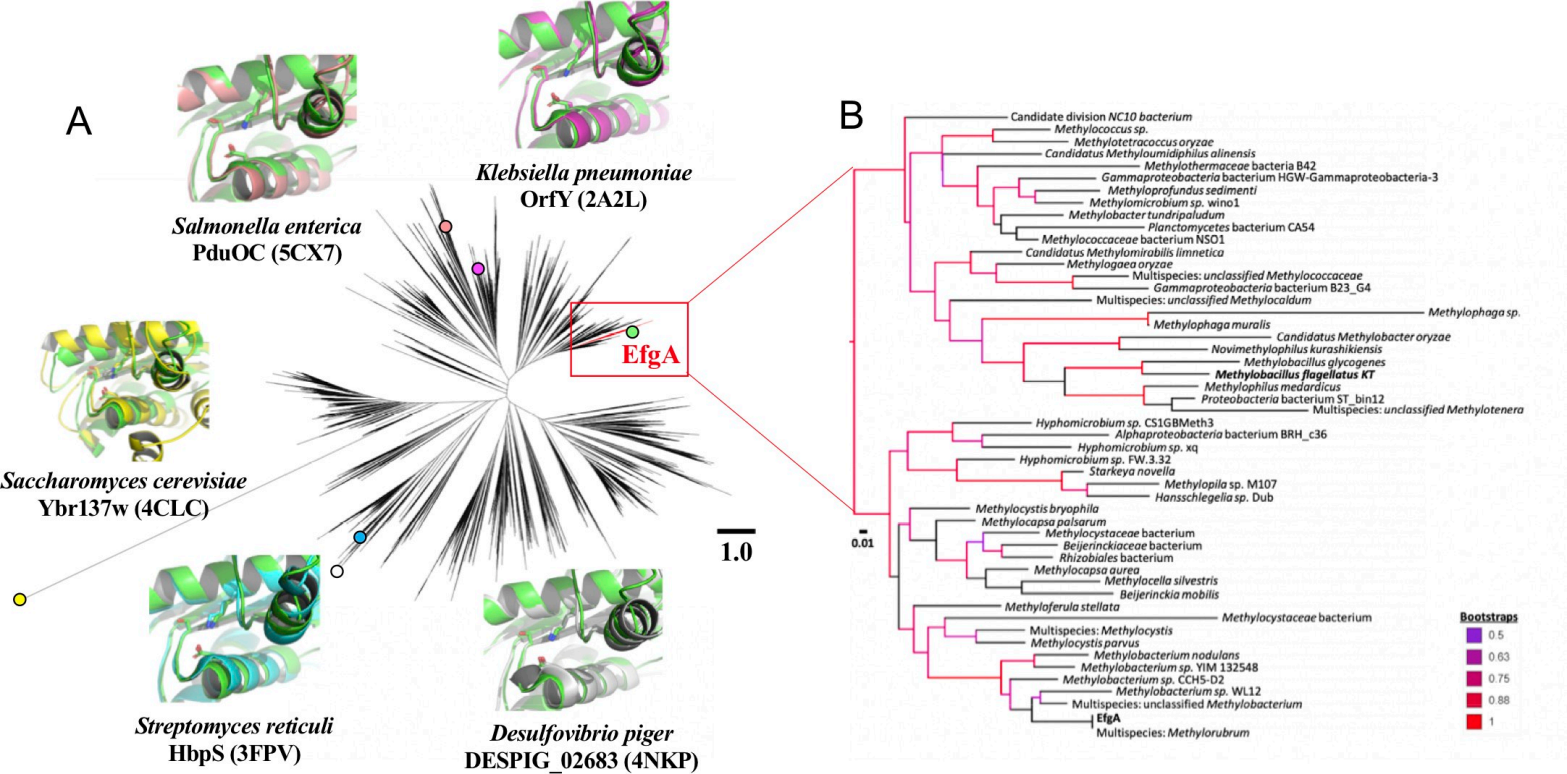
Phylogenetic analysis of the DUF336 superfamily indicates close homologs to EfgA are present in a broad array of methylotrophs. (A) The evolutionary relationship of the DUF336 region was compared to all current amino acid sequences and structures currently on NCBI via maximum likelihood. Solved structures of EfgA homologs are represented on the tree with their respective PDB accession codes and overlaid onto EfgA structure (green). The red colored sequences represent members of the EfgA clade. (B) Expanded view of the EfgA clade with bootstrap values above 50 highlighted by color according to the key. Bolded names indicate experimentally verified EfgA-like function. Scale bars for both (A) and (B) indicate substitutions per residue. The phylogenetic data are available at TreeBASE (http://purl.org/phylo/treebase/phylows/study/TB2:S27073).

Phylogenetic analysis demonstrated that DUF336 domains are encoded in all three domains of life, and the representatives with structures are broadly dispersed across the tree (Fig 2A). A few DUF336 family members are encoded in gene clusters of well-characterized metabolic pathways [85–89], and some of these DUF336 proteins are localized within bacterial microcompartments, proteinaceous organelles that can confine catabolic processes that involve the generation of toxic, and often volatile aldehydes [86,87,90]. The role of DUF336 domain in these contexts, however, has remained elusive. The only comprehensive studies on bacterial DUF336 function were with HbpS in the gram-positive bacterium *Streptomyces reticuli* [91–97]. HbpS localizes to the extracellular face of the cytoplasmic membrane where it senses environmental heme and, in turn, initiates a signaling cascade that mitigates oxidative stress. This raises the possibility that EfgA, despite low levels of sequence similarity to other DUF336 domains (23% identity to HpbS) may also play a role in sensing.

Phylogenetic analyses suggest that EfgA is linked to methylotrophy. Focusing upon close homologs of EfgA, we noted that these were found in a highly supported clade whose members are almost exclusively characterized methylotrophs (Fig 2B). These 51 sequences originate from a broad phylogenetic range including *alpha*-, *beta*-, and *gammaproteobacteria*, and NC10 (e.g., *Methylomirabilis oxyfera*) clades. However, we noted that some methylotrophic groups, such as those in the genus *Bacillus*, do not encode an EfgA homolog within this clade.

### Loss of EfgA function necessary and sufficient for growth on formaldehyde

**Fig 3:**
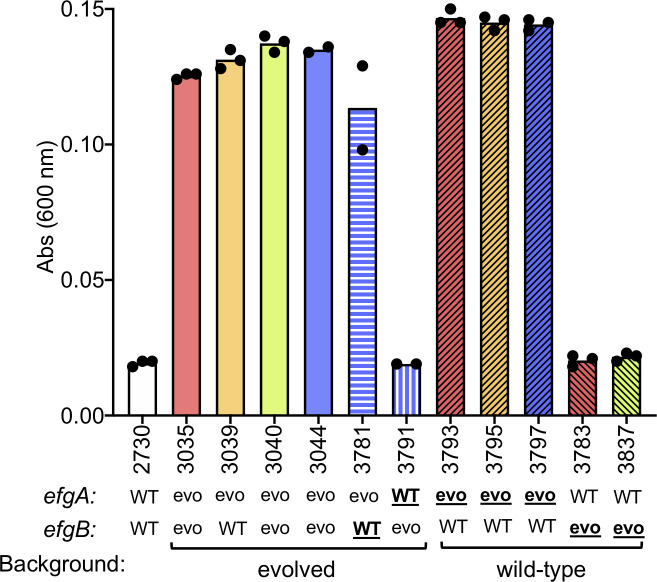
Evolved alleles of *efgA* are necessary and sufficient to confer growth on formaldehyde. Growth (24 h) of strains grown in liquid MP medium with 5 mM exogenous formaldehyde provided as a sole source of carbon and energy is shown. The wild type (CM2730) was grown alongside isolates evolved to grow on 20 mM formaldehyde: CM3035 (pink), CM3039 (orange), CM3040 (green), CM3044 (violet). Growth of genetic derivatives of CM3044 is also shown; in these strains (CM3781, CM3791), WT alleles of *efgB* or *efgA* were introduced into CM3044 to replace the evolved (“evo”) alleles. Lastly, growth of genetic derivatives of the wild-type strain is shown; these strains were made by introducing distinct *efgA*^*evo*^ alleles (CM3793, CM3795, and CM3797) or *efgB*^*evo*^ alleles (CM3783, CM3837) found in formaldehyde-evolved isolates into CM2730 to replace the native WT alleles. For constructed strains, colors of bars correlate to evolved isolate that alleles were originally isolated from and the alleles that were genetically introduced into a particular strain background are bolded. The original data shown in this and all other figures are available in Supporting information file S1 Data.

**Fig 4:**
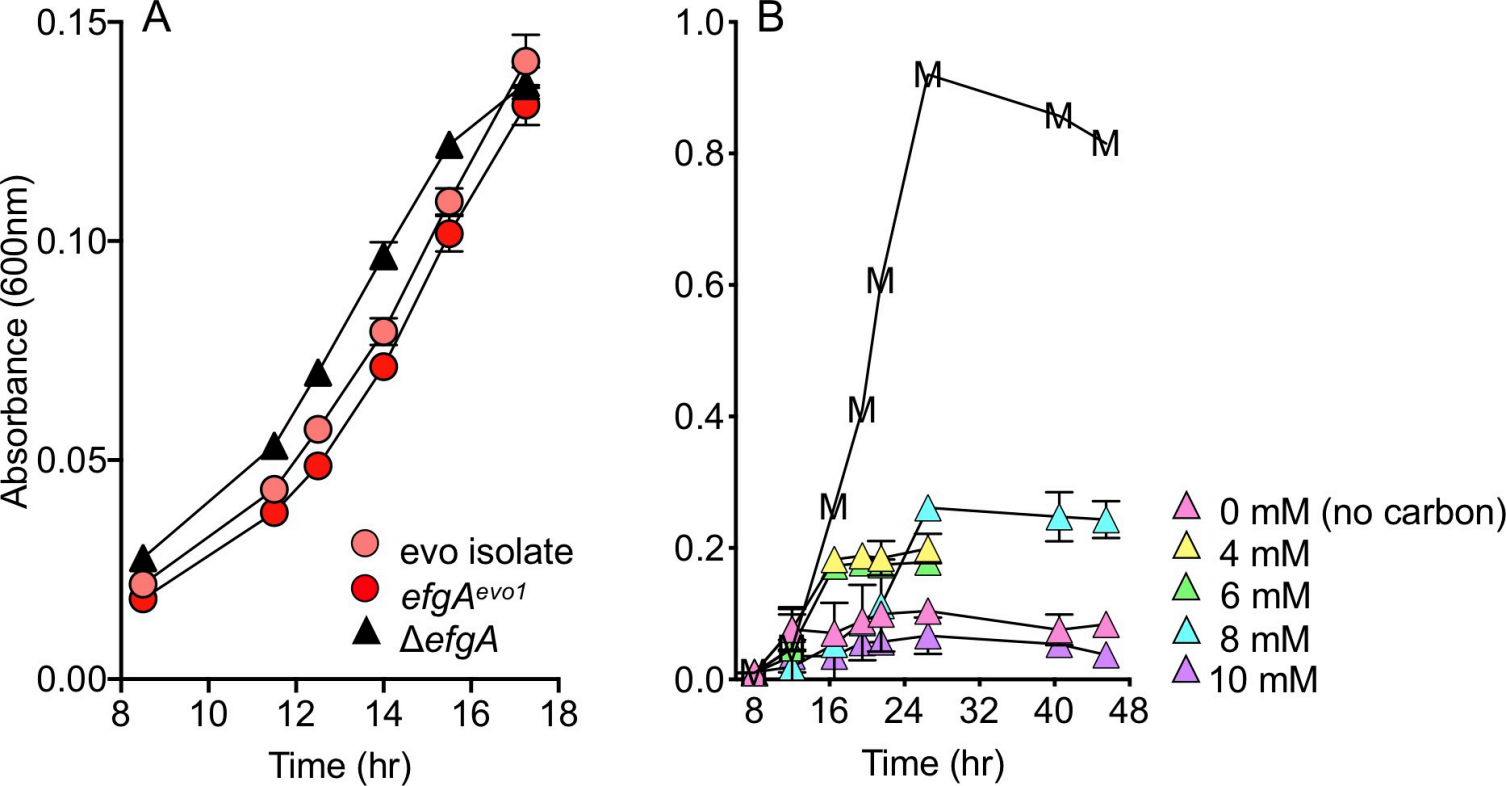
Evolved *efgA* allele or deletion of *efgA* recapitulates formaldehyde growth. (A) Evolved isolate (CM3035, pink), reconstructed *efgA*^*evo1*^ mutant (CM3793, red), and Δ*efgA* mutant (CM3745, black triangles) were grown in liquid Hypho medium with 5 mM exogenous formaldehyde as a sole source of carbon and energy. (B) The Δ*efgA* mutant strain was grown in liquid MP medium with 0 (i.e., no carbon), 4, 6, 8, or 10 mM exogenous formaldehyde as a sole source of carbon and energy. For comparison, representative growth with 15 mM methanol (no formaldehyde) is shown (“M”). Error bars represent the standard error of the mean of three biological replicates. In Panel A, only the exponential phase of growth is depicted; in Panel B, measurements are stopped upon entry into stationary phase. The original data shown in this and all other figures are available in Supporting information file S1 Data.

In order to understand how *efgA* is involved in formaldehyde growth, we generated a series of mutants and characterized their phenotype in growth media containing varying concentrations of formaldehyde as the sole carbon and energy source. Introduction of *efgA*^*evo*^ alleles from evolved isolates into the wild-type background was sufficient to enable growth in medium containing up to 5 mM formaldehyde (Figs 3 and 4A). Conversely, restoration of the *efgA*^WT^ allele in an evolved isolate abolished formaldehyde growth, demonstrating the necessity of *efgA*^*evo*^ for formaldehyde growth (Fig 3). Taken together, these data show that *efgA*^*evo*^ alleles were necessary and sufficient for growth on formaldehyde. Like the evolved alleles, a Δ*efgA* in-frame deletion allele in the wild-type background indicated that the EfgA function must be eliminated to permit growth on formaldehyde. These data, as well as the spectrum of mutations obtained for the *efgA*^*evo*^ alleles, indicate that these are loss-of-function mutations.

Further phenotypic analysis of the Δ*efgA* in the wild-type background showed that it enabled formaldehyde growth in medium containing 4 to 8 mM formaldehyde at initial growth rates comparable to those seen on methanol (Fig 4). Here, we noted that the maximum cell density was highest with 8 mM formaldehyde and abolished with 10 mM formaldehyde. The Δ*efgA* strain also exhibited increased formaldehyde resistance in the presence of other growth substrates such as succinate and methanol (S5 Fig). In these conditions, increased formaldehyde concentrations again resulted in modest increases in bacterial density until formaldehyde became inhibitory, suggesting that when tolerated, formaldehyde was serving as a secondary growth substrate. Correspondingly, introduction of a second copy of *efgA*^WT^ in the chromosome of wild-type resulted in increased sensitivity to formaldehyde in the presence of an alternative growth substrate such as succinate (S6 Fig). These data show that formaldehyde growth was due to increased formaldehyde resistance and that *efgA* plays a key role in the cell’s response to formaldehyde.

### EfgB plays a secondary role in formaldehyde resistance

*efgB* encodes a putative adenylate/guanylate cyclase, a group of regulatory proteins that synthesize cyclic nucleotide second messengers. Adenylate/guanylate cyclases are well known to exist in essentially all organisms and have been associated with a wide variety of phenotypes, ranging from catabolite repression, induction of virulence, and stress response [98–100]. EfgB displays low identity to CyaA of *E*. *coli* (6.5%) with its most closely related homologs found in other members of the Rhizobiales with diverse physiologies (S7 Fig), rather than methylotrophic organisms from a wide variety of phylogenetic groups.

Genetic analyses with *efgB* alleles indicate that EfgB also plays a role in growth on formaldehyde. Consistent with *efgA*^*evo*^ alleles being necessary and sufficient for formaldehyde growth, the introduction of *efgB*^*evo*^ alone into the wild-type background did not confer growth on 5 mM formaldehyde (Fig 3). When *efgB*^WT^ was introduced into an evolved strain to replace the *efgB*^*evo1*^ allele, no detectable change in growth was observed in medium containing up to 5 mM formaldehyde (Fig 3); however, when Δ*efgB* was introduced into any of the evolved strains, growth at higher concentrations of formaldehyde was decreased (S8 Fig). These data indicate that the *efgB*^*evo*^ alleles are gain-of-function mutations and led us to hypothesize that they could increase formaldehyde resistance in a genomic background that had already achieved formaldehyde growth.

**Fig 5:**
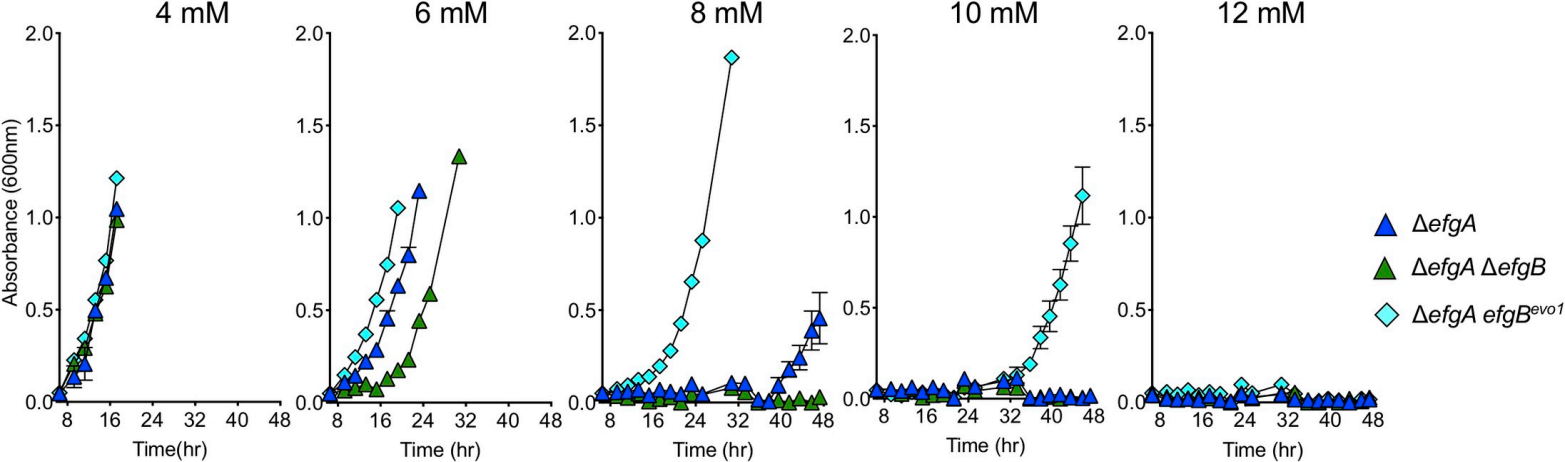
Formaldehyde resistance of *efgA efgB* double mutants indicates *efgB*^*evo1*^ is a gain-of-function allele. The Δ*efgA* (blue triangles), Δ*efgA* Δ*efgB* (green triangles), and Δ*efgA efgB*^*evo1*^ mutants (cyan diamonds) were grown in liquid MP medium with 15 mM succinate and 4, 6, 8, 10, or 12 mM exogenous formaldehyde. Error bars represent the standard error of the mean for three biological replicates. Growth measurements were stopped when absorbances neared the upper limits of the operational range of the spectrophotometer. The original data shown in this and all other figures are available in Supporting information file S1 Data.

To more clearly elucidate the role of EfgB, we further examined different *efgB* alleles in the Δ*efgA* and wild-type backgrounds. In the Δ*efgA* background, growth analysis showed that, at higher concentrations of formaldehyde (6 to 10 mM), the state of the *efgB* allele affected growth in the order *efgB*^*evo*^>e*fgB*^*WT*^>Δ*efgB* (Fig 5). In the wild-type background, strains with *efgB*^*evo*^ alleles exhibited modest resistance to formaldehyde but only when tested at the low concentration of 2 mM formaldehyde in the presence of a suitable growth substrate such as succinate (S9 Fig). These data indicate that the *efgB* alleles did not impact growth in the absence of formaldehyde stress but do increase formaldehyde resistance.

### EfgA has a formaldehyde-specific role in methylotrophy

Phenotypic analyses of the Δ*efgA* mutant indicate the impact of EfgA is specific to formaldehyde stress. During growth on methanol or succinate alone, the Δ*efgA* mutant phenotype was indistinguishable from wild type with regard to lag time, growth rate, and maximum cell density reached upon entry into stationary phase (S5 Fig). Inhibitory levels of several other aldehydes (glyoxal, acetaldehyde, glutaraldehyde, butyraldehyde, propionaldehyde) did not exhibit a differential effect on Δ*efgA* strains compared to wild type (S10 Fig). These data suggest that EfgA has a formaldehyde-specific role in the cell.

Analysis of *efgB* mutants indicate that EfgB is involved in a broad stress response and not limited to formaldehyde stress. None of the *efgB* alleles tested affected growth on methanol or succinate alone (S9 Fig). In contrast to the results with the Δ*efgA* strain, *efgB*^*evo*^ alleles conferred modest resistance to a number of aldehydes in addition to formaldehyde (S11A Fig). This broader stress resistance extended beyond aldehydes, as *efgB*^*evo*^ alleles provided resistance to heat shock as well as a few antibiotics (S11B and S11C Fig).

Taken together, our data indicate that EfgA has a formaldehyde-specific role in the cell, whereas EfgB is involved in resistance to multiple stressors. The specific association of homologs of EfgA, but not EfgB, with methylotrophy further corroborates this formaldehyde-specific role. Our data definitively show that EfgA plays a primary role in formaldehyde resistance unlike EfgB, which plays a secondary role. Correspondingly, we focused our efforts to uncover the biochemical function and role of EfgA.

### The crystal structure of EfgA and molecular dynamics simulations suggest a formaldehyde-binding pocket

**Fig 6:**
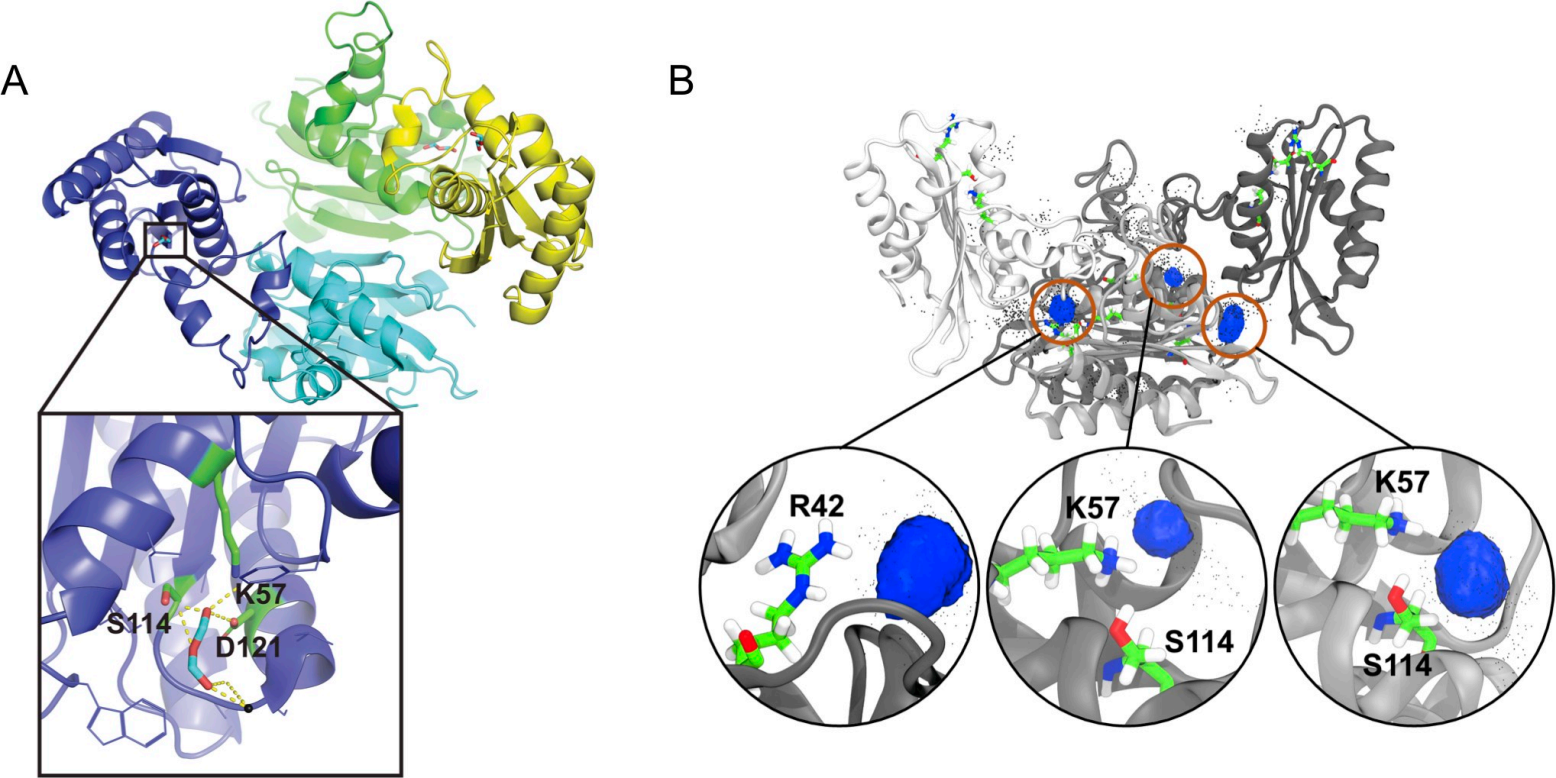
X-ray crystal structure of EfgA and predicted formaldehyde binding sites. (A) EfgA tetramer with each chain highlighted in different colors. Inset: enlarged view of the EfgA binding pocket showing 3 crucial amino acids, K57, D121, and S114, potentially involved in H-bond interaction with formaldehyde in the crystal structure. (B) EfgA tetramer with each chain highlighted in shades of gray with all 2,020 docked poses of formaldehyde and two key amino acids, K57 and S114. Each pose is represented by a single carbon atom (black points) of formaldehyde. Blue clusters demonstrate highly populated regions. Orange circles show an enlarged view of each distinct site. Residues at each site are elementally color-coded, with carbon = green, oxygen = red, nitrogen = blue, and hydrogen = white.

To further develop hypotheses regarding the biochemical function of EfgA, we determined the structure of an N-terminal His-tagged derivative. EfgA diffracted to 1.65 Å resolution (PDB: 6BWS). The tertiary structure of individual protomers is comprised of an antiparallel β-sheet flanked by 4 antiparallel α-helices in a mixed topology (S12 Fig). The packing of the monomers is consistent with a homotetrameric quaternary structure (Fig 6A). No cofactors or metals copurified with EfgA. With these structural data in hand, we employed a MD+FoldX approach [79] to rationalize the *efgA*^*evo*^ alleles that emerged. This analysis suggested that 11 of the 14 nonsynonymous *efgA* mutations either decrease the stability of monomers or of the interactions between them (all but H2Q, M107I, S114N; S13 Fig).

Search for a structural homolog via DALI revealed that the C_α_ positions of the EfgA monomer have a root-mean-square deviation of 1.95 Å (with 127 atoms aligned) for the C_α_ positions of the monomeric chain of HbpS from *S*. *reticuli* (PDB: 3FPV, Fig 2A). HbpS and EfgA only share 23% amino acid identity, and EfgA lacks the twin-arginine translocation signal sequence present in HbpS (S14 Fig). We did not expect HbpS and EfgA to have identical functions but were intrigued that EfgA may also function as a stress sensor, specifically hypothesizing that it may sense formaldehyde.

X-ray crystallography of formaldehyde-soaked crystals indicated a specific site where formaldehyde may bind. The structure of the protein with formaldehyde as a potential complexed ligand resolved at 1.83 Å resolution (PDB: 6C0Z). The overall structure was largely unchanged from the apo-protein, but the difference maps comparing the apo and formaldehyde complexes indicated new electron density in the formaldehyde-soaked crystals. The additional electron density was localized to a specific binding pocket of each monomer. In three EfgA monomers, new densities were modeled as oxydimethanol, and in the fourth monomer, the electron density was modeled as formate (Fig 6A). Both oxydimethanol and formate are derivatives of formaldehyde and are structurally homologous. Potential hydrogen bonds were observed between ligands and amino acids that corresponded to S114, D121, and K57 in the native EfgA protein. (Fig 6A, inset).

Consistent with the structural data from formaldehyde-soaked crystals, MD simulations identified the identical pocket as the likely site of formaldehyde binding. Unbiased docking calculations were performed to dock formaldehyde to snapshots obtained from 100 ns MD simulation initiated from the X-ray crystal structure of apo-EfgA tetramer. In total, 2,020 docked poses were captured, and regions of high-density poses were found to correspond with the binding pockets identified during formaldehyde crystal soaks (Fig 6B). In addition to the primary interaction (K57, D121, and S114), these calculations suggest a feasible interaction at R42. The potential importance of the K57-D121-S114 pocket is emphasized by the fact that one of the loss-of-function *efgA*^*evo*^ alleles (EfgA^S114N^) modifies one of these 3 proposed binding interactions.

### In vitro EfgA:ligand interaction demonstrates direct, specific binding to formaldehyde

**Fig 7:**
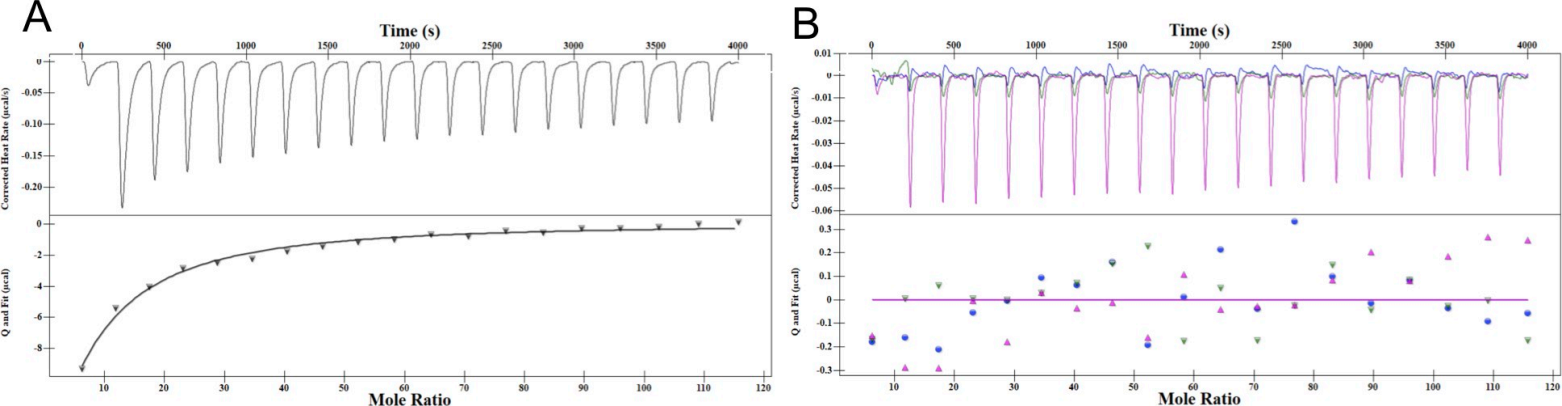
Microscale isothermal calorimetry indicates EfgA binds formaldehyde but not methanol, formate, or acetaldehyde. The binding isotherms represented as heat change (μJ/s) upon injection over time are in the top portion of the split graph, with independent binding modeling on the bottom portion. (A) Binding observed with 50 μM EfgA (black) and 2 μL injections of 25 mM formaldehyde (in PBS). (B) Binding observed with 50 μM EfgA and 2 μL injections of 25 mM methanol (blue), formate (green), acetaldehyde (pink). Data are representative of trends observed in multiple experiments (*n =* 3); additional replicates are shown in S15 Fig. The original data shown in this and all other figures are available in Supporting information file S1 Data.

To directly test the hypothesis that EfgA senses formaldehyde by direct binding, as suggested by the structural and biophysical modeling evidence above, we used 2 independent biophysical approaches. mITC binding isotherms showed that formaldehyde binding for native EfgA was exothermic (ΔH = −22.65 ± 1.16 kcal/mol), suggesting formaldehyde binding was favorable. Boiled EfgA had a 23-fold decrease in the enthalpy of binding and broadened isotherms, suggesting that the strong interaction of formaldehyde with EfgA required the native 3-dimensional structure, rather than nonspecific interactions (Fig 7A, S15A Fig). MST was used to both independently confirm the interaction of EfgA with formaldehyde and permit calculation of its affinity (K_d_ = 8.01 ± 3.5 mM) (S16 Fig).

We tested EfgA interactions with two additional categories of alternative ligands that are structurally similar: other C_1_ intermediates (methanol and formate) and a longer aldehyde (acetaldehyde). mITC results indicate no evidence of binding to methanol, formate, or acetaldehyde (Figs 1 and 7B, S15D–S15F Fig). Together, these biochemical data confirm the hypothesis that EfgA specifically binds formaldehyde.

### EfgA homologs from methylotrophs are functionally redundant

**Fig 8:**
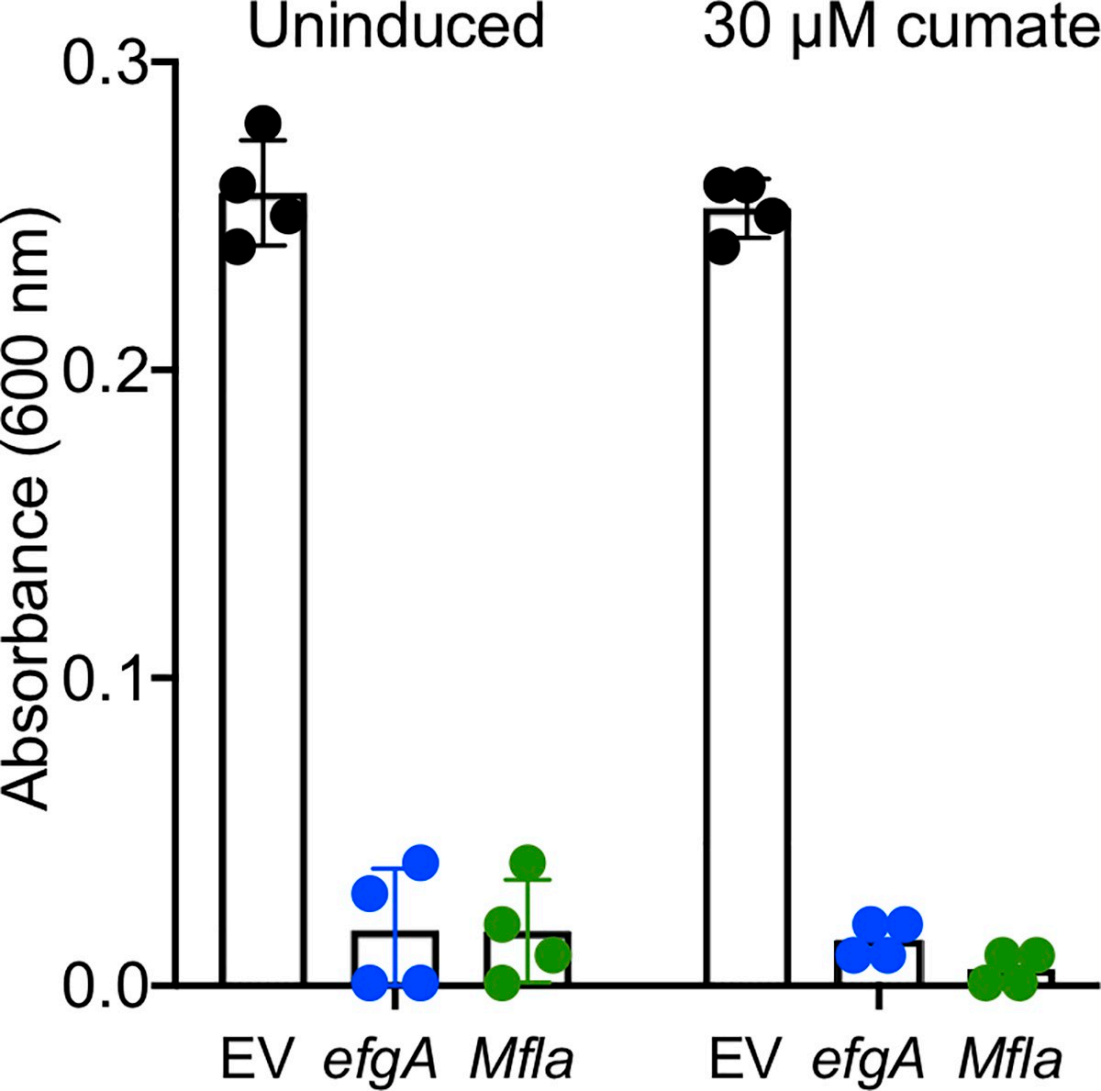
EfgA from *Methylobacillus flagellatus* complements the *M*. *extorquens* Δ*efgA* mutant. Derivatives of the Δ*efgA* mutant containing pLC290 were grown in liquid MP medium with 8 mM formaldehyde as a sole source of carbon and energy. Strains contained expression plasmids that were either an empty vector control (CM4625, EV, black), expressed EfgA from *M*. *extorquens* (CM4126, *efgA*, blue), or the homolog from *M*. *flagellatus* (CM4182, *Mfla_1444*, green). Expression from pLC290 is cumate-inducible [133]; uninduced were grown without the addition of cumate. Error bars represent the standard error of the mean for four biological replicates. The original data shown in this and all other figures are available in Supporting information file S1 Data.

Having demonstrated EfgA from *M*. *extorquens* binds formaldehyde, we hypothesized that EfgA homologs found in methylotrophs would have a conserved formaldehyde-sensing function. We attempted to complement a Δ*efgA* strain of *M*. *extorquens* (an alphaproteobacterium) with *Mfla_1444*, the corresponding gene from *Methylobacillus flagellatus* KT (a betaproteobacterium). Mfla_1444 is an EfgA homolog with 67% identity and 76% similarity and represents a member of the second major clade of EfgA sequences in comparison to the EfgA from *M*. *extorquens* (Fig 2B). Even the basal, uninduced expression of *Mfla_1444* complemented a Δ*efgA* strain and inhibited formaldehyde growth as well as the native gene (Fig 8). These data suggest that the clade of EfgA homologs found throughout methylotrophs sense formaldehyde and are competent in transmitting that signal to conserved downstream components.

### EfgA protects cells from endogenous formaldehyde stress

The direct interaction of EfgA with formaldehyde and its conserved function across methylotrophs led us to question what selective benefit EfgA may provide to cells. The experimental evolution conditions we used had selected for the “removal” of EfgA function to allow cells to grow on high concentrations of formaldehyde as a sole carbon source. These conditions are unlikely to be ecologically relevant as they were both extreme and involved exogenous formaldehyde rather than endogenous production as a pathway intermediate. Thus, we hypothesized that the advantage of EfgA regulation may be when methylotrophs experience misbalanced intracellular formaldehyde.

**Fig 9:**
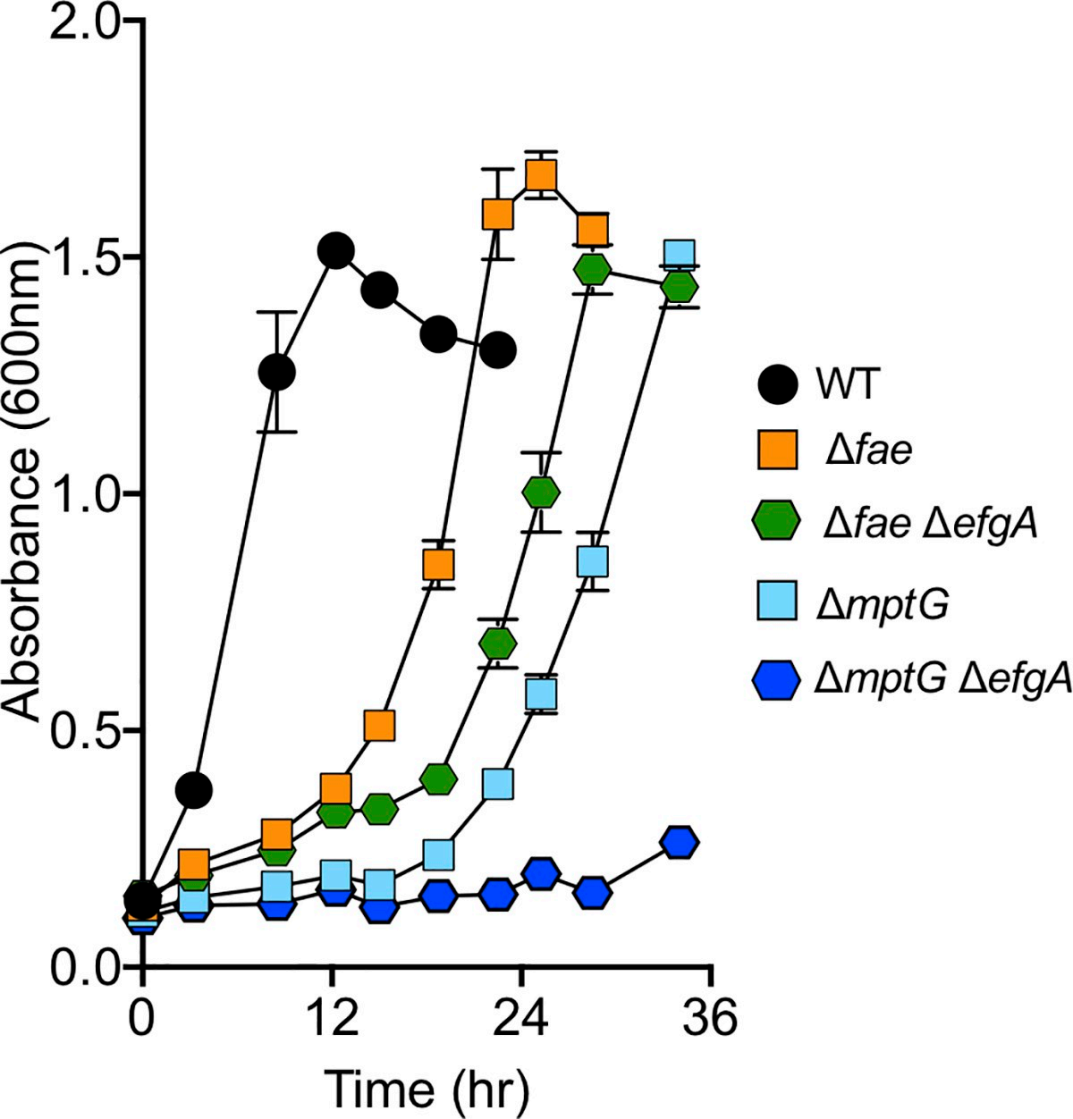
EfgA provides protection from internal formaldehyde in methanol sensitive mutants. Wild-type and mutant strains were grown to early exponential phase in liquid MP medium (succinate) at which point 1 mM methanol was introduced into the medium (t = 0 h). Strains represented are wild-type (black circles), Δ*fae* (CM3753, orange squares), Δ*mptG* (CM4765, light blue squares), Δ*efgA* Δ*fae* (CM3421-5, green hexagons), and Δ*efgA* Δ*mptG* mutants (CM3440-13, blue hexagons). Error bars represent the standard error of the mean for three biological replicates. For evidence that growth of all strains on succinate (without methanol treatment) is comparable, see S18 Fig. The original data shown in this and all other figures are available in Supporting information file S1 Data.

Taking advantage of mutants with partial (Δ*fae*) or complete (Δ*mptG*) lesions in the dH_4_MPT pathway that are predicted to generate internal formaldehyde stress, we determined that EfgA is beneficial under these conditions. During exponential growth on succinate, we found that Δ*fae* mutants accumulated internal formaldehyde when treated with 1 mM methanol, confirming that disrupting the dH_4_MPT pathway is correlated to an increase in intracellular formaldehyde (S17 Fig). Δ*efgA* Δ*fae* and Δ*efgA* Δ*mptG* strains displayed an exacerbated growth defect compared to the corresponding single mutants with *efgA*^WT^ when 1 mM methanol was added during growth on succinate (Fig 9). These data showed EfgA is beneficial when cells experience endogenous formaldehyde stress. Furthermore, they indicate that the EfgA-mediated response is independent of the dH_4_MPT pathway enzymes and metabolic intermediates.

### EfgA is not linked to phenotypic formaldehyde tolerance

**Fig 10:**
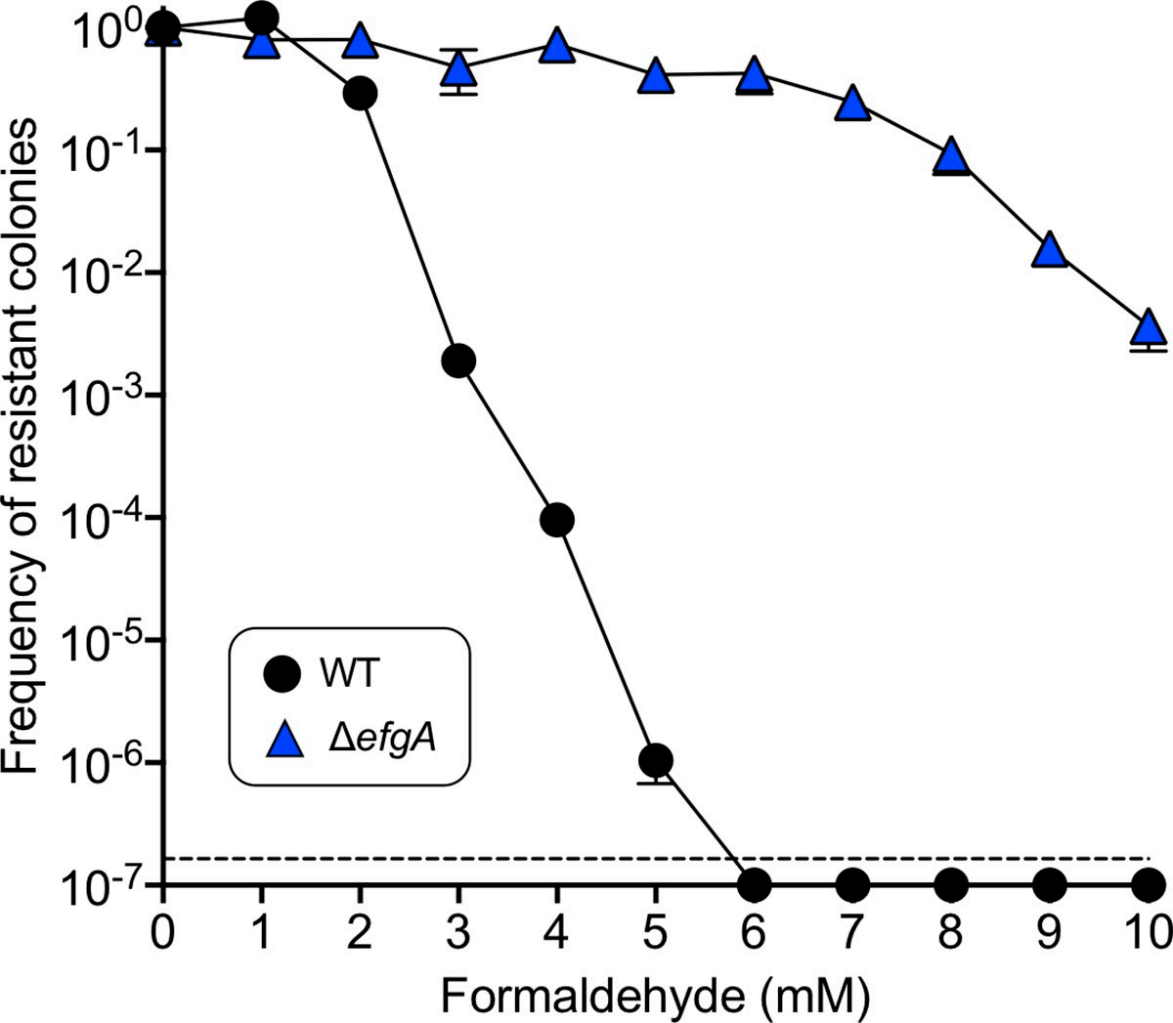
EfgA influences phenotypic formaldehyde tolerance distribution in *M*. *extorquens*. The distribution of formaldehyde tolerances among individual cells was assessed in WT (black solid circles) and Δ*efgA* (blue triangles) populations. Stationary-phase cultures were plated onto MP-methanol agar medium containing a range of formaldehyde concentrations at 1 mM intervals. The frequency of tolerant cells is expressed as the ratio of the CFU enumerated at the given formaldehyde concentration to the CFU enumerated on formaldehyde-free (0 mM) medium. Error bars represent the mean standard deviation of three replicate plates; the horizontal dotted line denotes the limit of detection. The original data shown in this and all other figures are available in Supporting information file S1 Data. CFU, colony-forming units; MP, *Methylobacterium* PIPES; WT, wild-type.

Given that mutations in *efgA* impact genetic resistance to formaldehyde, we questioned whether it was linked to phenotypic formaldehyde tolerance observed between genetically identical cells [35]. *efgA* was not represented among the differentially expressed genes seen in the environmentally responsive phenotypic variation in formaldehyde tolerance (S2 Table). Additionally, neither *efgB* nor any of the other loci identified via mutations during selection for formaldehyde growth had significant expression changes, other than a single modest change in a secondary locus yet to be investigated (*Mext_2112*, encoding an XRE family transcriptional regulator/shikimate kinase; 1.19-fold change, p-adj = 9.28 E-05) (S2 Table). A comparison of the tolerance distributions of wild-type and the Δ*efgA* mutant showed that a distribution of formaldehyde tolerance was maintained within populations of the Δ*efgA* mutant but was shifted toward higher tolerance (Fig 10). Notably, the qualitative shape of the distribution altered with the rate of decline being decreased in the Δ*efgA* mutant (slope: WT = −4.112 log_10_ cells / mM formaldehyde, Δ*efgA* = −1.482, *p*-value = 0.0004). Further work will be required to determine whether their protein levels or activities may play any role in this response.

### EfgA can provide protection from formaldehyde in the non-methylotroph *E*. *coli*

Does EfgA only function in methylotrophs, or might it perhaps provide protection to a heterologous host that is not a methylotroph? Several pieces of evidence led us to hypothesize that this might be possible. First, EfgA senses formaldehyde directly and does not require a functional C_1_ pathway; these functions should be possible in a different genomic context. Second, we demonstrated that a phylogenetically distinct EfgA homolog can complement a Δ*efgA* mutant, suggesting that any downstream signaling system might be broadly conserved. Third, unlike other DUF336 homologs (e.g., *hbpS*, *pduO*, *glcC*), there does not appear to be a conserved genomic context for *efgA* across methylotrophs, suggesting it may have been introduced on its own in the history of those lineages.

**Fig 11:**
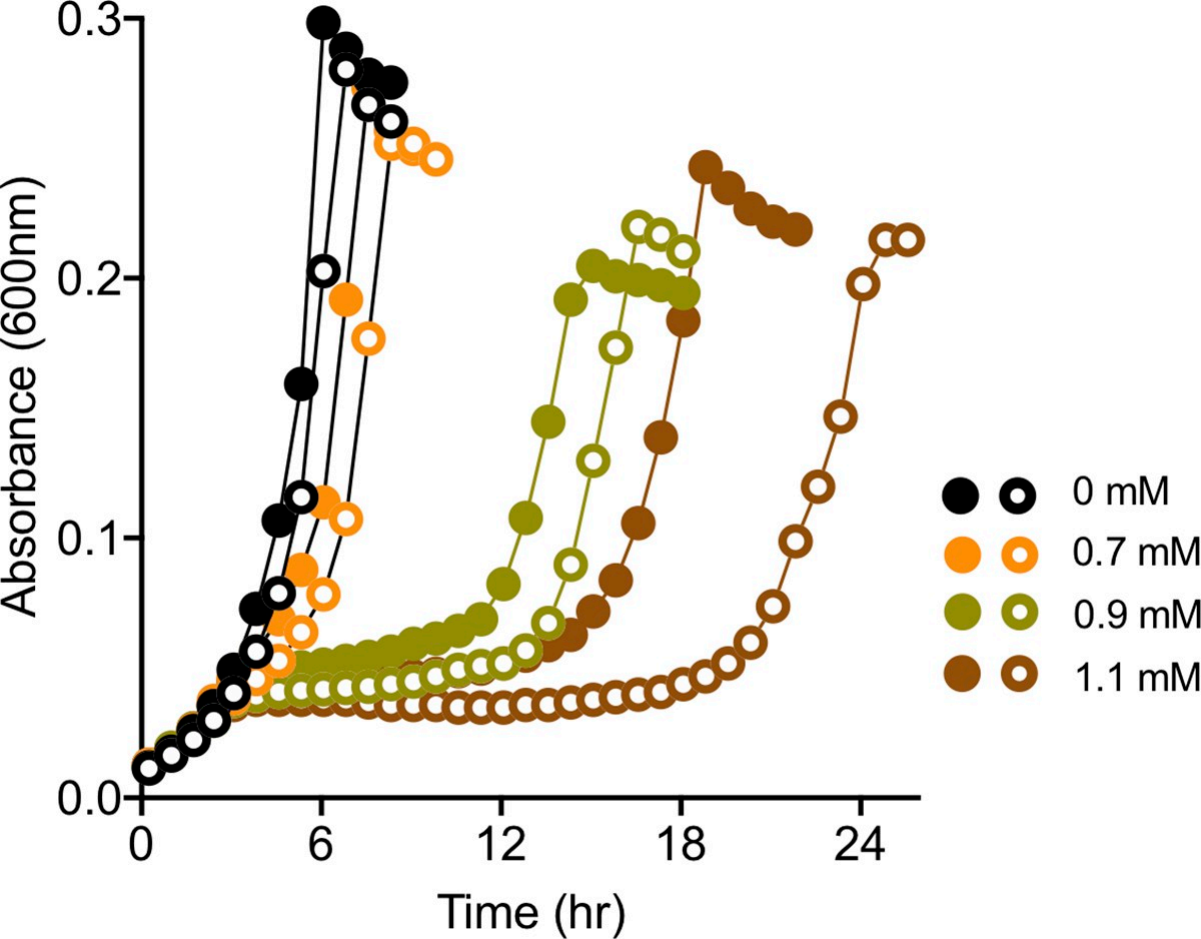
EfgA provides protection to *E*. *coli* strains during formaldehyde exposure. Growth of *E*. *coli* was quantified in liquid MOPS medium with 2 mM glucose and 0, 0.7, 0.9, or 1.1 mM exogenous formaldehyde added after 2.33 h. Strain WM8637 (filled symbols) harboring a chromosomal insert of *efgA* under the control of P_*rhaS*_ or the empty vector control (WM8653, empty symbols) was grown in comparable conditions. *efgA* expression was induced with 0.5 mM rhamnose. Data are representative of trends observed in multiple experiments. The original data shown in this and all other figures are available in Supporting information file S1 Data.

Heterologous expression of EfgA in *E*. *coli* (a gammaproteobacterium) demonstrated EfgA can provide increased formaldehyde resistance in a novel organism. *E*. *coli* grown in minimal MOPS medium with glucose displays sensitivity to >0.7 mM formaldehyde, but strains expressing EfgA showed a decreased lag time across the formaldehyde concentrations tested (Fig 11). The ability of EfgA to mitigate formaldehyde stress in a new organism, in the absence of native methylotrophic machinery, supported our hypothesis. These data also suggest one of two scenarios exist: EfgA acts independently or EfgA elicits a cellular response via interactions with proteins that are present and sufficiently conserved across non-methylotrophs to enable fortuitous interactions to occur.

### Alternative loci that permit growth on formaldehyde in the presence of active EfgA include two ribosome-associated proteins

Despite having a functional EfgA, four other loci were targets of beneficial, first step mutations that permitted growth on formaldehyde in some of the evolved populations. To glean information about possible downstream effects of EfgA, we resequenced the genomes of isolates from the seven (of 25) populations that rapidly evolved growth on 5 mM formaldehyde but had an *efgA*^WT^ allele. This identified four loci: *potG* (*Mext_4194*, encoding a putative putrescine transporter), *Mext_0925* (encoding a MarR family transcriptional regulator), *prmA* (*Mext_4479*, encoding ribosomal protein L11 methyltransferase), and *def* (*Mext_1636*, encoding peptide deformylase, PDF) (Table 1, S1 Table). An evolved isolate representing each of these four genes was found to have fairly similar fitness (within 10%) to a reconstructed *efgA*^*evo*^ mutant strain during growth on 5 mM formaldehyde (S19 Fig). While the role of PotG and the MarR-like regulator remain unclear, the finding that mutations in two different genes encoding ribosome-associated proteins (PrmA and PDF) could protect cells from formaldehyde led us to further explore the unexpected connection between formaldehyde stress and translational events.

### *N*-formylmethionine pathway contributes to formaldehyde resistance

**Fig 12:**
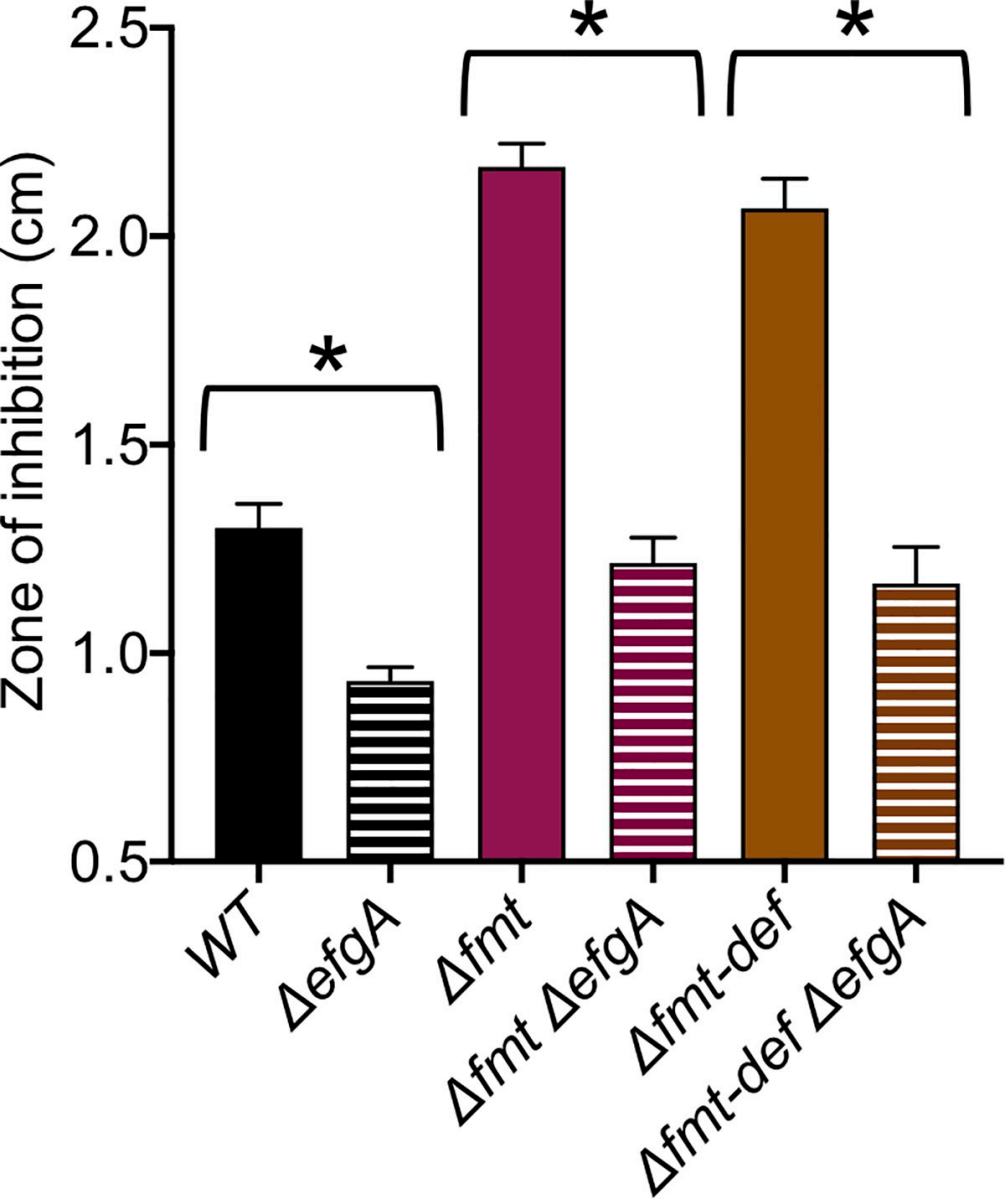
EfgA-mediated formaldehyde resistance is not dependent on PDF. Disc-diffusion assays were performed by placing formaldehyde-impregnated discs upon soft agar overlays of *M*. *extorquens* on solid MP media (125 mM methanol). The zones of inhibition observed showed that like wild type, mutants lacking the fMet-mediated protein degradation signal (Δ*fmt*, Δ*fmt-def*) are more formaldehyde-resistant when *efgA* is also deleted. Wild-type and Δ*efgA* mutant were included as experimental controls. Error bars represent the standard error of the mean for three biological replicates. Statistical significance was determined by an unpaired Student *t* test (*, *p* < 0.05). The original data shown in this and all other figures are available in Supporting information file S1 Data.

The observation that variants of PDF allowed formaldehyde growth led us to hypothesize that the *N*-formylmethionine (fMet) pathway plays a role in formaldehyde resistance. During translation, PDF removes the formyl moiety from the fMet at the N-terminus of a majority of the nascent peptides in bacteria. We were unable to delete *def* in wild-type *M*. *extorquens*, consistent with the finding that *def* is often individually essential [101–103]. In other bacteria, mutants lacking methionyl-tRNA formyltransferase (encoded by *fmt*) do not synthesize fMet and no longer require PDF [102–105]. In a wild-type background, deletions of *fmt* alone or the entire *fmt-def* operon significantly increased sensitivity to formaldehyde (Fig 12). To test whether the lack of fMet precludes the effects of EfgA activity, the Δ*fmt* and Δ*fmt-def* alleles were combined with the Δ*efgA* allele. The resulting strains showed that the deletion of *efgA* significantly increased resistance even in the absence of the fMet pathway (Fig 12). These results indicate that EfgA must act, at least in part, independent of the N-terminal protein formylation pathway, but further implicate protein translation/maturation as having a key role in formaldehyde resistance.

### EfgA rapidly halts growth in response to elevated formaldehyde

**Fig 13:**
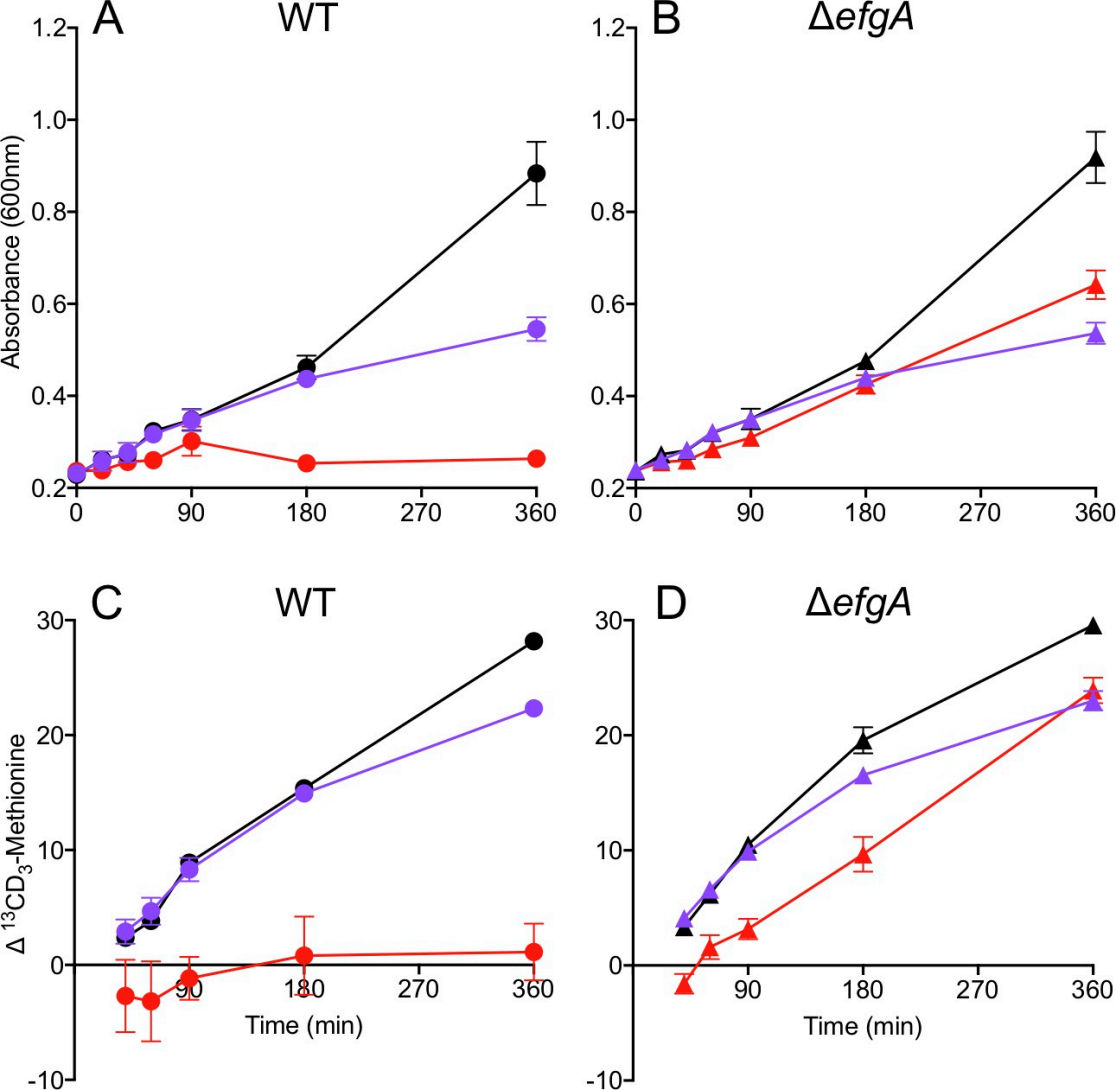
EfgA causes rapid growth arrest and cessation of translation in response to formaldehyde. Exponential phase cultures of wild-type (circles, A, C) and Δ*efgA* mutant (triangles, B, D) strains were treated with kanamycin (purple), exogenous formaldehyde (red) or left untreated (black). Top panel: Growth in response to treatment (t >0 min) was monitored. Bottom panel: After treatment (t >0 min) in vivo translation was assayed via [^13^CD_3_]-methionine incorporation. Error bars represent the standard error of the mean for three biological replicates. The original data shown in this and all other figures are available in Supporting information file S1 Data.

Multiple findings led us to directly test the effects of EfgA on growth and translation. During exponential growth on succinate, where formaldehyde is not produced, the impact of formaldehyde on wild-type and an Δ*efgA* mutant was assessed by monitoring growth and translation in response to exogenous formaldehyde shock. Translation was assayed in vivo by tracking the incorporation of exogenously provided [^13^CD_3_]-Met into cellular proteins of wild-type and an Δ*efgA* mutant by GC/MS. Treating cells with kanamycin, a known translation inhibitor, reduced methionine incorporation by the 360 m time point in both genotypes and did not appear to immediately impact growth (Fig 13). In contrast, formaldehyde-treated wild-type samples halted growth and translation immediately, showing no detectable increase in absorbance or methionine incorporation with statistical significance for the full 360 m (Fig 13). For the formaldehyde-treated Δ*efgA* mutant, growth continued in spite of treatment and accordingly, translation was not halted and increased between all time points. Overall, the effects of treatments were quite distinct; kanamycin inhibition being slow and formaldehyde inhibition in the presence of EfgA being rapid (Fig 13). The addition of formaldehyde to the Δ*efgA* mutant did not induce growth arrest and only led to a modest growth defect. These data indicate that, although formaldehyde does exert some inhibition of growth and translation on its own, the primary effect of excess formaldehyde upon growth and translation is mediated by EfgA.

## Discussion

Formaldehyde detoxification systems have been identified in all domains of life [106]. Methylotrophs generate formaldehyde at high rate, yet several decades of work with methylotrophs had failed to reveal any proteins that sense and respond to its toxicity. By evolving *M*. *extorquens* to grow on 20 mM formaldehyde as a sole substrate, a concentration that was found to sterilize a wild-type culture in just 2 h [35], we uncovered several genes that could permit growth. Notably, we report here that one of these, now named *efgA* for **e**nhanced **f**ormaldehyde **g**rowth, encodes a formaldehyde sensor that can act through inhibition of growth.

We propose that the role of EfgA is to impart protective growth stasis when formaldehyde is transiently elevated to protect cells from formaldehyde damage. Metabolite-mediated growth and translational arrest has been previously reported for metabolites involved in translation itself, such as amino acids or ppGpp, but not for endogenous stressors [107]. Such a mechanism could limit formaldehyde-induced damage to cellular components, spare protein resources under stress conditions [108], or even reduce further enzymatic production of formaldehyde during high formaldehyde exposure. Nascent peptides emerging from ribosomes may be among the most consequential sites of damage and toxicity of formaldehyde crosslinking. Although the mechanism of EfgA action remains unclear, the consequent translational inhibition may be effective because it provides a rapid mechanism for inducing growth arrest. Sustained high external concentrations of formaldehyde that induced permanent growth arrest in wild type are quite unlikely to be encountered in nature; however, the ability of EfgA to help cells navigate elevated internal formaldehyde stress may be critical in a fluctuating environment. *M*. *extorquens* are commonly isolated as key members of the leaf microbiome. Although formaldehyde concentrations in plant tissues are relatively low (0.1 to 10 μmol/g in *Zea mays* and 4.2 μmol/g in *Arabidopsis thaliana*) [109,110], methanol is generated and released in pulses each day [111,112]. This raises the possibility that the beneficial role of EfgA is to mitigate formaldehyde toxicity when formaldehyde production from methanol dehydrogenase and usage by the dH_4_MPT pathway become transiently imbalanced. Such imbalances might also arise from metabolic perturbations that can be caused by stressors [113,114], metabolic crosstalk [115], transcriptional bursts [116], or nutrient limitation/shifts [31,117]. Consistent with this suggestion, we have recently discovered that EfgA is one of two loci that we have found are required for the efficient transition from multi-C to C_1_ growth [118].

The *efgA*-related phenotypes are paradoxical as they simultaneously suggest that EfgA can protect cells from formaldehyde damage through growth stasis and yet in its absence cells are more resistant to exogenous formaldehyde. As EfgA reduces growth and translation, our data suggest that when EfgA is activated by endogenously produced formaldehyde, cells with reduced growth and translation may be able to avoid what would otherwise be the beginning of a cascade of damage. Data from our recent work indicated that a major part of the transcriptional response to formaldehyde stress appeared to be an up-regulation of chaperone-encoding genes and the response shared significant overlap with that of kanamycin, a classical translation inhibitor [119]. Chaperone-encoding genes also had increased transcription in cells with increased phenotypic tolerance to formaldehyde [35]. However, translation inhibition can be a double-edged sword, and we speculate that the heightened sensitivity of wild type to exogenous formaldehyde may represent a tipping point where inhibition of growth and translation cannot be reversed and cell death occurs as a result.

Both mITC and MST results confirmed that EfgA binds formaldehyde (Fig 7, S15 Fig) but with a relatively high K_d_ of 8 mM (S16 Fig). Given that the intracellular concentration of formaldehyde during growth of *M*. *extorquens* during steady-state growth on methanol has been estimated to be 1 mM [34], a high K_d_ above that concentration would ensure that EfgA occupancy would remain unsaturated during methylotrophic growth, leaving the opportunity for a dynamic response when formaldehyde concentrations rise further. Furthermore, the specificity of EfgA binding of formaldehyde (Fig 7, S15 Fig) would render EfgA insensitive to the free C_1_ intermediates upstream and downstream of it, as well as acetaldehyde that would be generated during growth on C_2_ compounds, such as ethanol.

Curiously, the additional electron densities that arose in the formaldehyde-soaked crystals were more consistent with the formaldehyde derivatives, oxydimethanol and formate and not formaldehyde itself. Each of these compounds are derived from formaldehyde and can spontaneously form in aqueous formaldehyde solutions [120]. Aqueous solutions of formaldehyde are composed of formaldehyde and formaldehyde-water derivatives (methylene glycol and oxydimethanol) that exist in equilibrium. Formaldehyde crystal soaks were performed at 10°C, where the molar ratio of oxydimethanol is nearly at its maximum (0.6 at 8.3°C) [120] and the molar ratio of formaldehyde is approximately 0.3. Therefore, we suspect that the presence of oxydimethanol was an artifact of the experimental conditions but, in fact, its localization is the physiologically relevant site of formaldehyde in the binding pocket. Alternatively, it is possible that EfgA does not sense the unmodified form of formaldehyde directly, but rather an adduct/derivative only formed when formaldehyde is present at sufficiently high concentrations (e.g., oxydimethanol) and which could serve as a proxy for formaldehyde itself. The presence of formate in one of the four monomers may represent an oxidation product formed during crystallization; the lack of interaction seen via mITC when testing formate directly argues against it being the genuine ligand in vivo. Taken together, our results validated the protein:ligand interaction as distinct from nonspecific binding that might be expected from the ability of formaldehyde to nonspecifically form adducts on and crosslink amino acids residues.

Both X-ray crystallography and in silico approaches to dock formaldehyde support the hypothesis that formaldehyde binding demonstrated empirically occurs in a pocket formed via hydrogen bonds with S114, D121, and K57. Fortuitously, a substitution in one of these sites, EfgA^S114N^, was isolated as one of the loss-of-function alleles isolated from one of the evolving populations. Combined with the high degree of conservation at these three residues (S14 Fig), these are top candidates for residues involved in ligand binding and will be investigated further in future work.

The discovery that EfgA is a formaldehyde sensor indicates a previously unknown role for DUF336 proteins in sensing small molecules. Proteins with DUF336 domains are present in single or multidomain proteins and their genetic association with gene clusters that encode well-characterized pathways such as glycolate utilization in *E*. *coli* (GlcG), 1,2-propanediol utilization in *Salmonella enterica* (PduO), and chloroaniline degradation in *Diaphorobacter* sp. PCA039 (OrfU2) has been noted [85–89]. The two best characterized examples are HbpS of *S*. *reticuli* and PduO of *S*. *enterica*. HbpS is located extracellularly where it senses and degrades heme and activates a two-component system involved in oxidative stress [91,95,121,122]. PduO is localized to the *pdu* microcompartment; its DUF336 domain is fused to an ATP:cob(I)alamin adenosyltransferase domain; its DUF336 domain is not required for the activity of the ATP:Cob(I)alamin adenosyltransferase domain in vitro but is required for optimal 1,2-propanediol utilization for unknown reasons [89]. Both HbpS and PduO have been shown to bind heme and cobalamin, but their cellular locations are distinct, consistent with their disparate functions. In this light, EfgA represents the third biochemically characterized bacterial DUF336 protein and is distinct from HbpS in that it is cytoplasmic, senses formaldehyde, and modulates translation. EfgA and HbpS overlap in terms of being sensors; EfgA and PduO may exhibit functional overlap due to the involvement of an aldehyde in propanediol utilization. Thus, our work, which assigns a novel, aldehyde sensing function for a DUF336 protein, helps to define the broader role of DUF336 domains.

The involvement of several genes with known involvement in translation suggests that controlling protein damage may be particularly critical for cells confronted with formaldehyde stress. Two secondary mutations that we have yet to examine also suggest the key role for protein quality control in formaldehyde resistance. For *hrcA*^*evo*^, the apparent loss-of-function mutation would eliminate HrcA, a heat-inducible transcriptional repressor that negatively regulates heat shock genes [123,124]. The intergenic *Mext_3827*/*Mext_3828* mutation is upstream of *Mext_3828*, annotated as encoding HdeA, a periplasmic chaperone protein that prevents aggregation of periplasmic proteins [125,126]. These mutations suggest that preventing/repairing protein damage may be important for formaldehyde resistance and imply that unchecked formaldehyde stress leads to protein damage in *M*. *extorquens*. Though formaldehyde damage is most commonly associated with DNA damage, there is precedent for formaldehyde-induced protein damage [14,127] which was also suggested by our previous work [35,119].

While the catalytic activity of PDF has long been known, it has only recently been identified as a key player in protein quality control [8,128,129]. Typically, as the fMet of an elongating peptide chain emerges from the ribosome exit tunnel, PDF quickly removes its formyl group, making it a suitable substrate for the downstream processing enzyme. In instances where the elongating peptide is misfolded, fMet is less accessible to PDF and serves as a signal for protein degradation [128]. We isolated *def*^*evo*^ alleles that, through an unknown mechanism, increase formaldehyde resistance, whereas Δ*fmt* and Δ*fmt-def* strains with no fMet cycling decreased formaldehyde resistance. To our knowledge, this is the first indication that fMet modification plays a role in formaldehyde resistance.

The surprising ability for EfgA homologs to influence formaldehyde resistance when introduced between distantly related organisms raises an intriguing possibility that EfgA may directly interact with one or more ribosomal or ribosome-associated components to halt translation. There is precedent for a DUF336 protein to physically interact with ribosome-associated proteins. The homolog in *Saccharomyces cerevisiae*, Ybr137wp, has been shown to be involved in posttranslational control, whereby it binds Sgt2 and Get proteins and is involved in the cotranslational targeting of tail-anchored proteins into membranes [130]. If EfgA interacts with ribosomal or ribosome-associated proteins, it should be noted that molecular composition of ribosomes and the sequences of the molecular components are among most well-conserved aspects of bacteria. The finding that EfgA from *M*. *extorquens* can provide protection from formaldehyde in *E*. *coli* may rely upon the fact that its interaction partners were already encoded and expressed there. Having downstream partners already present in genomes could also explain how *efgA* could be acquired by horizontal gene transfer and integrated into the genome as an isolated gene without conserved synteny. This would be quite distinct from the rampant exchange of methylotrophy pathways by horizontal gene transfer which appear to have been cointroduced as large, genomically clustered modules [131]. The ability for EfgA to provide an immediate benefit in dealing with formaldehyde stress may also have more immediate biotechnological benefits. Given that formaldehyde toxicity was also a key challenge in the engineering and evolution of an *E*. *coli* strain that can grow on methanol as a sole carbon source [132], this raises the possibility that introducing EfgA would increase the cells’ ability to grow while producing formaldehyde as a high-flux intermediate. We perhaps should not be surprised that metabolic pathways that generate toxic intermediates need molecular systems to sense their accumulation and mount responses that either eliminate the toxin, increase the ability to repair such damage, or help the cell avoid making the molecules that the toxin damages in the first place.

## Supporting information

S1 Fig Broad-host-range allelic exchange vector pPS04. Plasmid map of pPS04 [GenBank: MW118672] showing the key features including *kan* (encodes kanamycin resistance), multiple cloning site (MCS) containing a number of single-cutting restriction sites, IncP *oriT* (origin of conjugal transfer), *sacB* (encodes levansucrase for sucrose sensitivity), ColE1 *ori* (high-copy origin of replication for *E*. *coli*).
(TIFF)

S2 Fig Selective regime for the evolution of *M*. *extorquens*. *M*. *extorquens* was experimentally evolved in Hypho liquid media for 150 generations. Initial growth conditions relied on methanol as a sole source of carbon and energy. At each transfer (approximately 6 generations), methanol concentrations were decreased and formaldehyde was introduced into the growth media at increasing concentrations. By generation 35, formaldehyde was the only carbon/energy source present. Formaldehyde concentrations continued to be gradually increased until generation 60 when it reached 20 mM. Selective pressure was sustained at 20 mM formaldehyde until the experiment was completed at 150 generations. The original data shown in this and all other figures are available in Supporting information file S1 Data.
(TIFF)

S3 Fig Individual isolates from formaldehyde-evolved populations grow on formaldehyde up to 20 mM. Five isolates from each of the final formaldehyde-evolved populations (CM3031-CM3045, Table 1) were grown in minimal Hypho medium with 20 mM or 10 mM formaldehyde for 48 h. Plot whiskers indicate the minimum and maximum values. The original data shown in this and all other figures are available in Supporting information file S1 Data.
(TIFF)

S4 Fig Genomic context of *efgA* and *efgB* is not conserved in other organisms. Gene neighborhoods from fully assembled genomes are aligned by (A) *efgA* (blue) or (B) *efgB* (green) and include 10 flanking genes on either side. Organisms represented in Panel A span the EfgA-clade (Fig 2) identified in the phylogenetic analysis of EfgA homologs, while those in Panel B span the phylogenetic distribution of EfgB homolog containing organisms (S7 Fig). Homology between genes is indicated by identical colors among the genomes within each panel. In both panels, genes lacking homologous counterparts in the gene neighborhoods shown are colored tan.
(TIFF)

S5 Fig The absence of *efgA* increases formaldehyde resistance during growth on alternative substrates. Wild-type (CM2730, circles, upper panels) and the Δ*efgA* mutant (CM3745, triangles, lower panels) were grown in liquid MP medium with succinate (left panels) or methanol (right panels) provided as the primary carbon source. Additionally, 0, 2, 4, 6, 8, or 10 mM exogenous formaldehyde was provided as a stressor; however, it can also serve as a secondary carbon source when it is tolerated. Error bars represent the standard error of the mean of three biological replicates. The original data shown in this and all other figures are available in Supporting information file S1 Data.
(TIFF)

S6 Fig Increasing the *efgA* gene dose sensitizes *M*. *extorquens* to formaldehyde. The wild-type (black circles), Δ*efgA* mutant (blue triangles), two-copy *efgA* mutant (gray circles), and Δ*efgA*+chromosomal *efgA* complement (gray triangles) were grown in liquid MP medium with 3.5 mM succinate and 2 mM formaldehyde. Error bars represent the standard error of mean of three biological replicates. All strains were derived from WT, which is Δ*cel*; the second copy of *efgA* was introduced at the Δ*cel* locus. The original data shown in this and all other figures are available in Supporting information file S1 Data.
(TIFF)

S7 Fig Phylogenetic analysis of the EfgB indicates close homologs are in other members of the Rhizobiales with diverse physiologies. The evolutionary relationship of *efgB* was compared to genes with 65%–90% identity via maximum likelihood. Bootstrap values are shown at nodes, and branch lengths reflect the indicate substitutions per nucleotide. The green diamonds represent members whose genomic context is illustrated in S4 Fig. The phylogenetic data are available at TreeBASE (http://purl.org/phylo/treebase/phylows/study/TB2:S27073).
(TIFF)

S8 Fig *efgB* activity is needed for enhanced formaldehyde resistance in evolved isolates. Formaldehyde growth of evolved isolates CM3035 (pink), CM3039 (orange), CM3040 (green), and CM3044 (violet) was measured in liquid MP medium with 6, 8, 10, or 12 mM exogenous formaldehyde provided as a sole source of carbon and energy. Replacing evolved beneficial *efgB* alleles with Δ*efgB* resulted in otherwise isogenic strains CM3739, CM3741, CM3824, and CM3743 (hatched bars) failed to utilize formaldehyde at higher concentrations. Data are representative of trends observed in multiple experiments. The original data shown in this and all other figures are available in Supporting information file S1 Data.
(TIFF)

S9 Fig *efgB*^*evo*^ alleles increase resistance to low concentrations of formaldehyde even when EfgA is functional. Growth of wild-type (CM2730, circles), Δ*efgB* (CM3737, triangles), and *efgB*^*evo1*^ (CM3783, pink diamonds) and *efgB*^*evo2*^ mutant (CM3837, green diamonds) was quantified in liquid MP medium (methanol) containing 2 mM formaldehyde. Error bars represent the standard error of the mean for three biological replicates. The original data shown in this and all other figures are available in Supporting information file S1 Data.
(TIFF)

S10 Fig The Δ*efgA* mutant does not have additional resistance to alternative aldehydes. Growth of wild-type (CM2730, circles) and the Δ*efgA* mutant (CM3745, triangles) was quantified in liquid MP medium (succinate) containing no aldehydes (panel A, gray symbols). Additionally, growth of wild-type (black circles) and the Δ*efgA* mutant (blue triangles) was quantified in the same medium with the addition of (A) 2 mM formaldehyde, (B) 1.25 mM acetaldehyde, (C) 2.5 mM butyraldehyde, (D) 2.5 mM propionaldehyde, (E) 1.25 mM glyoxal, and (F) 0.157 mM glutaraldehyde. Error bars represent the standard error of mean of three biological replicates. The original data shown in this and all other figures are available in Supporting information file S1 Data.
(TIFF)

S11 Fig *efgB*^*evo*^ alleles confer resistance to a variety of stressors. (A) Growth of wild-type (CM2730, circles) and the Δ*efgB* mutant (CM3737, triangles) were quantified in methanol medium with the addition of no aldehydes (gray symbols), 2.5 mM butyraldehyde (purple symbols), 1.25 mM glyoxal (red symbols), and 0.157 mM glutaraldehyde (blue symbols). (B) Viability of wild-type (CM2730, circles), Δ*efgB* (CM3737, triangles), and *efgB*^*evo1*^ (CM3783, pink diamonds) and *efgB*^*evo2*^ mutant (CM3837, green diamonds) was assayed when culture tubes were submerged in a 55°C water bath for 0, 5, or 10 m. Error bars represent the standard error of the mean for three biological replicates. (C) Disc-diffusion assays were performed by placing antibiotic-impregnated discs upon soft agar overlays of *M*. *extorquens* on solid MP media (15 mM succinate). The zones of inhibition showed that *efgB*^*evo1*^ (CM3783, pink) and *efgB*^*evo2*^ mutants (CM3837, green) are more resistant to multiple antibiotics than the wild-type (CM2730, black) and the Δ*efgB* mutant (CM3737, white). KN, kanamycin; NA, nalidixic acid; NB, novobiocin; RA, rifampicin; TC, tetracycline. The original data shown in this and all other figures are available in Supporting information file S1 Data.
(TIFF)

S12 Fig Protein topology of EfgA. The secondary structural elements of EfgA and their relative positions are shown. The peptide chain begins at the N-terminus (“N”) and proceeds through the C-terminus (“C”); the directionality is indicated by the small blue arrows. Cylinders represent ɑ-helices, and the wide arrows represent the β-strands. Residue numbers that begin and end each element are noted.
(TIFF)

S13 Fig Evolved *efgA* alleles have a variety of predicted folding and binding stabilities. We used our MD+FoldX approach [79] to predict the effect of all possible 19 mutations at each amino acid site on monomers or tetramer formation. (A) The distribution of ΔΔG values associated to monomer folding and tetramer binding for all possible nonsynonymous mutations (2,546) of *efgA* are shown as gray circles. Orange circles indicate the location of the experimentally observed mutations (14) within the distribution. The green circle indicates the location of the S114N mutation. (B) A table of the 14 experimentally observed amino acid substitutions with their ΔΔG folding and ΔΔG binding values listed (in kcal/mol). Of these, 10 mutations increased the folding free energy of the monomer, suggesting they decreased the monomer stability and one was predicted to significantly increase binding free energy associated to tetramer formation, suggesting it destabilized the oligomeric assembly. The original data shown in this and all other figures are available in Supporting information file S1 Data.
(TIFF)

S14 Fig Formaldehyde-binding residues are largely conserved in DUF336 homologs. A Clustal Omega [134] alignment was performed with EfgA, structural DUF336 homologs (bold) and additional homologs referenced in the text. Conservation of residues is indicated when identical (*), strongly similar (:), or weakly similar (.). Small-hydrophobic residues (less Y) are in red (AVFPMILW), acidic residues are in blue (DE), basic residues are in magenta (RHK), and hydroxly + sulfhydryl + amine + G residues are in green (STYHCNGQ). Boxes indicate residues that correspond to those implicated in formaldehyde binding in EfgA (K57, S114, and D121). Pairwise comparisons between EfgA and each homolog were performed with EMBOSS Needle [135] to ascertain % identities and % similarities. For PduO, only the C-terminal DUF336 domain (PDB:5CX7) was included.
(TIFF)

S15 Fig Replicates of microscale isothermal calorimetry assays of EfgA:ligand binding. The binding isotherms represented as heat change (μJ/s) upon injection over time are in the top portion of the split graphs, with independent binding modeling on the bottom portion. Binding observed with 50 μM EfgA (A) and 2 μL injections of 25 mM formaldehyde (in PBS). Binding observed with 50 μM EfgA and 2 μL injections of methanol (C), formate (D), and acetaldehyde (E). Data are experimental replicates (*n =* 3) performed with protein from three independent purifications. The original data shown in this and all other figures are available in Supporting information file S1 Data.
(TIFF)

S16 Fig Microscale thermophoresis indicates EfgA binds formaldehyde. The dose response curve of EfgA to formaldehyde is represented by the difference in normalized fluorescence (F_norm_ [‰]) for analysis of thermophoresis across formaldehyde concentrations with 20 nM EfgA. The K_d_ is fitted to 8.01 ± 3.5 mM. Data represent (*n* = 3) MST measurements. The original data shown in this and all other figures are available in Supporting information file S1 Data.
(TIFF)

S17 Fig Endogenous formaldehyde is elevated when dH_4_MPT pathway is disrupted. Intracellular formaldehyde levels of wild-type (black), Δ*fae* (CM3753, orange) were measured by Purpald assay. Strains were grown in liquid MP medium (succinate) to early exponential phase at which point 1 mM methanol was introduced into the medium (t = 0 h); formaldehyde was measured at t = 4 h. Error bars represent the standard error of the mean for three biological replicates. The original data shown in this and all other figures are available in Supporting information file S1 Data.
(TIFF)

S18 Fig Growth of methanol sensitive mutants is comparable to wild type during growth on succinate. Wild-type and mutant strains were grown in liquid MP medium (succinate). Strains represented are wild-type (black circles), Δ*fae* (CM3753, orange squares), Δ*mptG* (CM4765, light blue squares), Δ*efgA* Δ*fae* (CM3421-5, green hexagons), and Δ*efgA* Δ*mptG* mutants (CM3440-13, blue hexagons). Error bars represent the standard error of the mean for three biological replicates. The original data shown in this and all other figures are available in Supporting information file S1 Data.
(TIFF)

S19 Fig Relative fitness of *efgA* and alleles at other loci. Strains with evolved beneficial alleles that independently conferred formaldehyde growth were assessed for fitness in media containing 5 mM formaldehyde. Relative fitness values were determined via competition experiments against a common fluorescently tagged reference strain. Fitness values for each strain, relative to the *efgA*^*evo1*^ mutant, are presented as bars representing mean +/− SEM (*n* = 3 biological replicates). Statistical significance was determined by an unpaired Student *t* test (*, *p* < 0.05). The original data shown in this and all other figures are available in Supporting information file S1 Data.
(TIFF)

S1 Table Bacterial strains and plasmids. (XLSX)

S2 Table Expression of genes targeted in evolution of formaldehyde growth evolution are not differentially expressed in formaldehyde tolerant subpopulations. (XLSX)

S1 Data Relative fitness of *efgA* and alleles at other loci. Excel file with original data shown in Figs 3–5, 7–9, and 10–13 and S2–S5, S6, S8–S11, S13, and S15–S19 Figs.
(XLSX)

## Abbreviations

ANL APS: Argonne National Laboratory’s Advanced Photon Source
CFU: colony-forming unit
dH_4_MPT: dephospho-tetrahydromethanopterin
DMF: dimethylformamide
DTT: dithiothreitol
DUF: domain of unknown function
Fae: formaldehyde-activating enzyme
fMet: *N*-formylmethionine
GSH: glutathione
IPTG: isopropyl β-D-1-thiogalactopyranoside
MCL: Maximum Composite Likelihood
MD: molecular dynamics
Met: methionine
mITC: microscale isothermal calorimetry
MP: *Methylobacterium* PIPES
MST: microscale thermophoresis
PDB: Protein Data Bank
PDF: peptide deformylase
rmsd: root-mean-square deviations
SDS–PAGE: sodium dodecyl sulfate polyacrylamide gel electrophoresis
