# Supplementary figures and images for: EfgA is a conserved formaldehyde sensor that leads to bacterial growth arrest in response to elevated formaldehyde
Source: PLoS Biol. 2021 May 26;19(5):e3001208. doi: 10.1371/journal.pbio.3001208 (PMC8153426; doi:10.1371/journal.pbio.3001208)

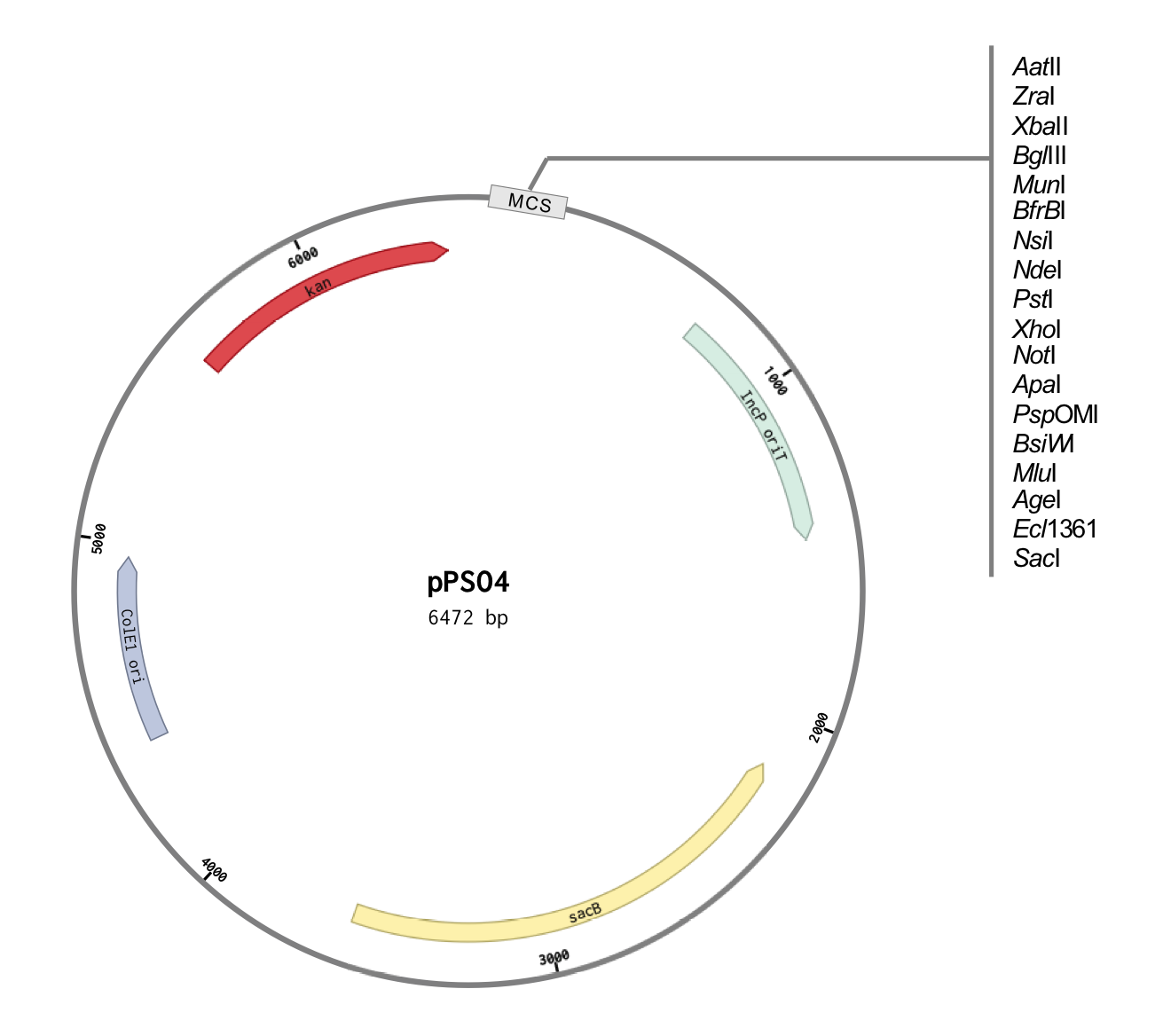

Supplement: S1 Fig — Plasmid map of pPS04 [GenBank: MW118672] showing the key features including kan (encodes kanamycin resistance), multiple cloning site (MCS) containing a number of single-cutting restriction sites, IncP oriT (origin of conjugal transfer), sacB (encodes levansucrase for sucrose sensitivity), ColE1 ori (high-copy origin of replication for E. coli). (TIFF) [file pbio.3001208.s001.tiff]

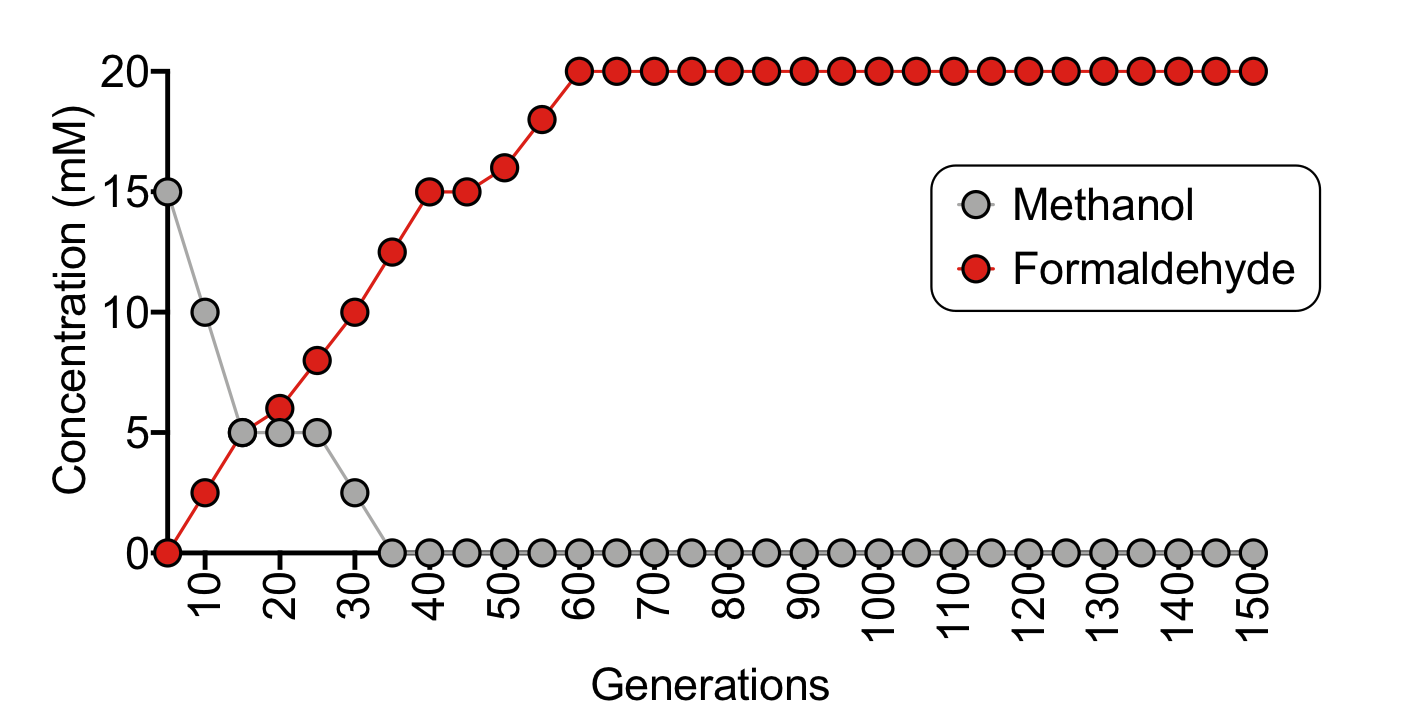

Supplement: S2 Fig — M. extorquens was experimentally evolved in Hypho liquid media for 150 generations. Initial growth conditions relied on methanol as a sole source of carbon and energy. At each transfer (approximately 6 generations), methanol concentrations were decreased and formaldehyde was introduced into the growth media at increasing concentrations. By generation 35, formaldehyde was the only carbon/energy source present. Formaldehyde concentrations continued to be gradually increased until generation 60 when it reached 20 mM. Selective pressure was sustained at 20 mM formaldehyde until the experiment was completed at 150 generations. The original data shown in this and all other figures are available in Supporting information file S1 Data. (TIFF) [file pbio.3001208.s002.tiff]

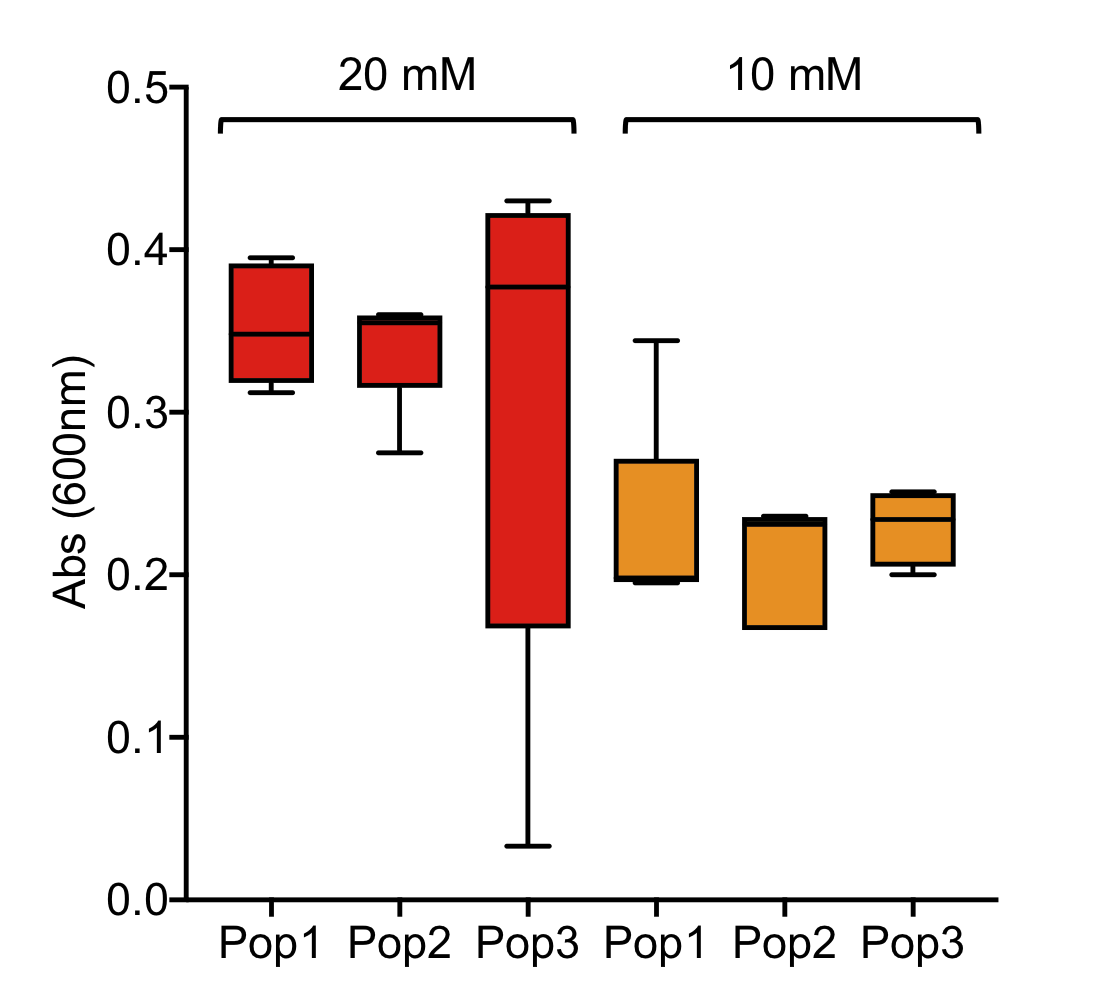

Supplement: S3 Fig — Five isolates from each of the final formaldehyde-evolved populations (CM3031-CM3045, Table 1) were grown in minimal Hypho medium with 20 mM or 10 mM formaldehyde for 48 h. Plot whiskers indicate the minimum and maximum values. The original data shown in this and all other figures are available in Supporting information file S1 Data. (TIFF) [file pbio.3001208.s003.tiff]

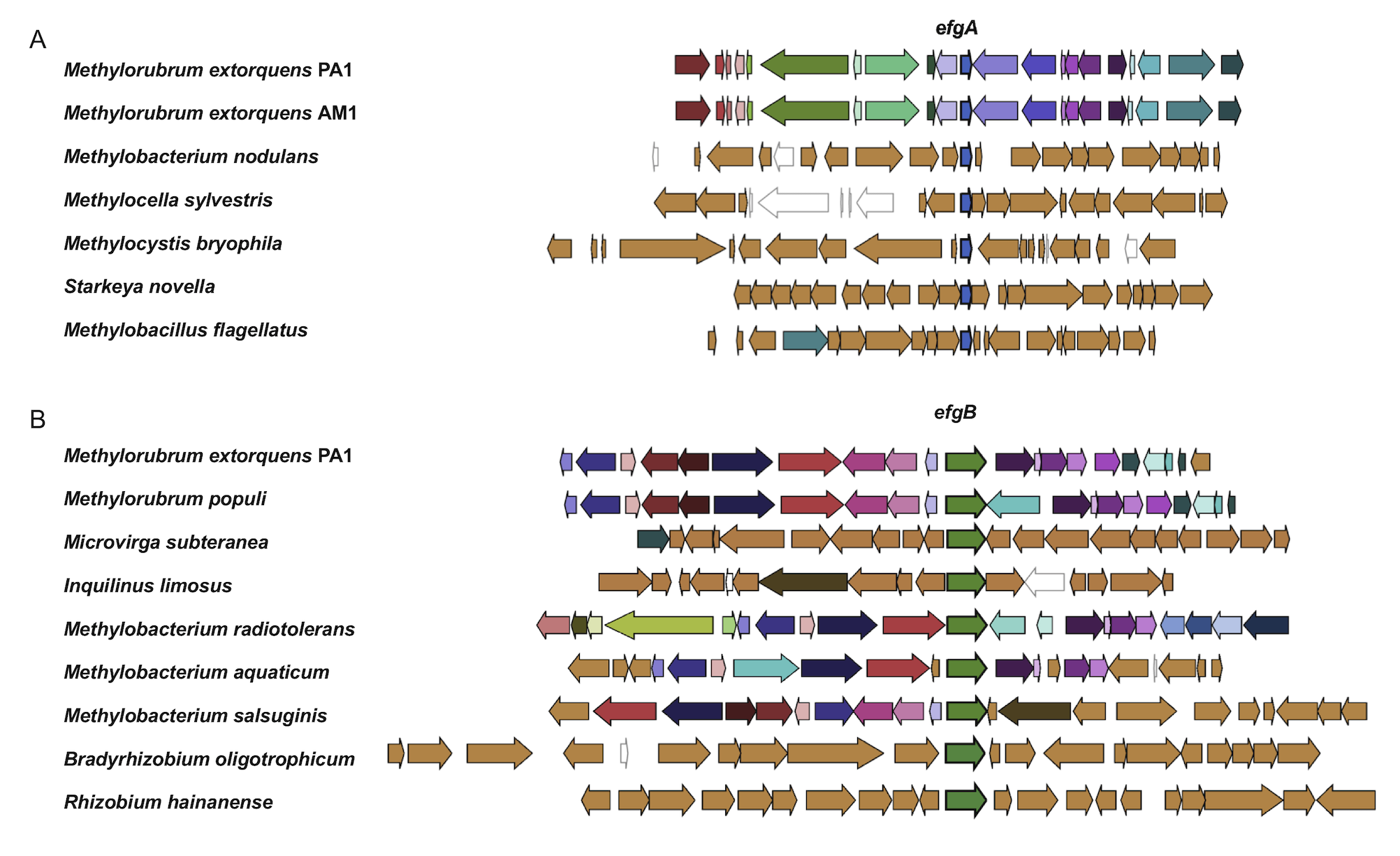

Supplement: S4 Fig — Gene neighborhoods from fully assembled genomes are aligned by (A) efgA (blue) or (B) efgB (green) and include 10 flanking genes on either side. Organisms represented in Panel A span the EfgA-clade (Fig 2) identified in the phylogenetic analysis of EfgA homologs, while those in Panel B span the phylogenetic distribution of EfgB homolog containing organisms (S7 Fig). Homology between genes is indicated by identical colors among the genomes within each panel. In both panels, genes lacking homologous counterparts in the gene neighborhoods shown are colored tan. (TIFF) [file pbio.3001208.s004.tiff]

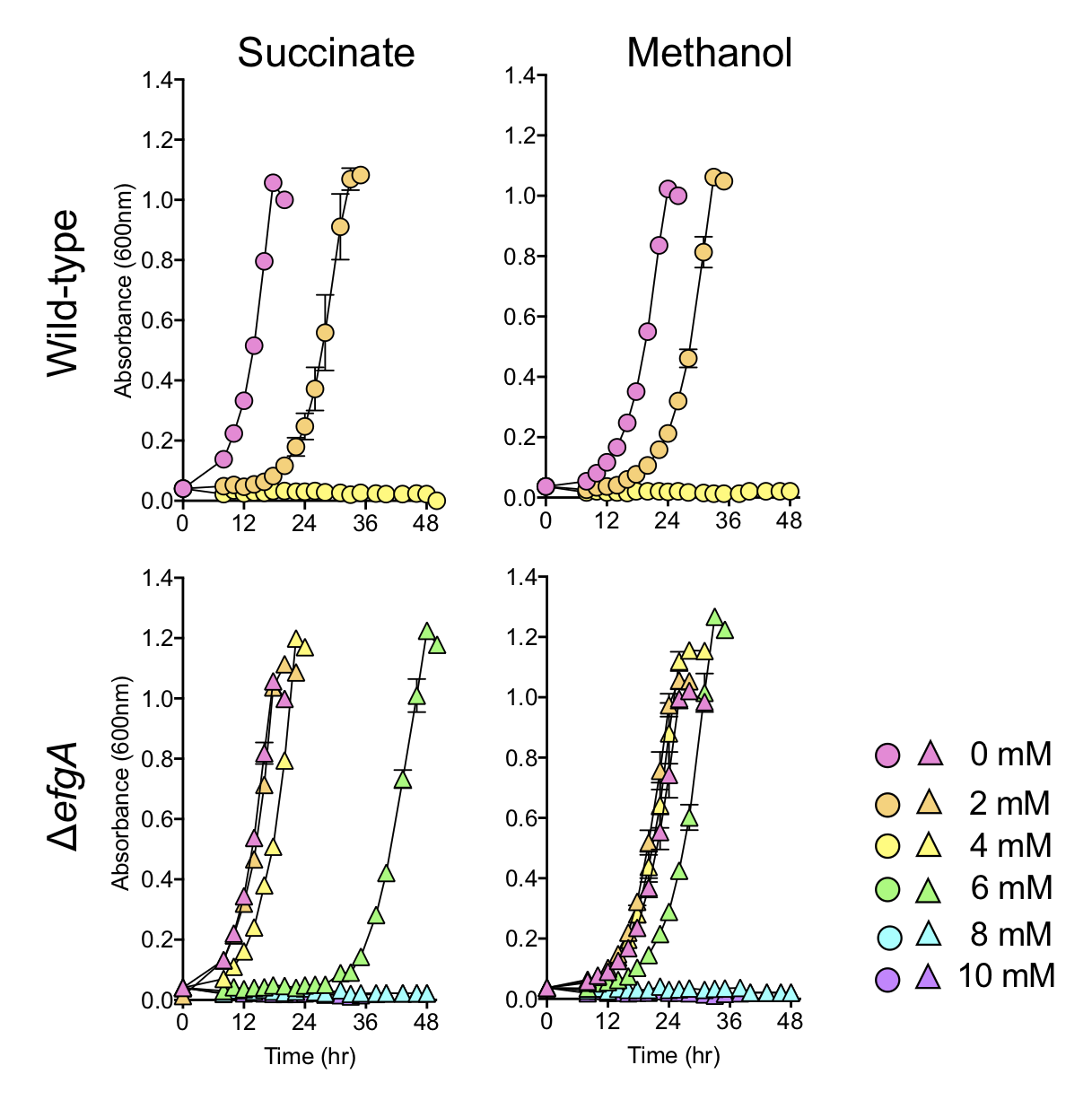

Supplement: S5 Fig — Wild-type (CM2730, circles, upper panels) and the ΔefgA mutant (CM3745, triangles, lower panels) were grown in liquid MP medium with succinate (left panels) or methanol (right panels) provided as the primary carbon source. Additionally, 0, 2, 4, 6, 8, or 10 mM exogenous formaldehyde was provided as a stressor; however, it can also serve as a secondary carbon source when it is tolerated. Error bars represent the standard error of the mean of three biological replicates. The original data shown in this and all other figures are available in Supporting information file S1 Data. (TIFF) [file pbio.3001208.s005.tiff]

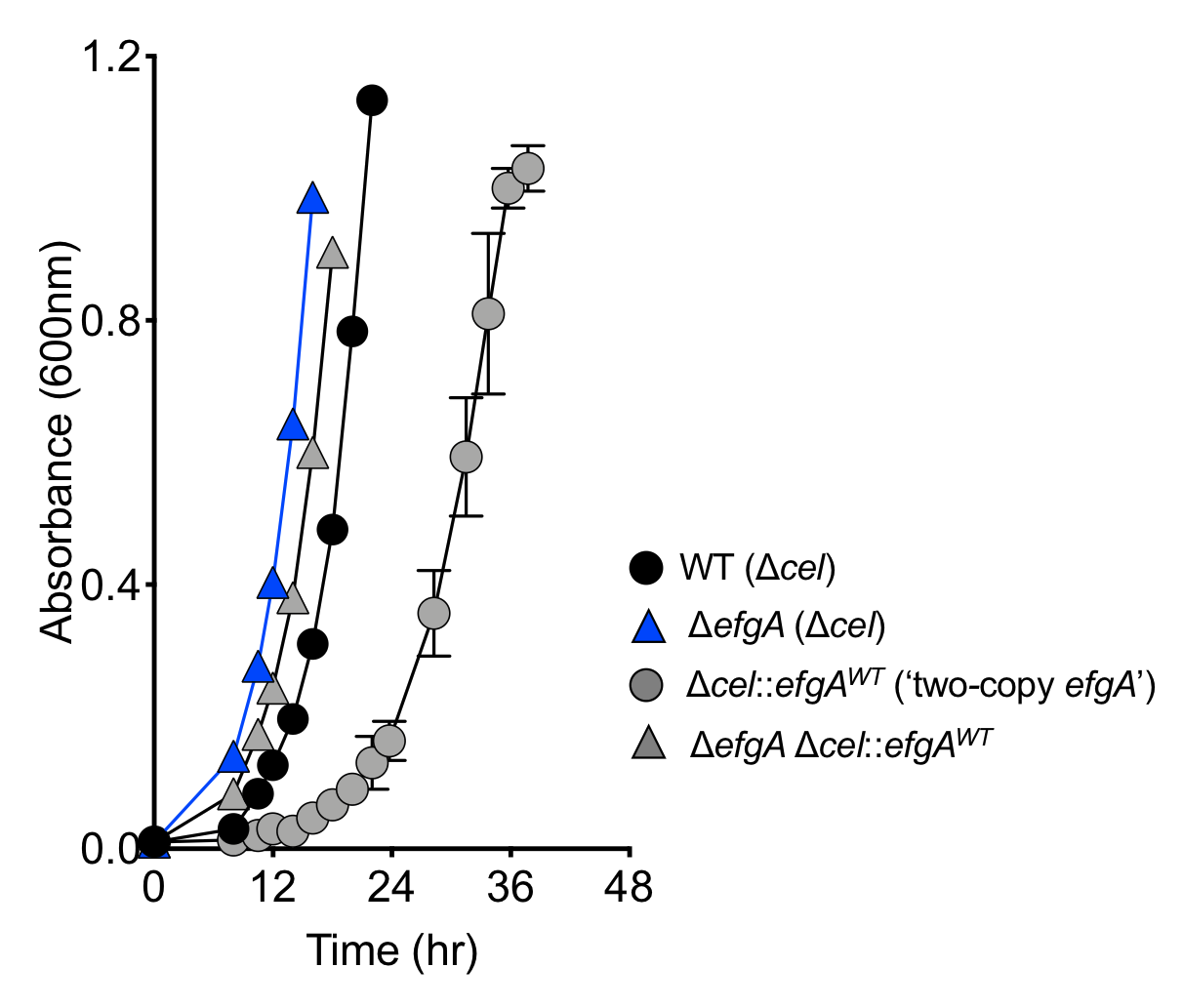

Supplement: S6 Fig — The wild-type (black circles), ΔefgA mutant (blue triangles), two-copy efgA mutant (gray circles), and ΔefgA+chromosomal efgA complement (gray triangles) were grown in liquid MP medium with 3.5 mM succinate and 2 mM formaldehyde. Error bars represent the standard error of mean of three biological replicates. All strains were derived from WT, which is Δcel; the second copy of efgA was introduced at the Δcel locus. The original data shown in this and all other figures are available in Supporting information file S1 Data. (TIFF) [file pbio.3001208.s006.tiff]

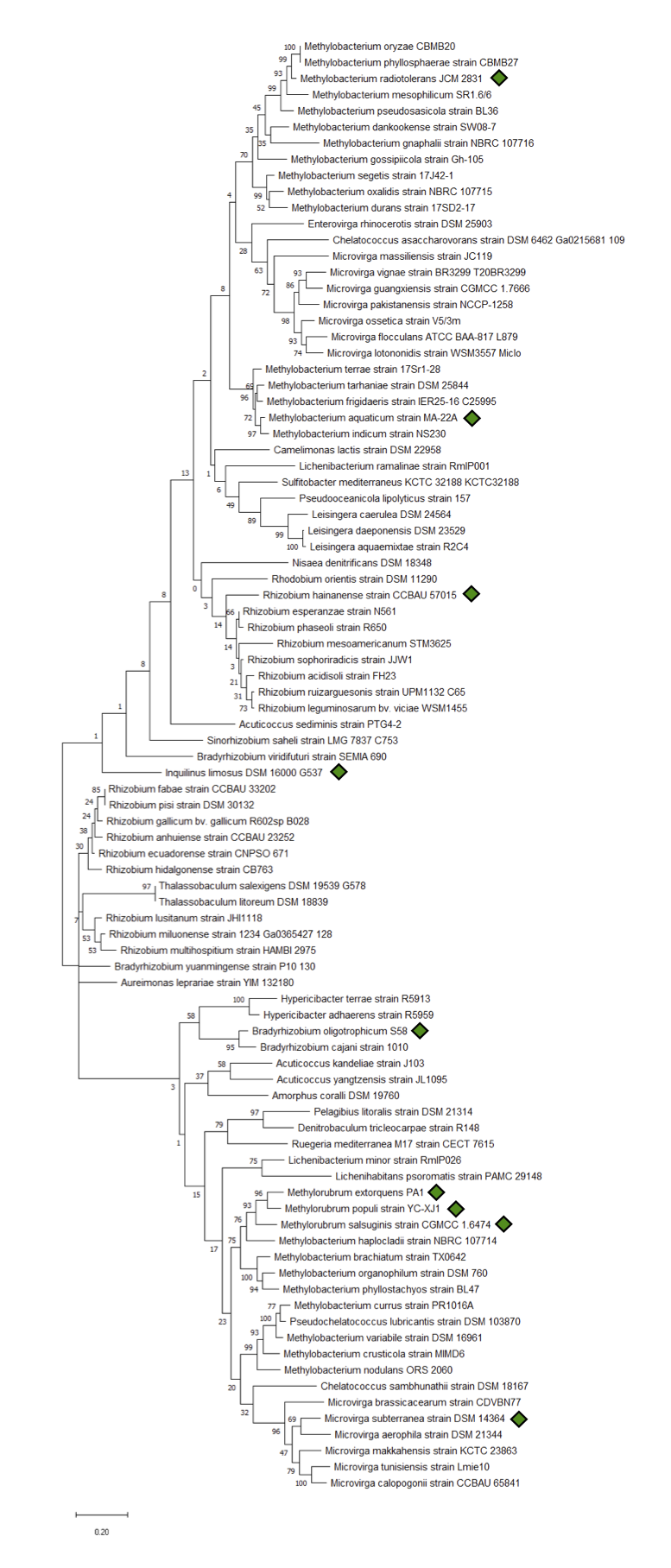

Supplement: S7 Fig — The evolutionary relationship of efgB was compared to genes with 65%–90% identity via maximum likelihood. Bootstrap values are shown at nodes, and branch lengths reflect the indicate substitutions per nucleotide. The green diamonds represent members whose genomic context is illustrated in S4 Fig. The phylogenetic data are available at TreeBASE (http://purl.org/phylo/treebase/phylows/study/TB2:S27073). (TIFF) [file pbio.3001208.s007.tiff]

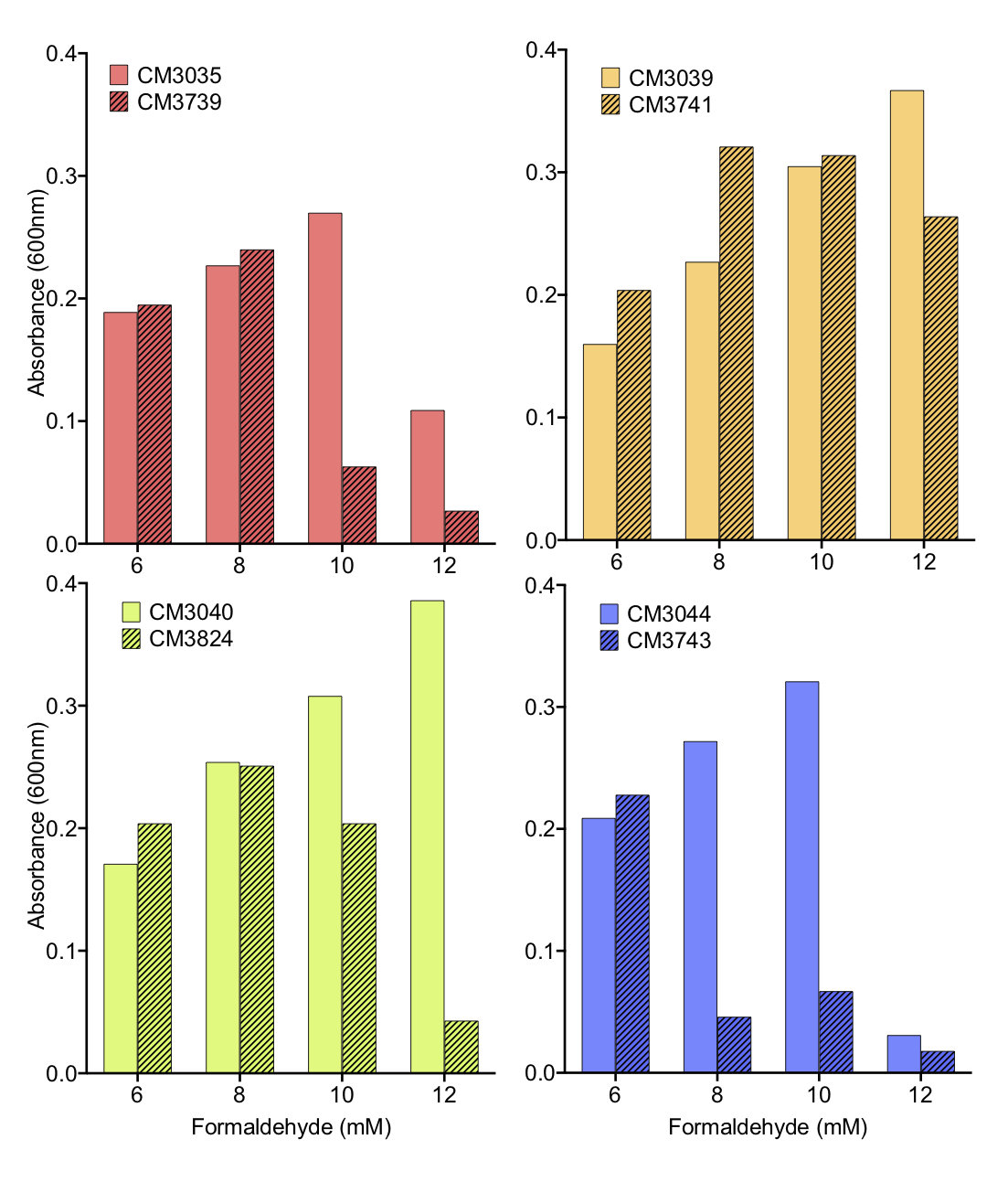

Supplement: S8 Fig — Formaldehyde growth of evolved isolates CM3035 (pink), CM3039 (orange), CM3040 (green), and CM3044 (violet) was measured in liquid MP medium with 6, 8, 10, or 12 mM exogenous formaldehyde provided as a sole source of carbon and energy. Replacing evolved beneficial efgB alleles with ΔefgB resulted in otherwise isogenic strains CM3739, CM3741, CM3824, and CM3743 (hatched bars) failed to utilize formaldehyde at higher concentrations. Data are representative of trends observed in multiple experiments. The original data shown in this and all other figures are available in Supporting information file S1 Data. (TIFF) [file pbio.3001208.s008.tiff]

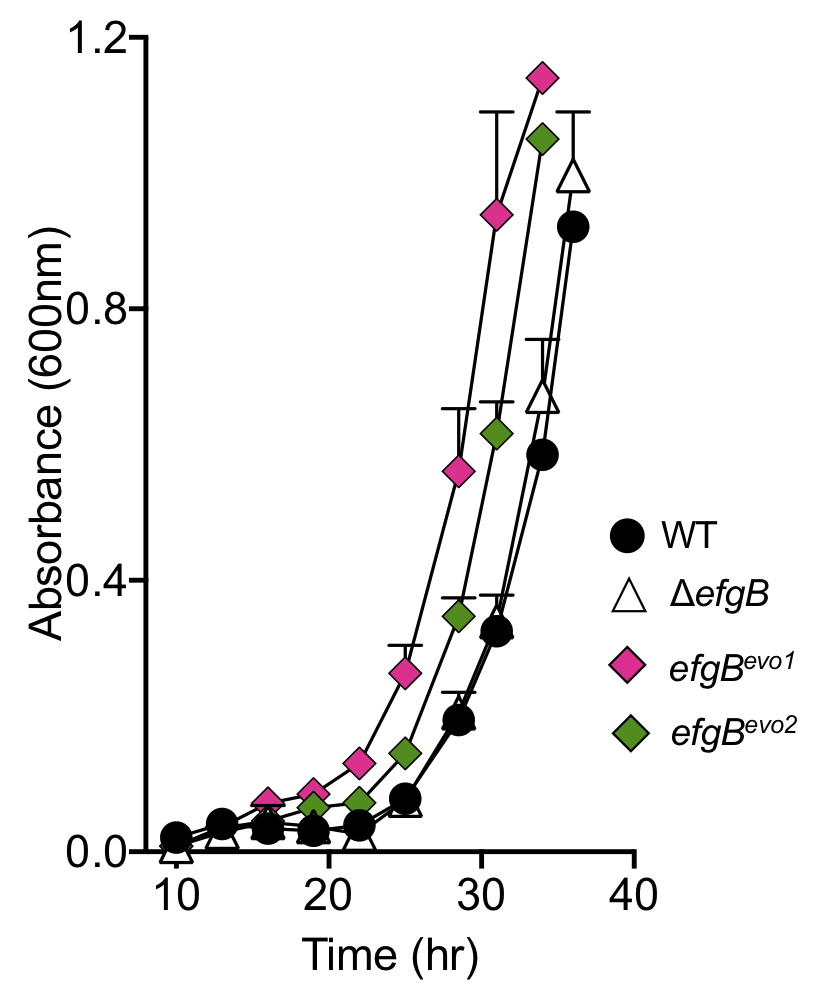

Supplement: S9 Fig — Growth of wild-type (CM2730, circles), ΔefgB (CM3737, triangles), and efgBevo1 (CM3783, pink diamonds) and efgBevo2 mutant (CM3837, green diamonds) was quantified in liquid MP medium (methanol) containing 2 mM formaldehyde. Error bars represent the standard error of the mean for three biological replicates. The original data shown in this and all other figures are available in Supporting information file S1 Data. (TIFF) [file pbio.3001208.s009.tiff]

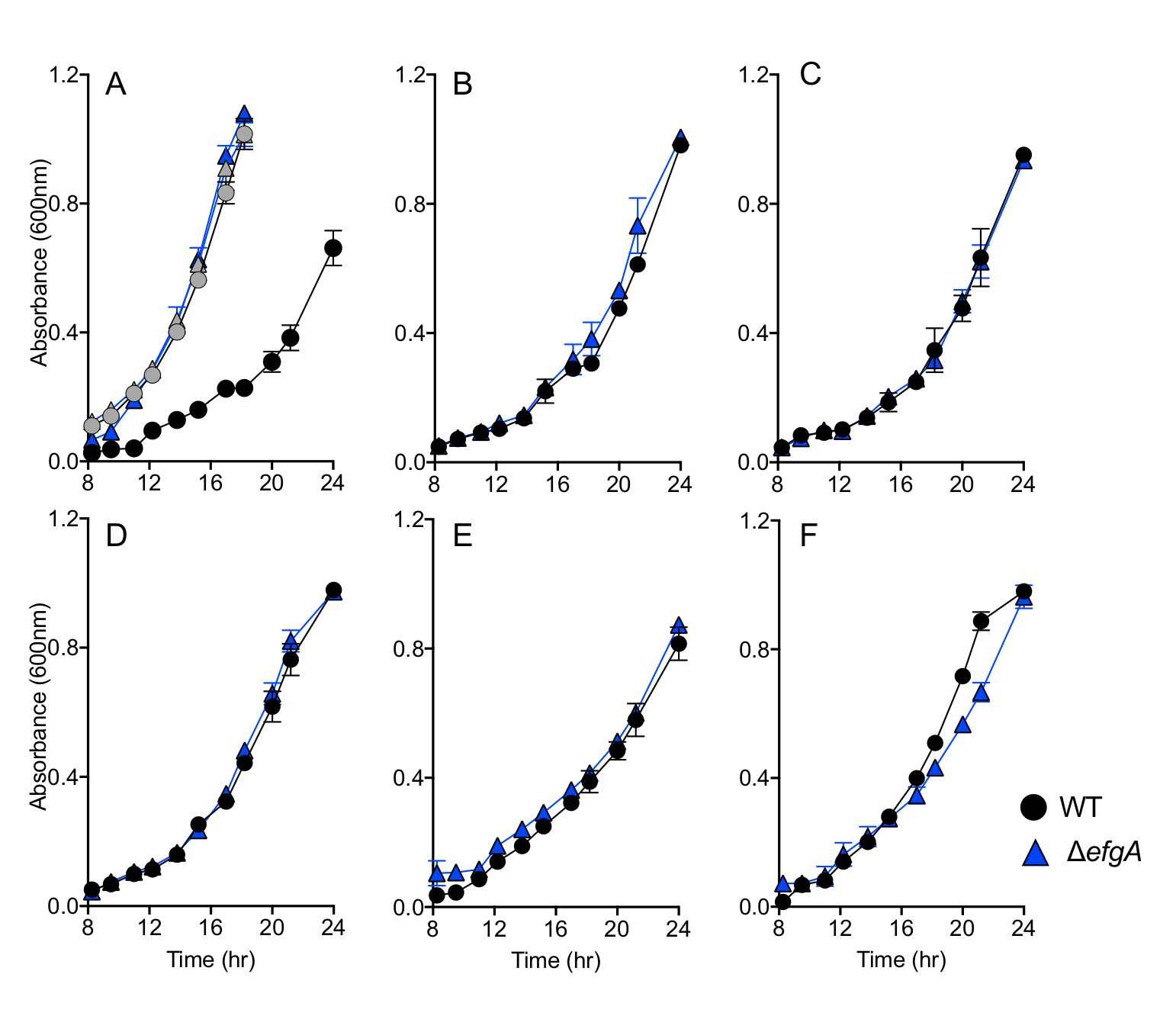

Supplement: S10 Fig — Growth of wild-type (CM2730, circles) and the ΔefgA mutant (CM3745, triangles) was quantified in liquid MP medium (succinate) containing no aldehydes (panel A, gray symbols). Additionally, growth of wild-type (black circles) and the ΔefgA mutant (blue triangles) was quantified in the same medium with the addition of (A) 2 mM formaldehyde, (B) 1.25 mM acetaldehyde, (C) 2.5 mM butyraldehyde, (D) 2.5 mM propionaldehyde, (E) 1.25 mM glyoxal, and (F) 0.157 mM glutaraldehyde. Error bars represent the standard error of mean of three biological replicates. The original data shown in this and all other figures are available in Supporting information file S1 Data. (TIFF) [file pbio.3001208.s010.tiff]

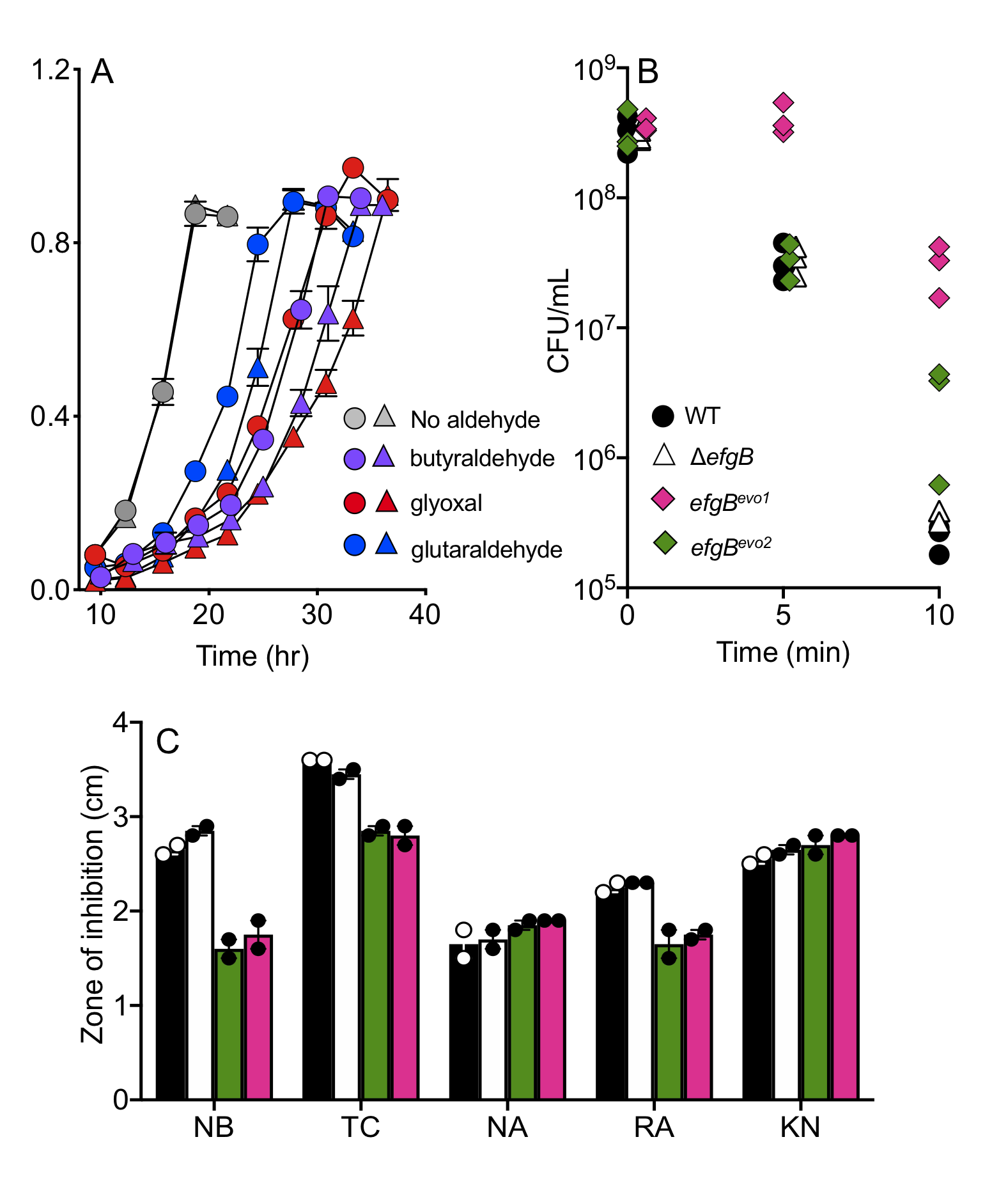

Supplement: S11 Fig — (A) Growth of wild-type (CM2730, circles) and the ΔefgB mutant (CM3737, triangles) were quantified in methanol medium with the addition of no aldehydes (gray symbols), 2.5 mM butyraldehyde (purple symbols), 1.25 mM glyoxal (red symbols), and 0.157 mM glutaraldehyde (blue symbols). (B) Viability of wild-type (CM2730, circles), ΔefgB (CM3737, triangles), and efgBevo1 (CM3783, pink diamonds) and efgBevo2 mutant (CM3837, green diamonds) was assayed when culture tubes were submerged in a 55°C water bath for 0, 5, or 10 m. Error bars represent the standard error of the mean for three biological replicates. (C) Disc-diffusion assays were performed by placing antibiotic-impregnated discs upon soft agar overlays of M. extorquens on solid MP media (15 mM succinate). The zones of inhibition showed that efgBevo1 (CM3783, pink) and efgBevo2 mutants (CM3837, green) are more resistant to multiple antibiotics than the wild-type (CM2730, black) and the ΔefgB mutant (CM3737, white). KN, kanamycin; NA, nalidixic acid; NB, novobiocin; RA, rifampicin; TC, tetracycline. The original data shown in this and all other figures are available in Supporting information file S1 Data. (TIFF) [file pbio.3001208.s011.tiff]

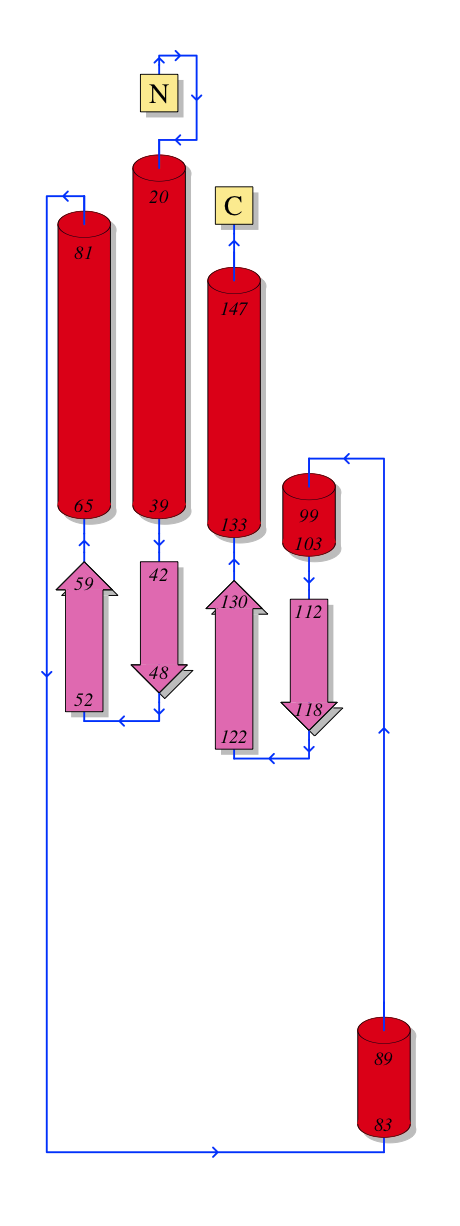

Supplement: S12 Fig — The secondary structural elements of EfgA and their relative positions are shown. The peptide chain begins at the N-terminus (“N”) and proceeds through the C-terminus (“C”); the directionality is indicated by the small blue arrows. Cylinders represent ɑ-helices, and the wide arrows represent the β-strands. Residue numbers that begin and end each element are noted. (TIFF) [file pbio.3001208.s012.tiff]

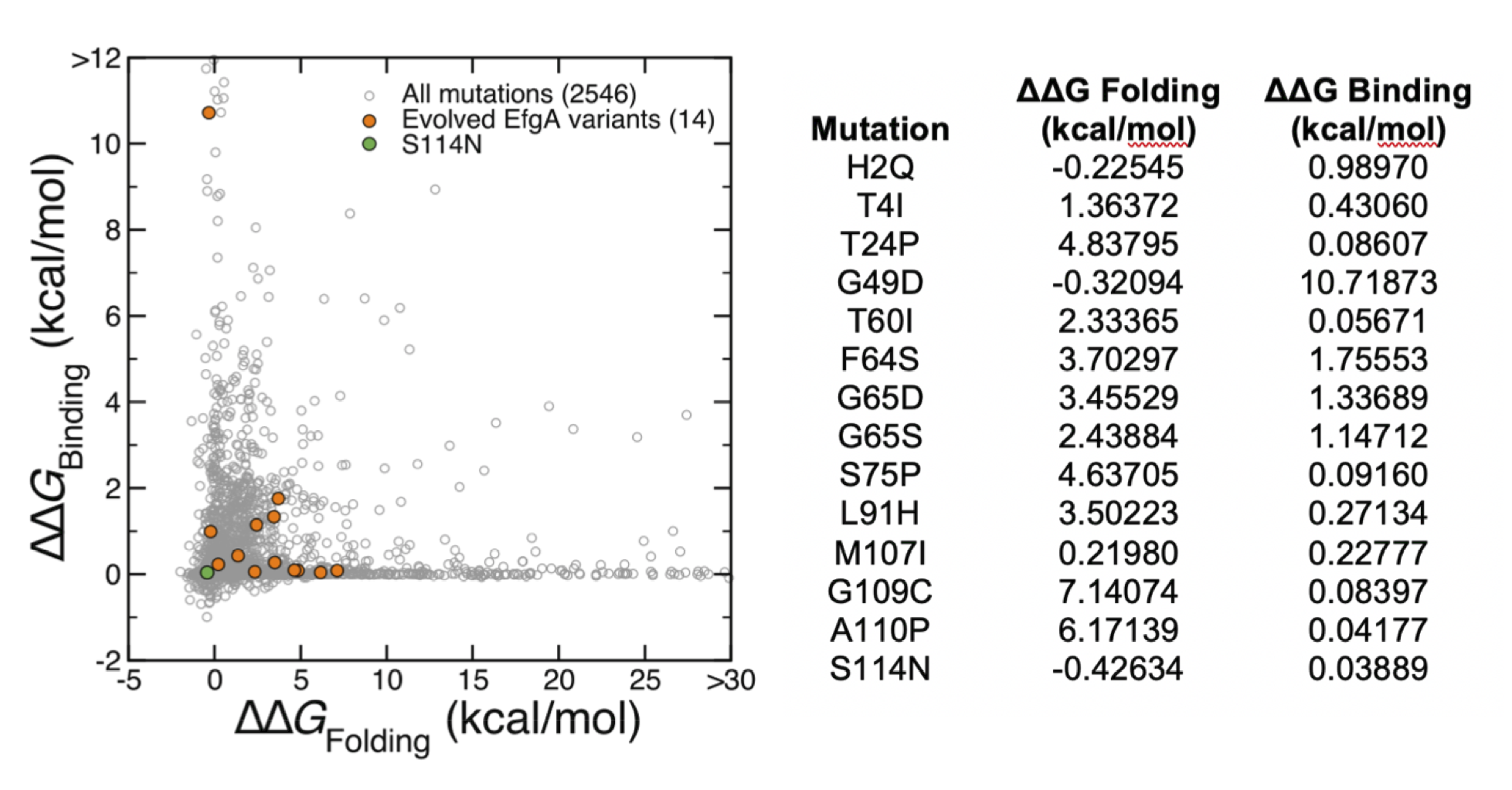

Supplement: S13 Fig — We used our MD+FoldX approach [79] to predict the effect of all possible 19 mutations at each amino acid site on monomers or tetramer formation. (A) The distribution of ΔΔG values associated to monomer folding and tetramer binding for all possible nonsynonymous mutations (2,546) of efgA are shown as gray circles. Orange circles indicate the location of the experimentally observed mutations (14) within the distribution. The green circle indicates the location of the S114N mutation. (B) A table of the 14 experimentally observed amino acid substitutions with their ΔΔG folding and ΔΔG binding values listed (in kcal/mol). Of these, 10 mutations increased the folding free energy of the monomer, suggesting they decreased the monomer stability and one was predicted to significantly increase binding free energy associated to tetramer formation, suggesting it destabilized the oligomeric assembly. The original data shown in this and all other figures are available in Supporting information file S1 Data. (TIFF) [file pbio.3001208.s013.tiff]

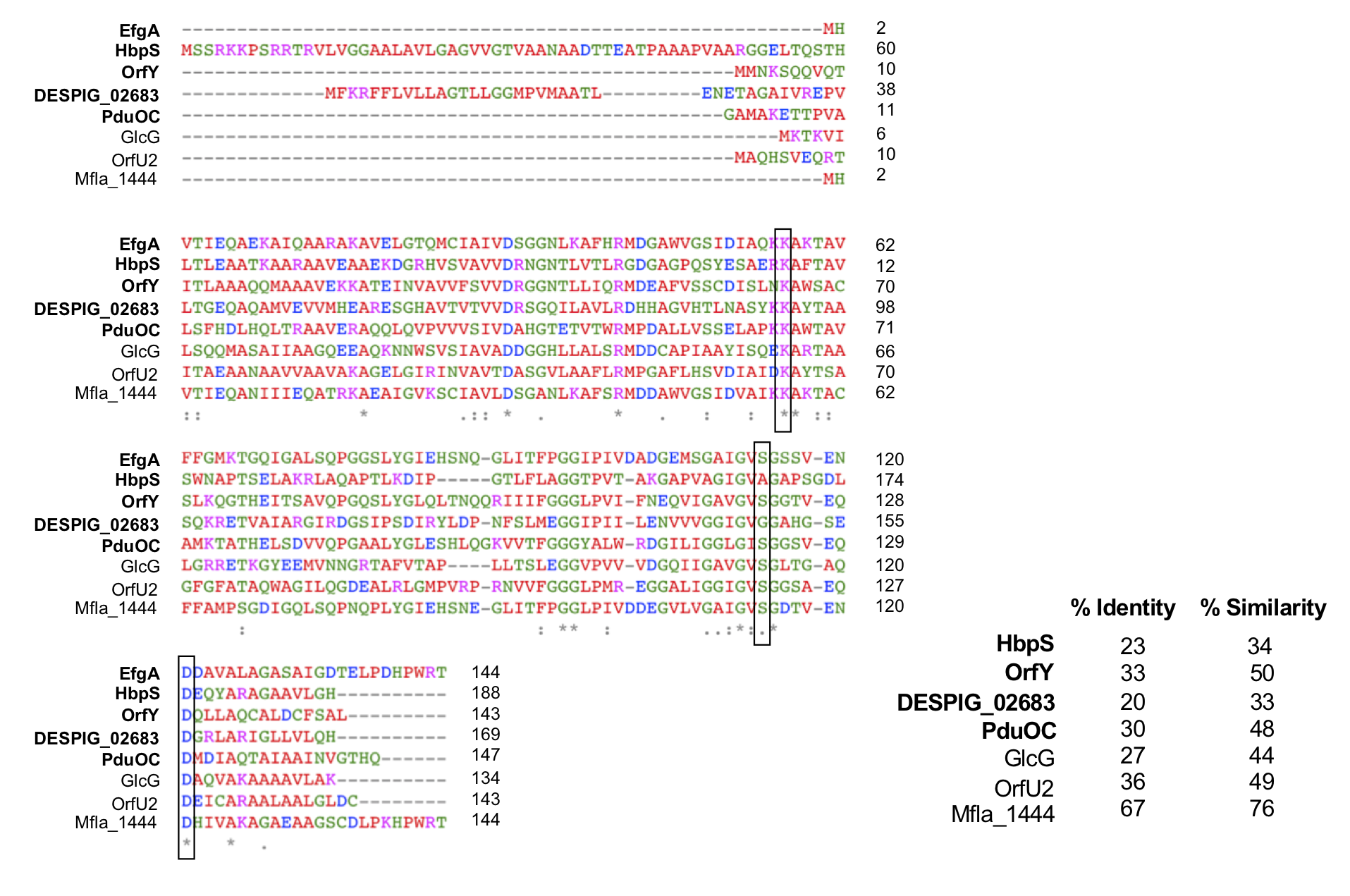

Supplement: S14 Fig — A Clustal Omega [134] alignment was performed with EfgA, structural DUF336 homologs (bold) and additional homologs referenced in the text. Conservation of residues is indicated when identical (*), strongly similar (:), or weakly similar (.). Small-hydrophobic residues (less Y) are in red (AVFPMILW), acidic residues are in blue (DE), basic residues are in magenta (RHK), and hydroxly + sulfhydryl + amine + G residues are in green (STYHCNGQ). Boxes indicate residues that correspond to those implicated in formaldehyde binding in EfgA (K57, S114, and D121). Pairwise comparisons between EfgA and each homolog were performed with EMBOSS Needle [135] to ascertain % identities and % similarities. For PduO, only the C-terminal DUF336 domain (PDB:5CX7) was included. (TIFF) [file pbio.3001208.s014.tiff]

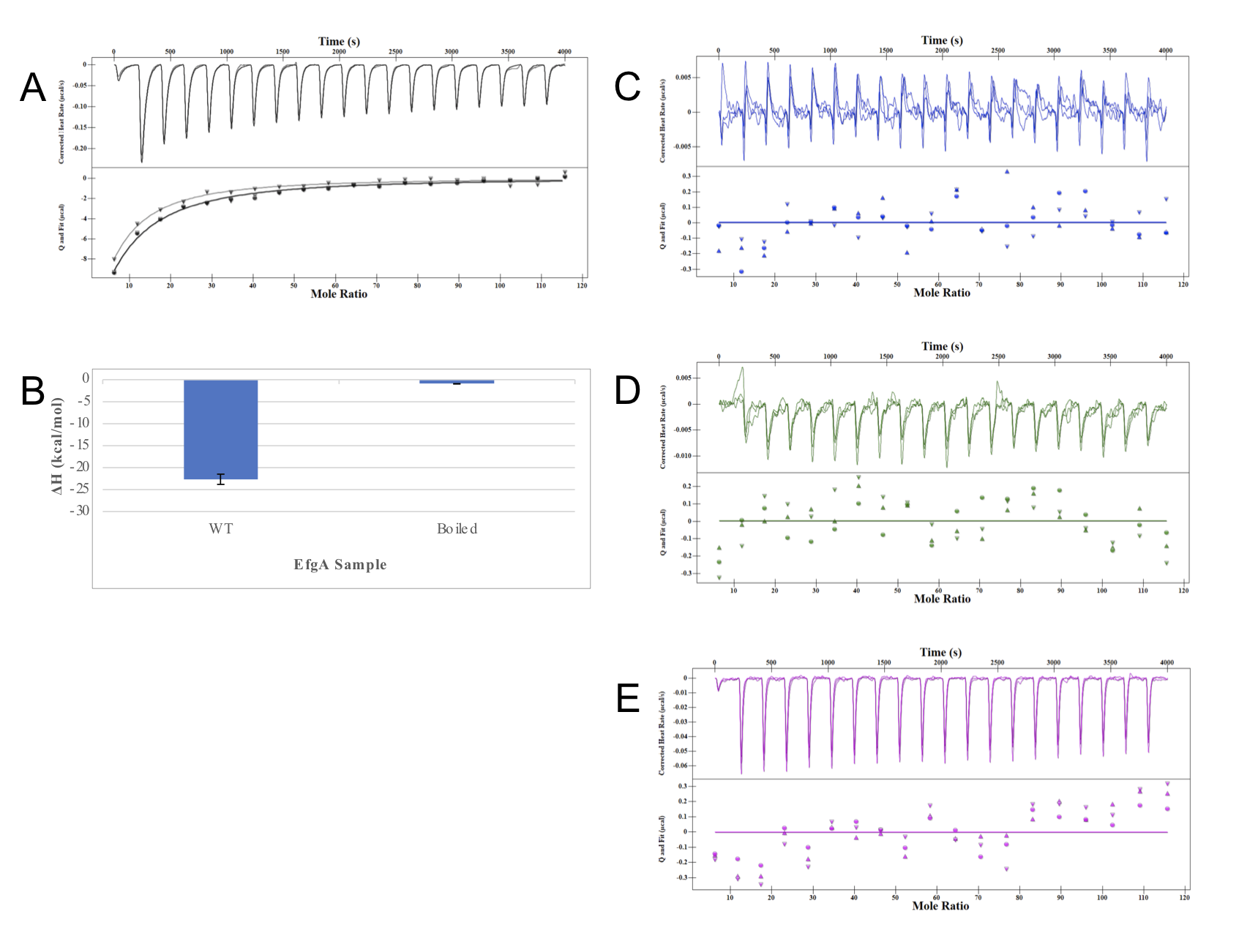

Supplement: S15 Fig — The binding isotherms represented as heat change (μJ/s) upon injection over time are in the top portion of the split graphs, with independent binding modeling on the bottom portion. Binding observed with 50 μM EfgA (A) and 2 μL injections of 25 mM formaldehyde (in PBS). Binding observed with 50 μM EfgA and 2 μL injections of methanol (C), formate (D), and acetaldehyde (E). Data are experimental replicates (n = 3) performed with protein from three independent purifications. The original data shown in this and all other figures are available in Supporting information file S1 Data. (TIFF) [file pbio.3001208.s015.tiff]

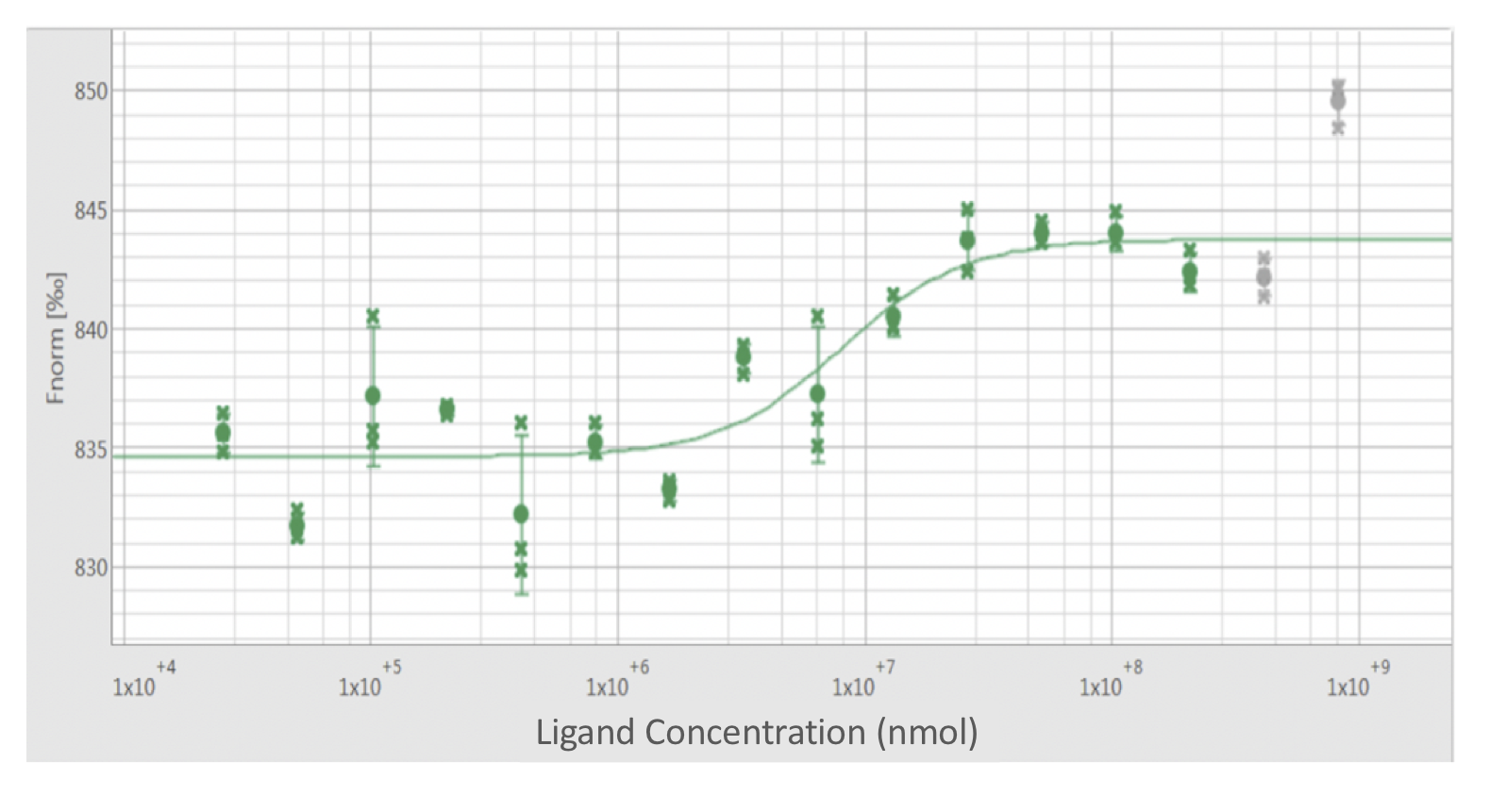

Supplement: S16 Fig — The dose response curve of EfgA to formaldehyde is represented by the difference in normalized fluorescence (Fnorm [‰]) for analysis of thermophoresis across formaldehyde concentrations with 20 nM EfgA. The Kd is fitted to 8.01 ± 3.5 mM. Data represent (n = 3) MST measurements. The original data shown in this and all other figures are available in Supporting information file S1 Data. (TIFF) [file pbio.3001208.s016.tiff]

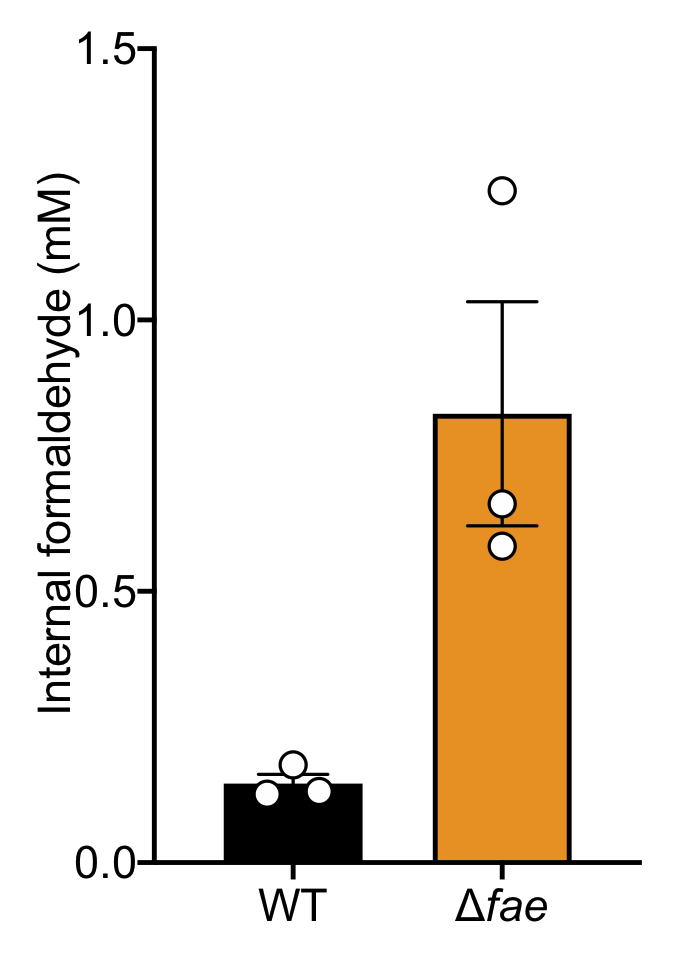

Supplement: S17 Fig — Intracellular formaldehyde levels of wild-type (black), Δfae (CM3753, orange) were measured by Purpald assay. Strains were grown in liquid MP medium (succinate) to early exponential phase at which point 1 mM methanol was introduced into the medium (t = 0 h); formaldehyde was measured at t = 4 h. Error bars represent the standard error of the mean for three biological replicates. The original data shown in this and all other figures are available in Supporting information file S1 Data. (TIFF) [file pbio.3001208.s017.tiff]

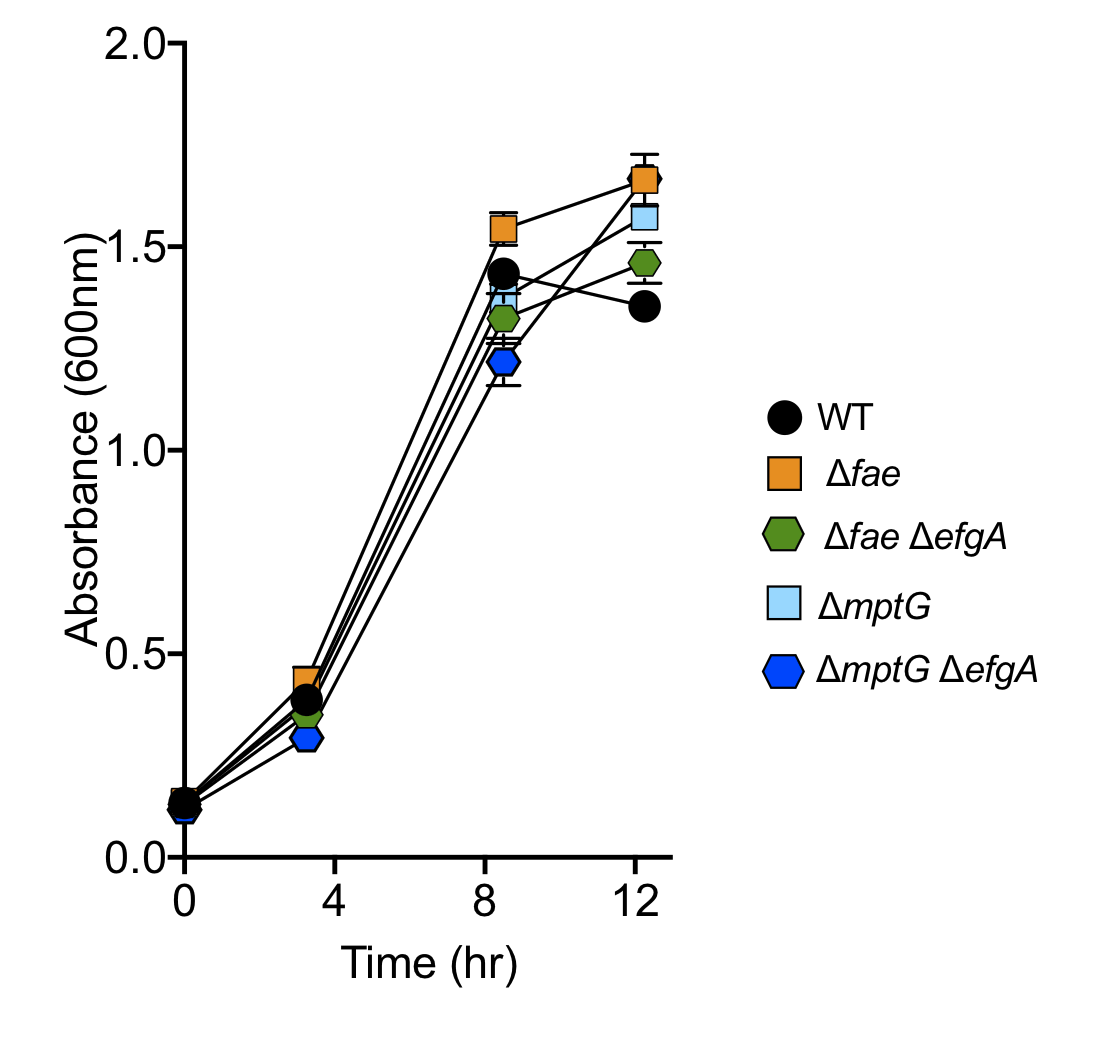

Supplement: S18 Fig — Wild-type and mutant strains were grown in liquid MP medium (succinate). Strains represented are wild-type (black circles), Δfae (CM3753, orange squares), ΔmptG (CM4765, light blue squares), ΔefgA Δfae (CM3421-5, green hexagons), and ΔefgA ΔmptG mutants (CM3440-13, blue hexagons). Error bars represent the standard error of the mean for three biological replicates. The original data shown in this and all other figures are available in Supporting information file S1 Data. (TIFF) [file pbio.3001208.s018.tiff]

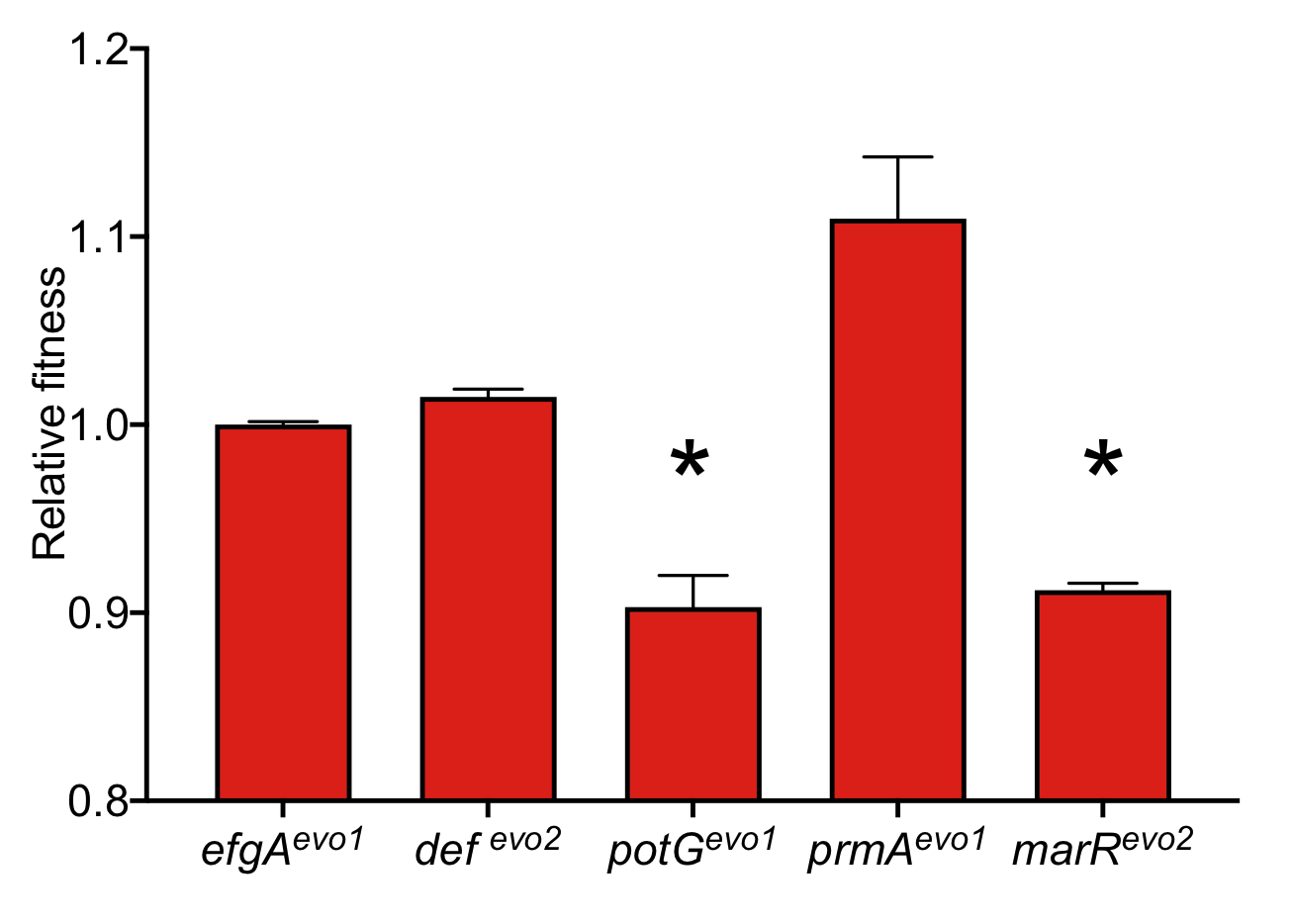

Supplement: S19 Fig — Strains with evolved beneficial alleles that independently conferred formaldehyde growth were assessed for fitness in media containing 5 mM formaldehyde. Relative fitness values were determined via competition experiments against a common fluorescently tagged reference strain. Fitness values for each strain, relative to the efgAevo1 mutant, are presented as bars representing mean +/− SEM (n = 3 biological replicates). Statistical significance was determined by an unpaired Student t test (*, p < 0.05). The original data shown in this and all other figures are available in Supporting information file S1 Data. (TIFF) [file pbio.3001208.s019.tiff]
